# Yb_2_‐Tb Upconversion in a Hetero‐Trimetallic Molecular Lanthanide Complex

**DOI:** 10.1002/anie.202519563

**Published:** 2026-02-12

**Authors:** Nicolaj Kofod, Matthew E. Thornton, Abigail Richardson, Charles Smith, Sabina Gurung, Patrick Parkinson, Stephen Faulkner, Sam Hay, Louise S. Natrajan

**Affiliations:** ^1^ Department of Chemistry The University of Manchester Manchester UK; ^2^ Manchester Institute of Biotechnology The University of Manchester Manchester UK; ^3^ The Photon Science Institute The University of Manchester Manchester UK; ^4^ Department of Physics and Astronomy The University of Manchester Manchester UK; ^5^ Chemistry Research Laboratory Department of Chemistry University of Oxford Oxford UK

**Keywords:** lanthanide photophysics, lanthanide upconversion, molecular dynamics

## Abstract

Photon Upconversion in molecular hetero‐metallic lanthanide systems is challenged by the lack of chemical diversity displayed by the lanthanide ions. Here, we report the multi‐photon photophysical properties of a series of molecular hetero‐trimetallic lanthanide complexes **Yb_2_Ln** (Ln = Eu^3+^, Gd^3+^, Tb^3+^) assembled from kinetically inert building blocks providing site‐specific chemical control regarding introduction of differing lanthanide ions. The hetero‐trimetallic complex **Yb_2_Tb** shows efficient Yb_2_ **→** Tb photon upconversion via cooperative sensitization in both D_2_O and H_2_O. By contrast, **Yb_2_Eu** does not show Yb_2_ **→** Eu upconversion, while **Yb_2_Gd** has been used as a spectroscopic blank. We find that the Yb_2_ **→** Tb energy transfer appears to be independent of OH quenching from the solvent. Additionally, we report the intermetallic distances in the complex using density functional theory and molecular dynamics simulations. We find that the Yb_2_ **→** Tb cooperative sensitization upconversion energy transfer remains effective despite relatively long intermetallic distances between donor pairs (13.5–25 Å) and between the Yb donors and the Tb acceptor (11.5–13.5 Å).

## Graphical abstract

Lanthanides are at the forefront of photon upconversion in molecular systems; however, the chemical nature of the lanthanides makes site‐specific coordination chemistry difficult to achieve. Here, we employ kinetically stabile building blocks to achieve hetero‐trimetallic 4*f* complexes with complete site‐specific chemical control. We observe efficient Yb_2_ **→** Tb upconversion in aqueous solution despite relatively long intermetallic distances.

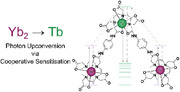

## 1 Introduction

**FIGURE 1:**
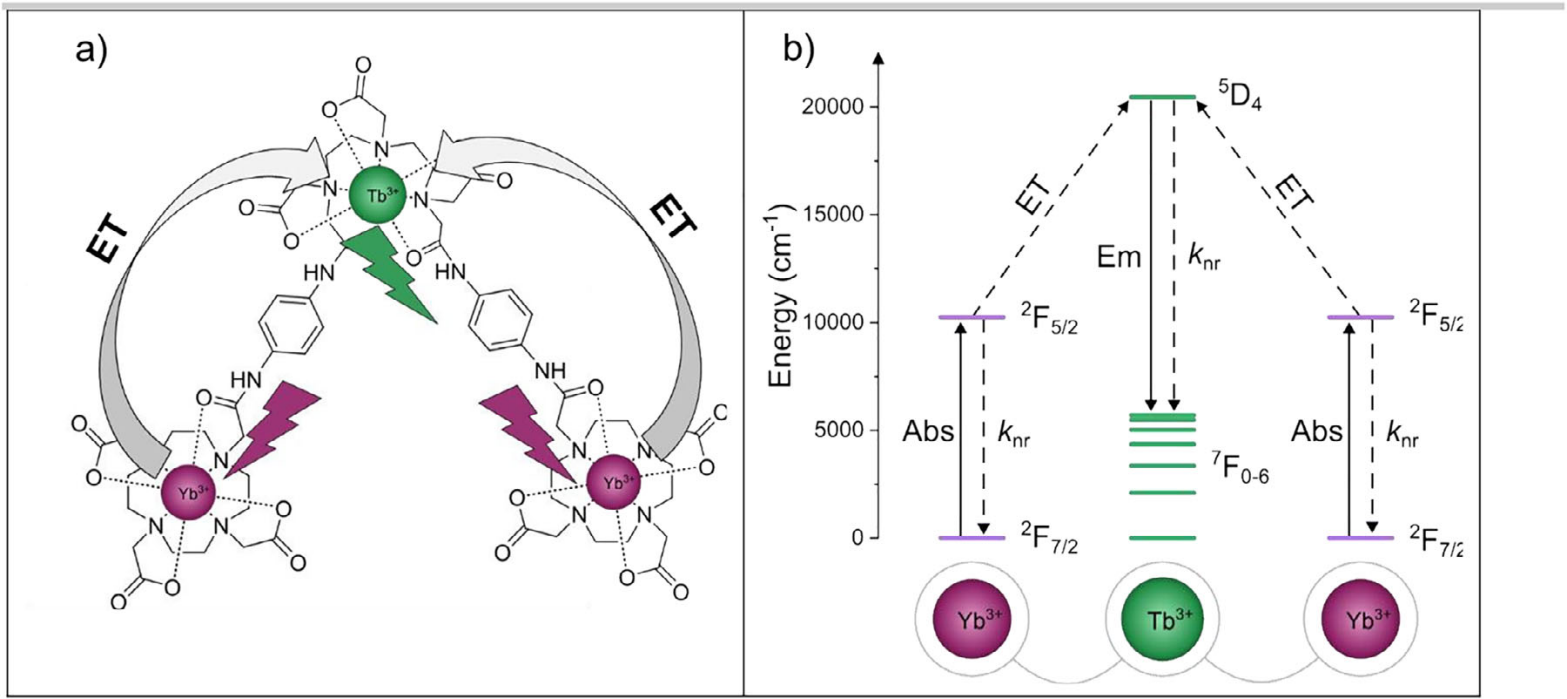
(a) Schematic of the molecular system and energy transfer pathway in **Yb_2_Tb**; (b) Schematic of the energy levels involved in Yb_2_ → Tb cooperative sensitization in **Yb_2_Tb**. Sequential excitation of two Yb^3+^ ions from ^2^F_7/2_ → ^2^F_5/2_ (10200 cm^−1^) can result in photon UC generating a single Tb^3+^ excited state via excitation into the ^7^F_6_ → ^5^D_4_ (20470 cm^−1^) transition. Solid lines represent radiative processes, absorption, and emission. Dashed lines represent non‐radiative processes, quenching (*k*_nr_) and energy transfer (ET). Energy levels taken from [43, 44].

Upconversion (UC) is an anti‐Stokes process where subsequent absorption of two or more photons of low energy, usually near infrared (NIR), results in the emission of one photon of higher energy [1, 2]. UC has gathered much interest for biological applications in particular, as background signals from autofluorescence and light scattering are greatly reduced, and the excitation is typically well within the transparent region of biological tissue (650–1300 nm) [3, 4, 5, 6, 7, 8, 9, 10, 11, 12, 13, 14, 15]. Trivalent lanthanide ions are particularly well suited for UC systems, due to their long‐excited state lifetimes (µs‐ms), narrow optical bands, and energetically well‐defined spin orbit coupled excited states, affording ‘ladder‐like’ energy levels for UC to operate [16, 17, 18]. The main challenges of using lanthanide ions are the low absorption coefficients of direct *f*‐*f* excitation, the susceptibility of quenching of the intermediate excited state, and emitting state, especially by solvent vibrations, and the lack of chemical control of coordination compounds [17, 19, 20, 21, 22, 23]. This has resulted in most examples of lanthanide UC being solid state or nanoparticle systems [24, 25]. Since the seminal paper by Piguet et al in 2011 [26], several authors have reported UC in discrete molecular lanthanide complexes [7, 14, 17, 18, 27, 28, 29, 30, 31, 32, 33, 34, 35, 36, 37, 38, 39]. These systems generally involve supramolecular self‐assembly, rendering the lanthanide ions kinetically labile–that is ligands where dissociation of the metal ion by competitive coordinating agents can occur–therefore reducing their potential use in biological applications [40]. Here, we report the first example of a hetero‐trimetallic kinetically inert molecular lanthanide system assembled from macrocyclic and acyclic polyaminocarboxylates [41, 42] (regularly employed in medical imaging and therapy), **Yb_2_Tb**, that displays green UC via cooperative sensitization in room temperature H_2_O, as illustrated in Figure 1.

## 2 Experimental Section

Full synthetic details have been previously reported by us and are detailed in the Supporting Information [45]. All chemicals and solvents were used as received. 1.5 mM of **Yb_2_Ln** (Ln = Eu^3+^, Gd^3+^, Tb^3+^) was dissolved in D_2_O (99.9% D Sigma Aldrich) and 3 mM of **Yb_2_Tb** was prepared in Milli‐Q H_2_O. All measurements were carried out in 10 optical path length mm quartz cuvettes from Starna Scientific. No variations in signal were detected over time.

Multi‐photon luminescence measurements we made by focusing the tuneable output of a Spectra‐Physics Mai Tai Ti:sapphire oscillator (100 fs, 80 MHz) on to the sample using an extra‐long working distance (ELWD), 40X air immersion objective (Nikon plan fluor ELWD: 2.80–3.60 mm, 0.6 NA). The incident laser power was varied by rotating an achromatic half waveplate before a linear polarizer and measured with a power meter. The luminescence was detected in epifluorescence mode via a long‐pass dichroic mirror with a cut‐on wavelength of 650 nm (Thor Labs, FEL0650). To reduce residual and scattered laser light, a 700 nm short‐pass filter was used (Thor Labs, FESH0700). Fluorescence was collected using a compact fiber optic coupled CCD spectrometer (Ocean Optics QE65000) and processed using SpectraSuite.

Time‐resolved measurements were carried out with a in a DCS‐120 Super MPC FLIM System from Becker and Hickl, where a Spectra‐Physics Insight X3 tuneable laser is frequency doubled in a second harmonic generator and then coupled to Nikon Eclipse Ti2‐U microscope via a confocal galvo‐mirror scan head. The excitation was set to 960 nm and a combination of long pass and band pass filters were used to isolate fluorescence in the range 400–800 nm.

Knife‐edge measurements were used to determine the spot size (beam radius) of the laser beam at the excitation wavelengths employed. In this method, a sharp knife edge was mounted on a translation stage with a resolution of 1 µm. The knife edge was positioned at the focal plane of the objective lens, and a power meter was placed to measure the transmitted laser power. Initially, the laser beam was completely blocked by the knife edge. The knife was then translated across the beam in 1 µm increments, resulting in a gradual increase in the detected power. The measurement continued until the beam was fully transmitted to the power meter. Using this procedure, the beam radius was determined to be 10.7 ± 0.8 µm at 850 nm, 11.1 ± 0.8 µm at 920 nm, 11.5 ± 0.9 µm at 960 nm and 11.8 ± 0.9 µm at 980 nm.

Density functional theory (DFT) calculations were performed on **Yb_2_Tb** with the PBE functional, Stuttgart large‐core effective‐core‐potentials for Tb^3+^ and Yb^3+^, cc‐VDZ basis set for all other atoms, CPCM implicit solvation model (water) and the Grimme D3 dispersion correction using Orca 6.0.1 [46, 47, 48, 49, 50, 51, 52, 53, 54, 55, 56, 57]. Geometry optimizations were performed with 1 water directly coordinated to the Tb^3+^ ion based on experimental results [45]. A non‐exhaustive conformational search was performed to account for the ligand symmetry surrounding the central Tb^3+^ ion with 3 major conformations (Figure S41) identified. These DFT models were used as input geometries for the subsequent molecular dynamics (MD) simulations.

MD calculations were performed using the CHARMM36 forcefield [58] and TIP3P water model [59] using GROMACS versions [60] 2023.3 and 2025.1. The topology and coordinate files for the ligand were generated using the CHARMM General Force Field (CGenFF) program. [61, 62, 63] Yb^3+^ and Tb^3+^ ions were added to the Gromos87 format molecular structure, and the TIP3P ion parameters [64, 65] for the trivalent ions Yb^3+^ and Tb^3+^ were added to the topology file. After solvation, geometry optimization, and equilibration, 1 µs MD simulations were performed with a constant temperature and pressure of 300 K and 1 bar, LINCS constraints [66], 1.0 nm short‐range electrostatic cut‐offs, particle mesh Ewald for long‐range electrostatics, and a time step of 2 fs. Intermetallic distances, water coordination, and dihedral angles were calculated using VMD 1.9.4 [67]. The number of coordinated waters was calculated with a cut‐off distance of 2.7 Å between the metal center and the solvent oxygen atoms.

## 3 Results and Discussions

**SCHEME 1:**
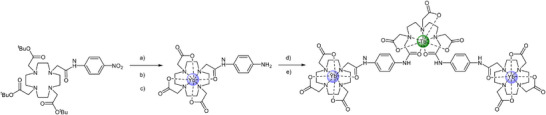
General synthetic procedure for Yb_2_Ln (**Yb_2_Tb** shown). Reagents and conditions: (a) NH_2_NH_2_.H_2_O, Pd/C, EtOH, 78°C; (b) CF_3_CO_2_H: CH_2_Cl_2_ 1:1 v:v; (c) Yb(OTf)_3_, MeOH, 40°C; (d) DTPA‐bis‐anhydride, K_2_CO_3_, DMF; (e) Tb(OTf)_3_, MeOH, 40°C.

The synthesis and one‐photon optical properties of **Yb_2_Ln** (Ln = Eu^3+^, Tb^3+^) have been reported by us previously and the Gd^3+^ derivative was prepared analogously using established procedures as illustrated in Scheme 1 [45, 66]. Use of the 2,2′,2′′,2′′′‐(1,4,7,10‐tetraazacyclododecane‐1,4,7,10‐tetrayl)tetraacetate (DOTA) and diethylenetriaminepentaacetate (DTPA) ligand motifs ensures kinetic stability and site‐specific chemical control of the lanthanide coordination [40, 45, 68, 69, 70]. In brief, alkylation of the well‐known *tert*‐butyl triester of cyclen with 2‐chloro‐*N*‐(4‐nitro‐phenyl)‐acetamide followed by reduction with hydrazine hydrate afforded the amine 10‐[1,4,7‐tris(*tert*‐butoxycarbonylmethyl)‐1,4,7,10‐tetraazacyclododecan‐1‐yl]‐*N*‐(4‐amino‐phenyl)‐acetamide) [71]. Removal of the *tert*‐butyl protecting groups with trifluoroacetic acid followed by complexation with a slight excess of Yb(OTf)_3_ resulted, after basic work up to remove any uncomplexed Yb^3+^, the Yb‐(DO3A)‐aminophenyl acetamide (**Yb**). Subsequent ring opening of DTPA‐anhydride with two equivalents of **Yb** and treatment with a slight excess of Ln(OTf)_3_ as above gave the target **Yb_2_Ln** complexes (Ln^3+^ = Eu, Tb, Gd) after workup and recrystallisation from methanol/diethyl ether [45]. Full synthetic and characterization data are provided in the Supporting Information; the complexes exhibited mass peaks commensurate with the predicted isotope patterns for **Yb_2_Ln** (Figures S1–S3) and paramagnetically shifted ^1^H NMR spectral shifts typical of Yb‐DO3A and Ln(DTPA) binding sites [45, 71].

**FIGURE 2:**
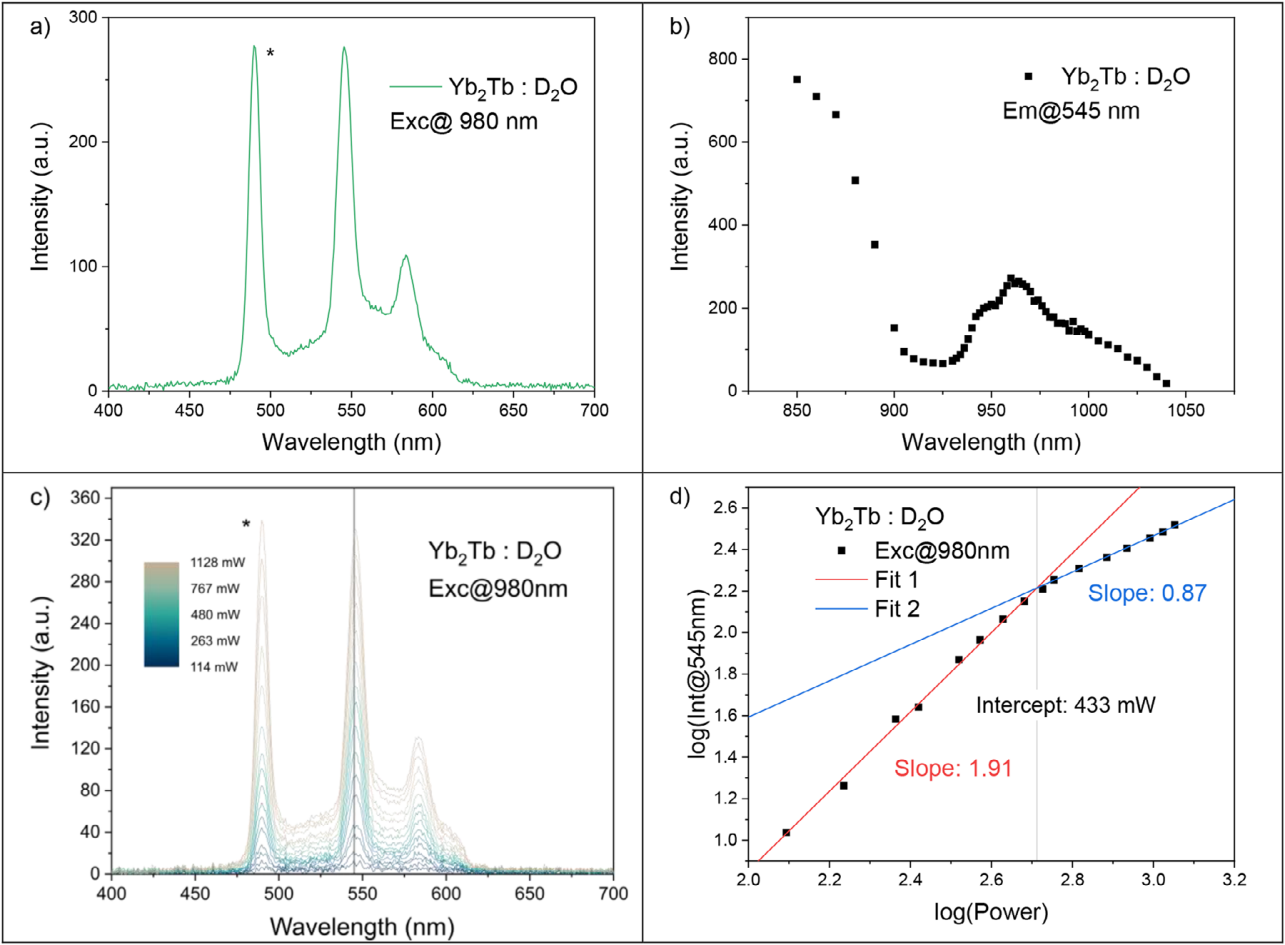
(a) Emission spectra of **Yb_2_Tb** in D_2_O excited through Yb^3+^ at 980 nm (laser beam radius = 11.8 ± 0.9 µm). Signal arising from the residual second harmonic of the laser is at 490 nm is marked by *. (b) Excitation spectra of **Yb_2_Tb** in D_2_O measured in the Tb^3+^ peak at 545 nm. (c) UC emission spectra of **Yb_2_Tb** in D_2_O excited at 980 nm used for power dependence determination from low‐power (blue) to high‐power (gray). The second harmonic of the excitation signal at 490 nm is denoted with *. The vertical line indicates wavelength (545 nm) used for power dependence determination. (d) Power dependence of UC emission at 545 nm for **Yb_2_Tb** in D_2_O, excited at 980 nm. The data has been fitted with two linear functions. The breakpoint was determined using a Chow‐test.

The Yb–Tb pair is an attractive candidate for cooperative sensitization from Yb_2_ **→** Tb as the Yb^3+^ excited ^2^F_5/2_ state (10,200 cm^−1^) is very near half of the Tb^3+^ excited ^5^D_4_ state (20,470 cm^−1^), see Figure 1 [41, 42]. We therefore explored the two‐photon photophysical properties of **Yb_2_Tb** in D_2_O to maximize the excited state lifetime of intermediate and emissive lanthanide states [20, 22, 72]. Excitation of **Yb_2_Tb** at 980 nm shows two clear luminescence signals in the 450–650 nm range – a broad band and two sharp emission peaks characteristic of Tb^3+ 5^D_4_ **→** ^7^F_5_ (545 nm) and ^5^D_4_ **→** ^7^F_4_ (585 nm) emission, see Figure 2a (and Figures S5 and S10). We tentatively attribute the broad emission feature to triplet emission, likely from the carbonyl groups of the ligand scaffold as the spectral range is in good agreement with literature [73], displays a room temperature time decay constant of 124 µs in deaerated H_2_O (Figures S16, S30, and S31) and does not correspond to the measured aryl ligand phosphorescence or fluorescence (Figures S42 and S34) [45]. This, formally forbidden triplet‐singlet transition is amplified by the heavy atom effect of the proximate lanthanide ions, allowing phosphorescence to be detectable at room temperature in non‐frozen solution [74]. Additional detectable Tb^3+^ emission peaks are expected at ^5^D_4_ **→** ^7^F_6_ (480 nm) and ^5^D_4_ **→** ^7^F_3_ (620 nm), but these are masked by the second harmonic of the laser and emission filters, respectively. The peak at 480 nm is clearly identified as residual excitation light from the second harmonic of the laser as the peak is dependent on excitation wavelength (Figure S9). The excitation spectra of the 545 nm peak, shows a sharp band between 900–1040 nm, on top of a broad band around 850 nm, see Figure 2b (and Figures S6, S7, and S10). The sharp band is characteristic of the Yb^3+ 2^F_7/2_ → ^2^F_5/2_ excitation [44, 75]. The broad feature in the emission spectra shows no Yb^3+^ based band in the excitation spectra (Figure S8). Likewise, excitation outside the Yb^3+^ band shows only weak Tb^3+^ emission peaks (Figure S11). Excitation at 850 nm again shows Tb^3+^ emission peaks, however, this is unlikely to be an UC process, *vide infra* (Figure S12). The emission spectra corresponding to Tb^3+^ is independent of excitation wavelength across the Yb^3+^ excitation band, Figure S9. After excitation at 960 nm, the ^5^D_4_ **→** ^7^F_6_ (480 nm) emission band of Tb^3+^ peak becomes visible, clearly showing the Yb_2_ **→** Tb UC. To further investigate the mechanism of UC in **Yb_2_Tb** we performed power dependence measurements at three excitation wavelengths. These are shown in Figure 2c,d and in Figures S18–S22. The power dependence is directly proportional to the number of photons involved in the process. The power dependence of the emission intensity at 545 nm after 980 nm excitation shows two clear regimes: at lower power the slope is ∼2 while at higher power the slope becomes ∼1. The break point between the two regimes was determined using a Chow Test [76]. The low‐power regime (<433 mW) corresponds to successive absorption of two photons. This, together with the possible energy levels involved, strongly indicates that the Yb_2_ **→** Tb UC proceeds via a cooperative sensitization mechanism. Here, the lack of a measurable rise time in the kinetic data corresponding to Yb^3+^ decay and population of the Tb^3+^ excited means we cannot rule out a contribution from an excited state absorption (ESA) mechanism. However, the fact that Tb^3+^ does not possess an intermediate energy level from which ESA can go through (Figure S4) and that we observe clear Yb^3+^ excitation bands the UC excitation spectra we can confidently conclude that CS is the primary ET pathway. By contrast, the high‐power regime (>433 mW) is less obvious. We interpret this as a photophysical steady‐state effect [77, 78, 79]. At higher power, one Yb^3+^ will continuously be in the excited state. Thus, the process becomes a *pseudo* one‐photon process. The emission band at 521 nm shows a power dependence of ∼1.4, (Figure S21). The same power dependence is observed after excitation outside the Yb^3+^ excitation band at 920 nm, indicating a non‐linear UC process through a virtual state. We note that the broad 521 nm phosphorescence does overlap with the Tb^3+^ emission peaks, but as the power dependence observed for this band itself is not present in the Tb^3+^ emission power dependence, we do not consider this to be problematic to the power law measurements. The Tb^3+^ emission observed after excitation at 850 nm shows a power dependence of ∼1. The exact nature of this process is not clear as its presence due to excitation by the second harmonic of the laser into the low energy tail of the ligand absorption band cannot be ruled out, but we can confidently conclude that it does not involve the Yb_2_ **→** Tb UC. Notably, this two‐regime power dependence behavior has been documented in several other UC systems, where there is a cross over point from 1 to 2 photons in the power dependence where excitation power dependencies vary between linear and quadratic [77, 78, 79].

**FIGURE 3:**
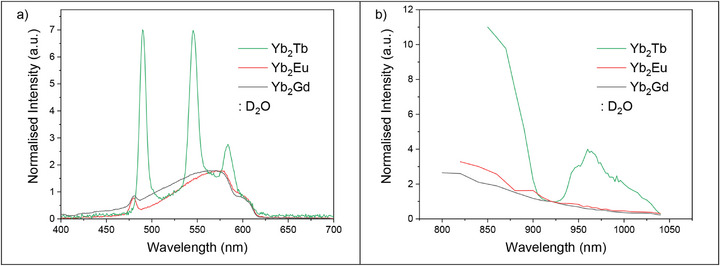
(a) Overlaid emission spectra of **Yb_2_Tb**, **Yb_2_Eu**, and **Yb_2_Gd** in D_2_O excited at the optimal energy to observe the most intense emission; 980 nm (**Yb_2_Tb**) (laser beam radius = 11.8 ± 0.9 µm) and 960 nm (**Yb_2_Eu** and **Yb_2_Gd**) (laser beam radius = 11.5 ± 0.9 µm). (b) Overlaid excitation spectra of **Yb_2_Tb**, **Yb_2_Eu** and **Yb_2_Gd** in D_2_O. Emission was measured at 545 nm (**Yb_2_Tb**) and 570 nm (**Yb_2_Eu** and **Yb_2_Gd**).

The Eu^3+^ ion possesses an excited state, ^5^D_2_ (21490 cm^−1^) near double that of the Yb^3+^ excited ^7^F_5/2_ state (10200 cm^−1^) and can thus potentially also exhibit Yb_2_ **→** Eu cooperative sensitization (see Figure S4) [75, 80], as recently observed in the solid state in an ion paired molecular system [81], co‐crystallized molecular assemblies [82] and in solution in a Eu‐Yb non‐nuclear cluster [83]. Thus, similar measurements were carried out on **Yb_2_Eu**, however, no discernible Eu^3+^ emission signal was observed upon Yb^3+^ excitation. Evidently, the energy match for Eu^3+^ is too poor to allow efficient Yb_2_ **→** Eu UC energy transfer in this system. Additionally, the complex **Yb_2_Gd** was prepared as a spectroscopically inactive reference sample. Both **Yb_2_Eu** and **Yb_2_Gd** show the same broad feature in the emission spectra as found for **Yb_2_Tb**, Figure 3a. No Yb^3+^ based bands are observed in the excitation spectra of **Yb_2_Eu** and **Yb_2_Gd**, Figure 3b [44, 75]. This is further indication that the broad emission signal arises from the ligand scaffold. The power dependence of the broad emission signal of **Yb_2_Eu** and **Yb_2_Gd** matches that of the **Yb_2_Tb** sample at lower powers (gradient = 1.3–1.5), see Figures S19–S25. At higher powers the power dependence of **Yb_2_Eu** and **Yb_2_Gd** increases to ∼2.5, which could possibly be due to higher‐lying excited states becoming involved/populated in the cooperative sensitization process due to the effect of the higher incident laser powers and/or heating effects of NIR excitation [84].

To verify that the assignment of the sharp emission bands in **Yb_2_Tb** are Tb^3+^ based, we performed time‐resolved emission measurements on all samples using a fluorescence and phosphorescence lifetime imaging set up (FLIM and PLIM) following excitation through the Yb^3+^ at 960 nm. The results are summarized in Table 1. All three samples show a tri‐exponential decay in the ns regime; plots given in the supplementary information, Figures S20–S26. A sample of pure solvent (D_2_O) shows the two shorter lifetimes arise from the laser signal/scatter, Figure S34. By tail fitting the emission decay traces (5–10 ns) we can extrapolate the excited state lifetime of the sample. All samples show a consistent excited state lifetime of 2.8–2.9 ns. While this may seem short for a T_1_ **→** S_0_ transition, it must be noted that the sample is measured in solution at room temperature, in the presence of three heavy atoms, and with no attempts to remove oxygen to reduce quenching of the phosphorescence. Indeed, in degassed H_2_O solution of **Yb_2_Eu**, the lifetime of this broad emission is observed in the PLIM window with a lifetime of 124 µs (Figures S16, S30, and S31), consistent with triplet emission. For **Yb_2_Tb** and **Yb_2_Eu**, a long‐lived emission signal is also observed with lifetimes of 2.5 and 1.9 ms, respectively, characteristic of Tb^3+^ and Eu^3+^ emission, S23 and S25. These are in good agreement with one‐photon measurements from our previous work [45]. The weak intensity of the Eu^3+^ signal explains why no Eu^3+^ emission peaks are observed in the steady state measurements. A very weak, long‐lived emission signal is observed for the **Yb_2_Gd** sample, Figures S32 and S33. However, due to the low intensity and the fact that the lifetime matches that of Tb^3+^, we attribute this to Tb^3+^ impurities in the sample and not a signal arising from the **Yb_2_Gd** sample. We note that while the long‐lived signal for **Yb_2_Tb** and, to a lesser extent, **Yb_2_Tb** are significantly more intense than the blank (Figures S34 and S35), the weak signal means the assignment remains somewhat tentative.

**TABLE 1:** Excited state lifetimes of **Yb_2_Ln** in D_2_O and H_2_O. All samples were excited at 960 nm. Short lifetimes were determined from tail‐fitting of the kinetic traces. Decay traces are given in Figures S24–S28 and Figures S36 and S37.

	*τ*_short_ (ns)	*τ*_long_ (ms)
**Yb_2_Tb** (D_2_O)	2.8 ± 0.2	2.5 ± 0.1
**Yb_2_Tb** (H_2_O)	2.1 ± 0.1	0.99 ± 0.001
**Yb_2_Eu** (D_2_O)	2.9 ± 0.1	1.9 ± 0.2
**Yb_2_Gd** (D_2_O)	2.8 ± 0.1	–*

* A small signal was detected in the ms range for **Yb_2_Gd**. We attribute this to small Tb^3+^ impurities in the sample, see Figure S32.

**FIGURE 4:**
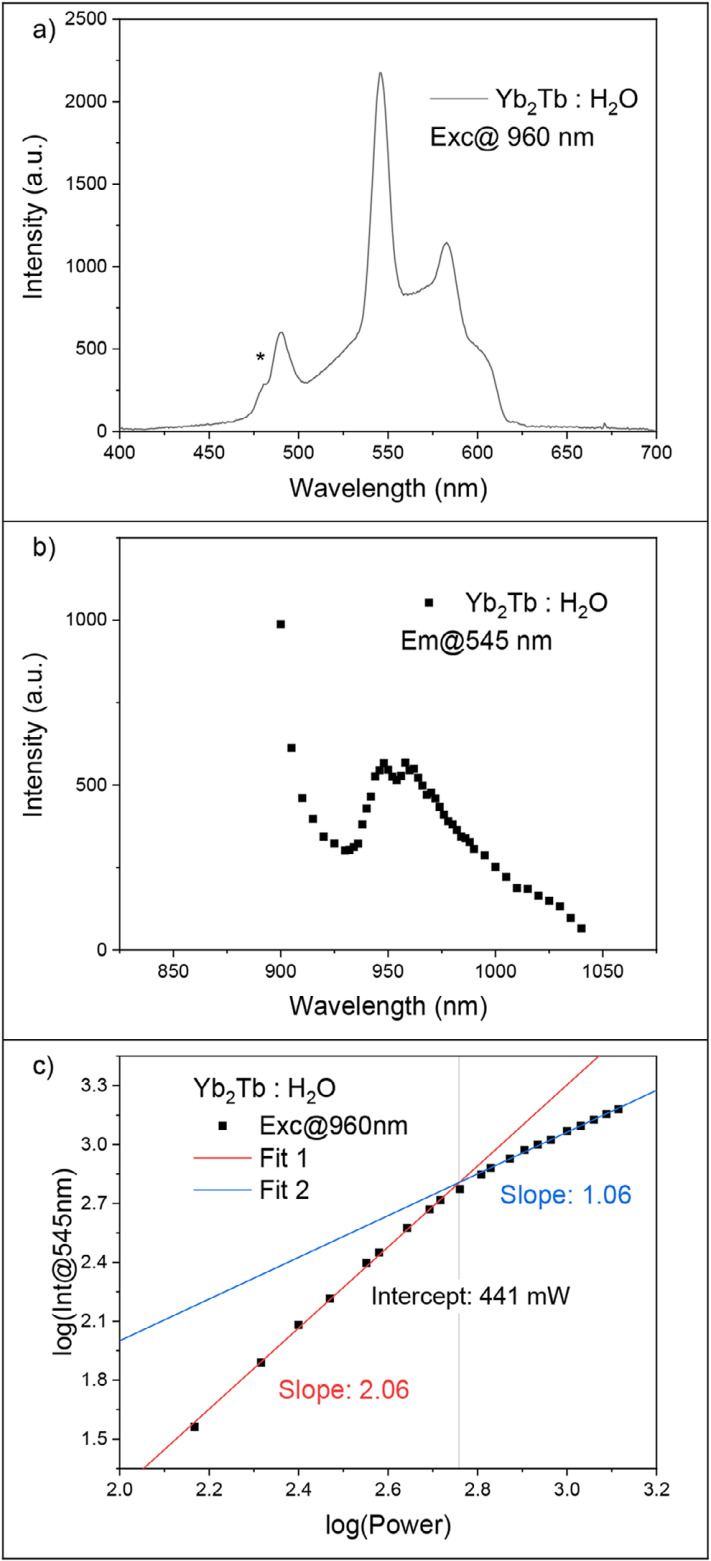
(a) Emission spectra of **Yb_2_Tb** in H_2_O excited at 960 nm. (b) Excitation spectra of **Yb_2_Tb** in H_2_O. Emission was measured at 545 nm. (c) Power dependence of **Yb_2_Tb** in H_2_O. Excitation was performed at 960 nm and emission was measured at 545 nm. Data was fitted with two linear functions.

The efficient UC signal of **Yb_2_Tb** in D_2_O led us to study the system in H_2_O. As the excited states of Yb^3+^ and Tb^3+^ are highly sensitive to quenching by OH oscillators, we expected to observe a much weaker, if any, UC emission signal [39]. To account for this, the concentration was increased from 1.5 mM for D_2_O samples to 3 mM for the H_2_O sample (see Figure S40 for the absorption spectra) [39]. Figure 4a shows the UC signal of **Yb_2_Tb** in H_2_O excited in the Yb^3+^ band. The emission and excitation spectra of **Yb_2_Tb** in H_2_O is identical to that observed in D_2_O, Figures 4a,b. This shows a surprising Yb_2_ **→** Tb UC efficiency, even in the presence of quenchers from the solvent.

The power dependence of **Yb_2_Tb** in H_2_O shows the exact same two regimes with a two‐photon process at low laser powers shifting to a “one‐photon” process at higher powers, Figure 4c. The break‐point was again determined using a Chow Test [72]. Surprisingly, the regime shift happens at the same power‐range, 433 and 441 mW for **Yb_2_Tb** in D_2_O and H_2_O, respectively. This indicates that the Yb_2_ **→** Tb energy transfer is largely unaffected by the shorter lifetime of the intermediate Yb^3+^ excited state. Based on our previous work, the Yb^3+^ excited state lifetime reduces fourfold from 8 to 2 µs from D_2_O to H_2_O [55]. From this, the Yb_2_ **→** Tb UC process is much faster than the excited state lifetime of Yb^3+^. We note that the emission intensity between samples is not directly comparable, but when corrected for concentration differences and under the same measurement conditions, the Yb_2_→Tb energy transfer appears to be independent of OH quenching since the ratio of the Tb^3+^ excited state lifetimes [45] between samples in H_2_O and D_2_O of 1.8 ± 0.1 is comparable to that of the Tb^3+^ emission intensities under multiphoton excitation of 1.7 ± 0.1. (See Figure S15 for graphical representation and further discussion). This is despite the relatively long Yb–Tb intermetallic distance in the system, estimated to ∼10–15 Å from DFT calculations (Table S7).

To better understand how the intermetallic distances and degree of direct coordination by H_2_O in **Yb_2_Tb** fluctuates in solution, we performed molecular dynamics (MD) simulations of the **Yb_2_Tb** in a H_2_O box at 300 K. 1 µs simulations were performed with 3 different starting geometries to account for the symmetry around the Tb‐DTPA binding pocket (see Figure S45). Directly coordinated H_2_O greatly affects the excited state lifetimes of lanthanide ions [19, 20, 21, 22], and thus, is expected to have a strong effect on the UC efficiency. From single‐photon experiments, the solvation (*q*) is estimated to be ∼0 for both Yb^3+^ ions and ∼1 for Tb^3+^ in **Yb_2_Tb** [45]. The MD simulations treated each metal as an ion (*i.e*. without explicit ligand‐metal bonding restrictions), allowing decomplexation to occur during the trajectory. The simulations show that both Yb^3+^ centers predominately have 0 waters coordinated, see Table 2. For the Tb^3+^ center, the *trans* conformation predominately has 1 water, while the two *cis* conformations show a mixture between predominately 1 and 2 waters with a minor contribution of 3 waters coordinated, see Tables 2 and S1–S3. We note that the quenching from direct coordination of water to the Tb^3+^ center does not have any significant effect on the Yb_2_ **→** Tb energy transfer efficiency. The *trans* conformation is the best match with the experimental results (q_Tb_ = 1), which could indicate that this is the predominant structure in solution [45]. However, we find it likely that all three conformations interchange in solution faster than the experimental timescale of luminescence and ^1^H NMR spectroscopy, which is not captured in our 1 µs MD simulations, see Figures S49 and S50 for conformation histograms [45, 85, 86, 87].

**TABLE 2:** Number of coordinated waters at the three Ln centres of **Yb_2_Tb** from MD simulations averaged over the three **Yb_2_Tb** conformations given as percentage of frames with specified number of waters directly coordinated (Ln‐O <2.7 Å) during a 1 µs MD simulation. Individual contributions are given in Tables S3–S5.

$n_{\left(H_{2}O\right)}$	Tb	Yb1	Yb2
0	0.24%	99.97%	99.97%
1	57.10%	0.03%	0.03%
2	39.81%	–	–
3	2.85%	–	–

**FIGURE 5:**
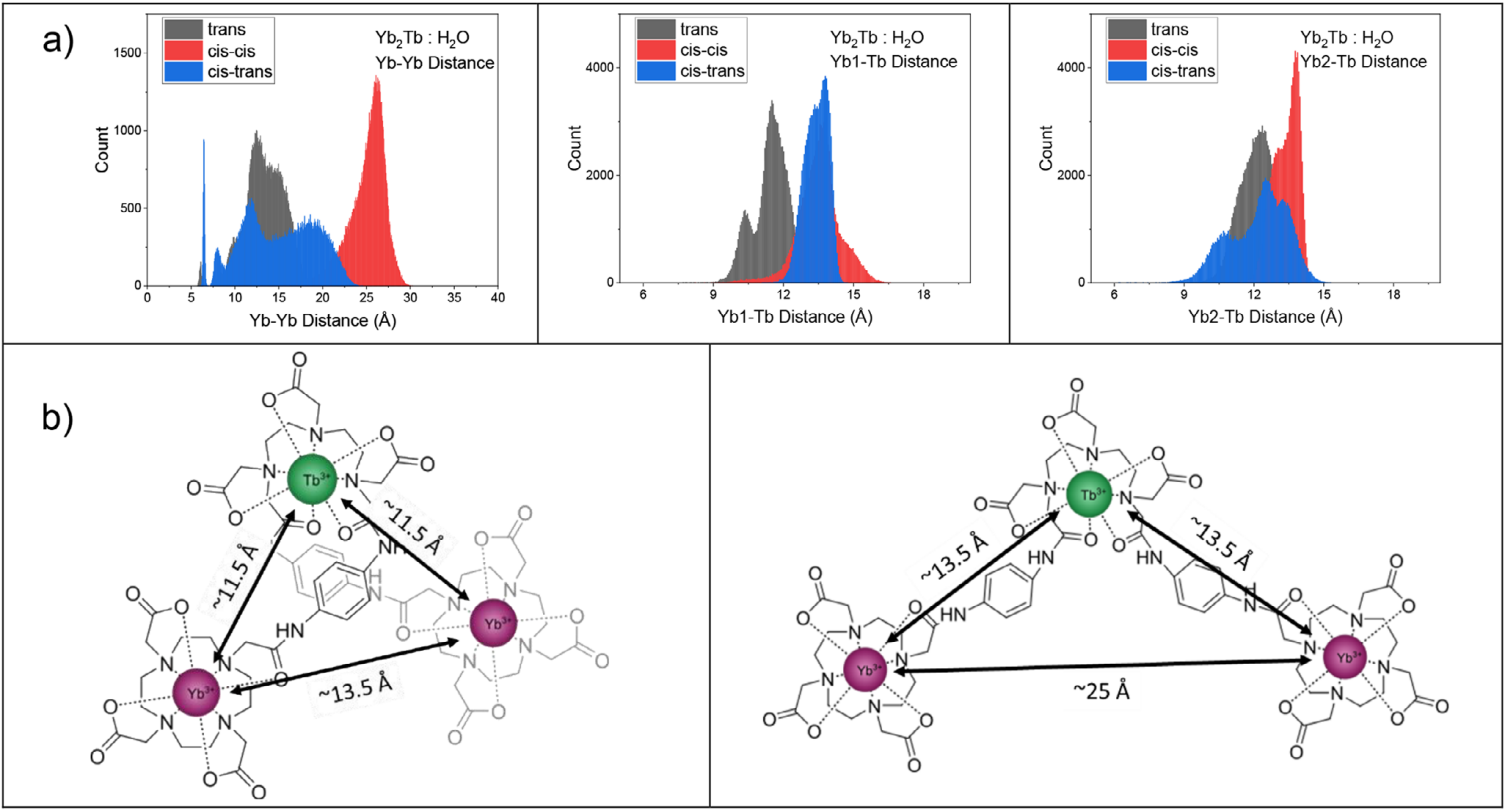
(a) Distribution of intermetallic distances observed during 1 µs MD simulations of the three conformations of **Yb_2_Tb**. Individual distributions as histograms are given in Figures S46 and S48; (b) Illustrations of the folded (left) and open (right) conformations.

The intermetallic distance is expected to affect the UC efficiency. The mechanism for the cooperative sensitization UC process is generally assumed to be a Förster Resonance Energy Transfer (FRET) type mechanism where shorter distances between donor and acceptor centers are crucial [1, 17, 36, 88]. The MD simulations show a distribution of intermetallic distances with two main conformations: an open and a folded conformation, shown in Figure 5. Snapshots of the molecular conformations along the MD trajectory are shown in Figure S51. The Yb–Yb distance is ∼13.5 Å for the folded conformation and ∼25 Å for the open conformation. The Yb–Tb distances are more constant, being slightly shorter, ∼11.5–12 Å, in the folded conformation to ∼13.5 Å in the open conformation. Population analysis of the MD trajectories (Table S9) show that the *trans* conformation predominantly (<90%) has both Yb–Tb distances <13 Å while the two *cis* conformations predominantly have longer Yb–Tb distances. Neither of the three conformations have a significant contribution (<1%) of conformations where both Yb‐Tb distances are <10 Å. These results show that even at long intermetallic distances of >10 Å, cooperative sensitization in lanthanide complexes remains effective and competitive with lanthanide excited state quenching. This is in line with donor‐acceptor energy transfer processes reported over distances >10 Å in the literature [89, 90].

## 4 Conclusions

Here, we present the two‐photon UC photophysical properties of kinetically inert hetero‐trimetallic **Yb_2_Ln** complexes (Ln = Eu^3+^, Gd^3+^, Tb^3+^) *via* Yb_2_ **→** Tb cooperative sensitization. The **Yb_2_Eu** and **Yb_2_Gd** complexes do not exhibit UC, whilst **Yb_2_Tb** shows efficient green UC in both D_2_O and H_2_O. We show that the Tb^3+^ emission is slower than quenching by OH oscillators and the Yb_2_ **→** Tb energy transfer efficiency appears to be independent of OH quenching. This is despite the relatively long intermetallic distances of ∼11.5‐13.5 Å between acceptor and donors and ∼13.5–25 Å between donor pairs observed during MD simulations. This is the first example of Yb_2_ **→** Tb UC in a molecular system made of kinetically stable building blocks with complete site‐specific chemical control. The use of multiphoton phosphorescence lifetime imaging (PLIM) to record the Tb^3+^ kinetic decay (lifetime) demonstrates that this such complexes are suitable candidates for UC optical imaging in aqueous solution paving the way to further development toward biomedical imaging applications.

## Conflicts of Interest

The authors declare no conflicts of interest.

## Supporting information




**Supporting File 1**: anie71363‐sup‐0001‐SuppMat.pdf.

## Data Availability

The data that support the findings of this study are available in the supplementary material of this article.
