# Supplementary material for: Yb2‐Tb Upconversion in a Hetero‐Trimetallic Molecular Lanthanide Complex
Source: Angew Chem Int Ed Engl. 2026 Feb 12;65(13):e19563. doi: 10.1002/anie.202519563 (PMC13007591; doi:10.1002/anie.202519563)
Supplement: Supplementary file 1 — Supporting File 1: anie71363‐sup‐0001‐SuppMat.pdf. [file ANIE-65-e19563-s001.pdf]

Supporting Information for: Yb_2-Tb Upconversion in a Hetero-Trimetallic Molecular Lanthanide Complex

Nicolaj Kofod*,[a] Matthew E. Thornton,[a] Abigail Richardson,[a,b] Charles Smith,[c] Sabina Gurung,[c] Patrick Parkinson,[c,d] Stephen Faulkner,[e] Sam Hay*,[a,b] Louise S. Natrajan*[a,c]

[a] Department of Chemistry, The University of Manchester, Oxford Road, Manchester, M13 9PL, UK.
[b] Manchester Institute of Biotechnology, The University of Manchester, 131 Princess Street, Manchester M1 7DN, UK.
[c] The Photon Science Institute, The University of Manchester, Oxford Road, Manchester, M13 9PL, UK.
[d] Department of Physics and Astronomy, The University of Manchester, Oxford Road, Manchester, M13 9PL, UK.
[e] Chemistry Research Laboratory, Department of Chemistry, University of Oxford, 12 Mansfield Road, Oxford, OX1 3TA, UK.

E-mail: nicolaj.kofod@manchester.ac.uk; sam.hay@manchester.ac.uk; louise.natrajan@manchester.uk

Synthetic procedures and characterisation data for ligands and complexes[1]

General considerations

1,4,7,10-tetraazacyclododecane (cyclen) was purchased from CheMatech and used without further purification. All other reagents and solvents were purchased from Sigma-Aldrich, Fluorochem Ltd. or Apollo Scientific Ltd. and used without further purification. Electrospray +/- (ES-MS) spectra were recorded on a Thermo Orbitrap Exactive Plus mass spectrometer. MALDI-TOF spectra were recorded on a Shimadzu Biotech Axima Confidence mass spectrometer. FT-IR spectra were recorded on a Bruker ALPHA I FT-IR spectrometer. Elemental analysis data were recorder using a Thermo Scientific FlashSmart Elemental Analyzer.

Multinuclear NMR spectra were recorded on a Bruker AVIII HD 500MHz spectrometer (BBFO inverse probe) in deuterated chloroform, deuterium oxide or deuterated methanol and analyzed using MestReNova 14.1.0. Chemical shifts in parts per million (ppm – δ) are reported relative to residual proton resonances and an internal tetramethylsilane reference. Splitting abbreviations: s: singlet, br. broad singlet, d: doublet, dd: doublet of doublets, t: triplet, dt: doublet of triplets, m: multiplet. Blank sections of spectra or those containing solvent resonances are omitted in certain spectra for clarity. Due to complex isomerism between the square and twisted square antiprismatic (SAP-TSAP) forms of multi-macrocyclic cyclen compounds, ^{1}H NMR assignments were often achieved *via* correlation with 2D COSY, HSQC and HMBC data where possible ^{1}H NMR data for compounds containing paramagnetic ions (Yb^{3+}, Tb^{3+}, Eu^{3+}) were processed using a line broadening/apodisation factor of 1.5 to 5 Hz and baseline corrected using a multipoint baseline correction with a Whittaker, cubic spline or segment algorithm. The chemical shift values of the ^{1}H resonances in all the lanthanide(III) complexes are reported without assignment due to the complex nature of the paramagnetic NMR assignment of polyaminocarboxylate lanthanide compounds.[1] The ^{1}H NMR spectroscopic data for **Yb_2Gd** and ^{13}C NMR spectroscopic data could not be collected for the same paramagnetic compounds due to rapid relaxation and extensive line broadening. Deaerated solutions for spectroscopic analysis were prepared by slow bubbling of the solution of the complex in a 1cm path length quartz cuvette fitted with a rubber septum with dry argon gas for 3 hours on a Schlenk line.

General method for preparation of complexes

Complexation of Ln^{3+} into deprotected DO3A ligands followed a general procedure analogous to literature previously reported. Complexation of Ln^{3+} into deprotected DTPA ligands followed the same general procedure with **bis-Yb-(DO3A)-aminophenyl acetamide DTPA, Yb_2DTPA,** as the starting material.

DO3A-(triacetic acid)-aminophenyl acetamide was dissolved in methanol and stirred. $Ln(OTf)_3$ dissolved in methanol was slowly added dropwise and the reaction mixture warmed gently to 40°C for 72 h. All volatiles were removed under reduced pressure and the residue dissolved in minimal deionized water. The pH was adjusted to ~10 with 0.1 M NaOH to precipitate any uncomplexed Ln^{3+} as the hydroxide, and the solution filtered through a pad of celite. The pH was then readjusted to ~6 with 0.1 M HCl and all solvents removed under reduced pressure. Minimal ethanol was added and the solution and sonicated to dissolve, then left to stand at 4°C for several hours before filtration and then being washed with ethanol. All volatiles were then removed, and the product was re-dissolved in minimal methanol. The product was slowly precipitated by layering the concentrated methanol solution with diethyl ether and allowed to diffuse for 48 hours at room temperature. The solid was filtered, washed with diethyl ether and dried under vacuum to isolate the complex as a white solid.

Preparation of 1,4,7-tris(tert-butoxycarbonylmethyl)-1,4,7,10-tetraazacyclododecane

1,4,7,10-Tetraazacyclododecane (17.4 g, 0.10 mol) and sodium hydrogen carbonate (28 g, 0.33 mol) were stirred at 0°C in acetonitrile. *Tert*-butyl bromoacetate (45 mL, 0.30 mol) dissolved in acetonitrile was added dropwise over 4 hours and the mixture was stirred at room temperature for 48 hours. The resulting inorganic salts that precipitated were filtered off and washed with acetonitrile. The filtrate was evaporated under reduced pressure (< 40°C) to leave a white solid. Warm toluene (< 50°C) was added to the crude product mixture and stirred, then left to stand at room temperature overnight. The white powder precipitate was filtered and washed with toluene then diethyl ether and dried under vacuum to isolate the product as a white powder in 44% yield (22.9 g).

^{1}H NMR (500 MHz, CDC_{l3}) δ (ppm): 1.45 (s, 9H, Bu **H**), 1.46 (s, 18H, ^{t}Bu **H**), 2.87 (br. s, 4H N-C**H**2), 2.92 (m, 8H, N-C**H**2), 3.10 (m, 4H, NH-C**H**2), 3.29 (s, 2H, CO-C**H**2), 3.37 (s, 4H, CO-C**H**2), 10.03 (br. s, 1H, N**H**). 13C NMR (126 MHz, CDCl3) δ (ppm): 28.33, 28.37 (^{t}Bu **C**H3), 47.67, 49.33, 51.35, 51.49 (NH-**C**H2), 58.37 (CO-**C**H2), 81.83, 81.99 (^{t}Bu **C**), 169.76,170.65 (**C**O). EI-MS: *m/z* = 515 [M]+ (100%), 537 $[M+Na]^+$ (20 %). IR (FT-IR) ν (cm^{-1}): 2998, 2979, 2941, 2852 (sp^3 C-H stretch); 2732; 1718 (C=O stretch); 1450, 1368 (C-H bend); 1254 (acyl C-O stretch); 1147 (alkoxy C-O stretch). CHN elemental analysis $C_{26}H_{50}N_4O_6\cdot HBr\cdot H_2O$ (%): Expected C 50.89, H 8.71, N 9.13; found C 50.94, H 8.71, N 9.45.

2-Chloro-N-(4-nitro-phenyl)-acetamide

4-Nitroaniline (10 g, 0.072 mol) was dissolved in acetonitrile. Sodium hydrogen carbonate was added (10.1 g, 0.12 mol) and the mixture stirred at 0°C. Chloroacetyl chloride (6 mL, 0.075 mol) diluted in acetonitrile was added dropwise to the solution over 2 hours and the mixture allowed to stir overnight at room temperature. The sodium hydrogen carbonate was filtered off and solvent removed under reduced pressure. The yellow solid was dissolved in hot toluene and allowed to crystallize in the refrigerator

overnight (~ 4°C). The solid was filtered and washed with cold toluene, to yield a yellow powder in 94% yield, (14.6 g).

^{1}H NMR (500 MHz, $CDCl_3$) δ: 4.24 (s, 2H, C**H**$_2$), 7.77 (dt, 2H, $^{3}J_{H-H}$ = 9.1 Hz, $^{4}J_{H-H}$ = 3 Hz, NH-Ar **H**), 8.26 (dt, 2H, $^{3}J_{H-H}$ = 9.1 Hz, $^{4}J_{H-H}$ = 3 Hz, NO_2-Ar **H**), 8.49 (br. 1H, N**H**). ^{13}C NMR (126 MHz, $CDCl_3$) δ 43.21 (**C**H$_2$), 119.90 (NH-Ar **C**H), 125.55 (NO_2-Ar **C**H), 142.68 (NH-Ar **C**), 144.74 (NO2- Ar **C**), 164.55 (**C**O). EI-MS: *m/z* = 213 [M-H]- (100%). IR (FT-IR) ν (cm-1): 3315 (N-H stretch); 3274, 3226, 3163, 3107 (sp2 C-H stretch); 2941 (sp3 C-H stretch); 1685 (C=O stretch); 1623, 1566 (C=C stretch); 1502 (N=O asymm. stretch); 1405 (sp^3 C-H bend); 1336 (N=O symm. stretch); 850, 748 (sp^2 C-H bend). CHN elemental analysis $C_8H_7N_2O_3Cl$ (%): Expected C 44.77, H 3.29, N 13.05; found C 44.09, H 3.19, N 13.02.

10-[1,4,7-tris(tert-butoxycarbonylmethyl)-1,4,7,10-tetraazacyclododecan-1-yl]-N-(4-nitro-phenyl)-acetamide

DO3A-(tris-*tert*-butyl ester) (7 g, 13.6 mmol) was dissolved in acetonitrile. Sodium carbonate (3.17 g, 29.9 mmol) was added, and the solution stirred at room temperature for 1 hour. 2-chloro-N-(4-nitro-phenyl)-acetamide (**2**) (3.22 g, 15 mmol) in minimal acetonitrile was added dropwise over 4 hours. The pale yellow mixture was stirred at room temperature for 24 hours then heated to reflux temperature for 24 hours. The sodium carbonate was filtered off and the solvent removed under reduced pressure. The yellow residue was dissolved in hot toluene and left in the refrigerator overnight (~ 4°C). The crude product precipitate was filtered off and the filtrate evaporated under reduced pressure to yield another crude yellow oil product. Both residues were purified by column chromatography using a DCM:MeOH gradient. Initial treatment with pure DCM to eluted a pale yellow band, followed by two further colored fractions upon increasing the polarity with MeOH (< 5%). The pure fractions were collected, and the solvent removed under reduced pressure to yield a pale yellow powder in 75% yield (7.10 g).

^{1}H NMR (500 MHz, CDCl3) δ 1.48 (s, 27H, ^{t}Bu **H**), 1.90 – 3.63 (m, 16H, N-C**H**$_2$), 3.78 (s, 8H, CO-C**H**$_2$), 8.07 (dt, 2H, $^{3}J_{H-H}$ = 9.4 Hz, $^{4}J_{H-H}$ = 2.4 Hz, NH-Ar **H**), 8.12 (qt, 2H, $^{3}J_{H-H}$ = 9.4 Hz, $^{4}J_{H-H}$ = 2.4 Hz, NO_2-Ar **H**), 11.37 (br. s, 1H, N**H**). ^{13}C NMR (126 MHz, $CDCl_3$) δ 28.01, 28.04 (^{t}Bu **C**H$_3$), 48.48, 52.66 (N-**C**H$_2$), 55.70, 55.82, 57.18 (CO-**C**H$_2$), 82.25, 82.37 (^{t}Bu **C**), 119.79 (NH-Ar **C**H), 124.41 (NO_2-Ar **C**H), 142.81 (NH-Ar **C**), 145.51 (NO_2-Ar **C**), 172.46 (**C**ONH), 172.61 (**C**OO). EI-MS: *m/z* = 715 $[M+Na]^+$ (100%). IR (FT-IR) ν (cm-1): 3315 (N-H stretch); 3182, 3137 (sp2 C-H stretch); 2976, 2753 (sp3 C-H stretch); 1725 (C=O); 1697 (C=O stretch); 1595, 1560 (C=C stretch); 1508 (N=O asymm. stretch); 1452 (sp3 C-H bend); 1327 (N=O symm. stretch); 1227 (acyl C-O stretch); 1158 (alkoxy C-O stretch); 1105 (C-O stretch); 857, 734 (sp^2 C-H bend). CHN elemental analysis $C_{34}H_{56}N_6O_9{\cdot}0.75NaCl$ (%): Expected C 55.43, H 7.66, N 11.41; found C 55.75, H 7.29, N 9.05.

10-[1,4,7-tris(tert-butoxycarbonylmethyl)-1,4,7,10-tetraazacyclododecan-1-yl]-N-(4-amino-phenyl)-acetamide

DO3A-(tris-*tert*-butyl ester)-nitrophenyl acetamide (3.53 g, 5.09 mmol) was dissolved in ethanol and warmed to ~78°C. 50-60% hydrazine hydrate solution (14.1 mL) and Pd/C catalyst (wetted with ethanol) were added to the mixture, and the reaction stirred under reflux overnight. The dark solution was filtered into celite, and the filtrate evaporated under reduced pressure. The resulting yellow liquid was dissolved

in chloroform and extracted with distilled water to remove the hydrazine. The organic portion was dried over anhydrous MgSO4 and filtered. The filtrate was evaporated under reduced pressure to yield a crude yellow oil, which was purified *via* recrystallization. The crude product was dissolved in minimal DCM and hexane added dropwise until the solution was cloudy. The mixture was warmed to dissolve the product then placed in the freezer (-18°C) overnight to crystallise and yield an off-white precipitate. The solution was filtered, and the precipitate dried under reduced pressure to yield a beige powder in 48% yield (1.64 g).

^{1}H NMR (500 MHz, $CDCl_3$) δ 1.41 (br 18H, ^{t}Bu **H**), 1.45 (s, 9H, ^{t}Bu **H**), 1.90 – 3.63 (m, N-C**H**$_2$) 2.62 (br. s, 4H, N-C**H**$_2$), 2.77 (br. s, 4H, N-C**H**$_2$), 2.89 (dd, $^3J_{H\text{-}H}$ = 14.4 Hz, $^4J_{H\text{-}H}$ = 5.9 Hz, 4H, N-C**H**$_2$), 2.91 (dd, $^3J_{H\text{-}H}$ = 14.4 Hz, $^4J_{H\text{-}H}$ = 5.7 Hz 4H, N-C**H**$_2$), 3.15 (s, 4H, CO-C**H**$_2$), 3.29 (s, 2H, CO-C**H**$_2$), 3.50 (br. 2H, N**H**$_2$), 3.63 (s, 2H, CONH-C**H**$_2$), 6.53 (dt, 2H, $^3J_{H\text{-}H}$ = 8.7 Hz, $^4J_{H\text{-}H}$ = 5.1 Hz, NH$_2$-Ar **H**), 7.68 (dt, 2H, $^3J_{H\text{-}H}$ = 8.8 Hz, $^4J_{H\text{-}H}$ = 5.1 Hz NH-Ar **H**), 10.19 (br. 1H, N**H**). ^{13C} NMR (126 MHz, CDCl3) δ 28.05, 28.10 (^{t}Bu **C**H3), 51.91, 52.21 (CO-**C**H$_2$), 52.65 (CONH-**C**H$_2$), 54.85, 55.80, 55.86, 56.83, 56.91, 59.37 (N-**C**H$_2$), 81.08, 81.97, 82.12 (^{t}Bu **C**), 115.08 (NH2-Ar **C**H), 121.78 (NH-Ar **C**H), 131.28 (NH-Ar **C**), 142.10 (NH2-Ar **C**), 170.19 (**C**ONH), 170.78, 172.37 (**C**OO). EI-MS: *m/z* = 685 $[M+Na]^+$ (100%). IR (FT-IR) ν (cm-1): 3182, 3122 (sp^2 C-H stretch); 2976, 2821 (sp^3 C-H stretch); 1725 (C=O stretch); 1664 (C=O stretch); 1546, 1514 (C=C stretch); 1452, 1367 (sp^3 C-H bend); 1224 (acyl C-O stretch); 1156 (alkoxy C-O stretch); 1103 (C-O stretch); 838, 755 (sp^2 C-H bend). CHN elemental analysis $C_{34}H_{58}N_6O_7$·NaCl·$_2$H2O·0.5CH_2Cl_2 (%): Expected C 51.81, H 7.94, N 10.51; found C 51.85, H 7.75, N 10.94.

2,2',2''-(10-(2-((4-aminophenyl)amino)-2-oxoethyl)-1,4,7,10-tetraazacyclododecane-1,4,7-triyl)triacetic acid

DO3A-(tris-*tert*-butyl ester)-aminophenyl acetamide (1.57 g, 2.37 mmol) was dissolved in dichloromethane and trifluoroacetic acid (1:1 ratio) was added dropwise to the stirring solution. The brown mixture was allowed to stir at room temperature for 24 hours. All volatiles were then removed under reduced pressure and the residue washed repeatedly with dichloromethane and methanol. The product was slowly precipitated by layering the concentrated methanol solution with diethyl ether and allowed to diffuse for 48 hours at room temperature. The hygroscopic product was filtered, washed with diethyl ether and dried under vacuum to afford a white solid in 74% yield (0.87 g).

^{1}H NMR (500 MHz, D2O) δ 3.13 (m, 12H, N-C**H**$_2$), 3.46 (s, 2H, CONH-C**H**$_2$), 3.51 (m, 4H, N-C**H**$_2$), 3.65 (s, 2H, CO-C**H**$_2$), 3.83 (s, 2H, CO-C**H**2), 3.84 (s, 2H, CO-C**H**2), 7.17 (d, 2H, 3*JH-H* = 8.8 Hz, NH2-Ar **H**), 7.49 (d, 2H, $^3J_{H\text{-}H}$ = 8.7 Hz, NH-Ar **H**). ^{13}C NMR (126 MHz, D2O) δ: 48.09, 48.25, 50.92, 51.63 (N-**C**H2), 55.00, 55.91, 56.40 (CO-**C**H$_2$), 115.15 (NH2-Ar **C**), 117.47 (NH-Ar **C**), 120.95 (NH2-Ar **C**H), 122.76 (NH-Ar **C**H), 169.80, 170.63 (**C**OO), 176.92 (**C**ONH). MALDI-TOF MS (alpha/MeOH): *m/z* = 517 $[M+Na]^+$ (100%), 533 $[M+K]^+$ (97 %), 495 $[M+H]^+$ (95 %). IR (FT-IR) ν (cm-1): 3095 (sp^2 C-H stretch); 2961, 2847 (sp^3 C-H stretch); 1676 (C=O stretch); 1513 (C=C stretch); 1458, 1425 (O-H bend); 1198, 1126 (C-O stretch); 830, 799 720 (sp^2 C-H bend). CHN elemental analysis $C_{22}H_{34}N_6O_7$·2TFA·3H_2O (%): Expected C 40.21, H 5.45, N 10.82; found C 39.95, H 4.90, N 11.03.

Synthesis of Yb-(DO3A)-aminophenyl acetamide, Yb

DO3A-(tri-acetate)-aminophenyl acetamide (35.4 mg, 0.072 mmol) was reacted with $Yb(OTf)_3$ (49.6 mg, 0.082 mmol)) following the general method for preparation of complexes outlined above to result in an isolated yield of 39 mg of **Yb** as an off white powder.

^{1}H NMR (500 MHz, D2O) δ -75.18, -74.34, -71.31, -56.82, -54.53, -43.02, -40.07, -28.76, -26.27, -24.39, -20.47, 0.33, 2.22, 8.67, 9.10, 14.58, 15.11, 15.96, 18.11, 19.42, 24.01, 25.80, 31.55, 117.18, 122.05, 128.38. MALDI-TOF MS (alpha/MeOH): *m/z* = 688 $[M+Na]^+$ (100%), 710 [M+2Na-H] (51%), 704 $[M+K]^+$ (35%), 1373 $[2M+K]^+$ (8%), 1351 $[2M+Na]^+$ (4%). IR (FT-IR) ν (cm-1): 3349 (N-H stretch); 2971, 2914, 2867 (sp3 C-H stretch); 1608 (C=O stretch); 1516 (C=C stretch); 1384 (C-H bend); 1247, 1160 (C-O stretch); 1082, 1029 (C-N stretch); 834, 721 637 (sp^2 C-H bend).

Synthesis of {Yb(DO3A)}₂-DTPA, Yb₂DTPA

Under argon, complex **Yb** (314 mg, 0.47 mmol) was dissolved in dry dimethylformamide (DMF) and potassium carbonate (4 eq.) was added. Diethylenetriamine pentaacetic acid (DTPA) anhydride (67.5 mg, 0.19 mmol) was dissolved in anhydrous DMF and added to the stirring reaction. The reaction mixture was then heated to 55 °C for 14 days under argon. The solution was opened to air, cooled, and filtered. The filtrate was reduced *in vacuo* and the residue re-dissolved in methanol and then precipitated with diethyl and placed in a freezer (-18 °C) overnight. The beige solid was decanted, washed with diethyl ether, and dried thoroughly under vacuum. The desired complex was isolated as a hygroscopic beige solid (91 mg).

^{1}H NMR (500 MHz, D2O) δ -74.32, -73.27, -72.61, -69.75, -57.49, -56.44, -43.46, -43.19, -41.09, -40.37, -26.48, -25.97, -24.93, -24.52, -21.69, -15.93, 2.84, 3.00, 3.25, 3.30, 3.34, 3.54, 3.72, 8.45, 9.04, 9.47, 10.30, 10.67, 11.05, 11.44, 11.73, 12.10, 12.95, 13.38, 13.76, 14.16, 14.79, 16.00, 16.33, 17.36, 17.73, 18.11, 19.12, 23.24, 24.19, 25.07, 26.78, 33.23, 113.88, 114.67, 115.72, 119.71, 128.43. MALDI-TOF MS (alpha/MeOH): *m/z* = 1724 $[M+K]^+$ (100%), 1762 $[M+2K-H]^+$ (44%), 1746 $[M+IPA+H]^+$ (36%), 1708 $[M+Na]^+$ (26%), 1686 $[M+H]^+$ (16%). ESI$^+$ accurate mass (MeOH:H_2O), *m/z* = 866.2103 $[M+2Na]^{2+}$ (100%), expected: 866.2125. IR (FT-IR) ν (cm-1): 3293 (N-H stretch); 2949, 2839 (sp3 C-H stretch); 1734 (C=O stretch); 1583 (C=C stretch); 1400, 1321 (C-H bend); 1254 (C-O stretch), 1160, 1031 (N-H bend); 840, 717, 638 (sp2 C-H bend).

Preparation of {Yb(DO3A)₂-{Tb(DTPA)}, Yb₂Tb

Yb₂DTPA (74.2 mg, 0.044 mmol) was reacted with $Tb(OTf)_3$ (31.7 mg, 0.052 mmol) following the general method for preparation of complexes outlined above to afford 43 mg of **Yb₂Tb**.

^{1}H NMR (500 MHz, MeOD) δ -98.41, -87.54, -86.12, -85.42, -81.11, -68.47, -64.43, -63.88, -51.79, -48.85, -45.64, -43.33, -30.18, -28.66, -24.68, -16.70, -0.97, 0.09, 0.89, 1.16, 1.17, 1.19, 1.28, 1.38, 1.51, 10.13, 11.24, 14.85, 16.53, 18.95, 19.77, 21.23, 23.40, 25.90, 28.37, 31.16, 38.22, 125.80, 129.18, 134.34, 143.32. ESI-MS (MeOH:H_2O): *m/z* = 1882 $[M+K]^+$ (100%), 1844 $[M+H]^+$ (78%), 1866 $[M+Na]^+$ (67%), 952 $[M+Na+K]^{2+}$, 944 $[M+2Na]^{2+}$. ESI$^+$ accurate mass (MeOH:H_2O), *m/z* = 951.6577 $[M+Na+K]^{2+}$ (100%), expected: 951.6495, 943.6705 (45%), $[M+2Na]^{2+}$ expected 943.6626. IR (FT-IR) ν (cm-1): 3376 (N-H

stretch); 2977, 2872 (sp^3 C-H stretch); 1599, 1515 (C=C stretch); 1340, 1321 (C-N stretch); 1086, 1031 (C-O stretch); 932, 844, 718, 639 (sp^2 C-H bend).

Preparation of {Yb(DO3A)}$_2$-{Eu(DTPA)}, Yb$_2$Eu

Yb$_2$DTPA (42.8 mg, 0.025 mmol) was reacted with Eu(OTf)$_3$ (16.8 mg, 0.028 mmol) to afford 22 mg of **Yb$_2$Eu**.

^{1}H NMR (500 MHz, MeOD) δ -97.44, -85.57, -83.41, -66.71, -51.16, -49.70, -46.35, -42.87, -17.21, -16.70, -16.01, -13.70, -12.13, -9.70, -5.41, -3.40, -2.62, -0.72, 7.59, 8.58, 10.17, 13.08, 14.76, 17.17, 18.74, 19.74, 23.27, 25.56, 28.20, 30.01, 33.03, 36.09, 151.16.
MALDI-TOF: *m/z* = 1858 [M+Na]$^+$ (100%), 1875 [M+K]$^+$ (77%), 1837 [M+H]$^+$ (75%), 1896 [M+Na+K-H]$^+$ (74%), 1880 [M+2Na-H]+ (58%). IR (FT-IR) ν (cm-1): 32378 (O-H stretch); 2976, 2922 (sp^3 C-H stretch); 1593 (C=O stretch); 1515 (C=C stretch); 1401, 1323, 1253 (C-N stretch); 1164, 1088, 1030 (C-O stretch); 931, 718, 638 (sp^2 C-H bend).

Preparation of {Yb(DO3A)}$_2$-{Gd(DTPA)}, Yb$_2$Gd

Yb$_2$DTPA (8.61 mg, 0.00075 mmol) was reacted with Gd(OTf)$_3$ (4.99 mg, 0.00825 mmol) to afford 7.8 mg of **Yb$_2$Gd**.
ESI-MS (MeOH:H_2O), *m/z* = 943 [M+2Na]$^{2+}$ (30%), 1869 [M+Li]$^+$ (5%).

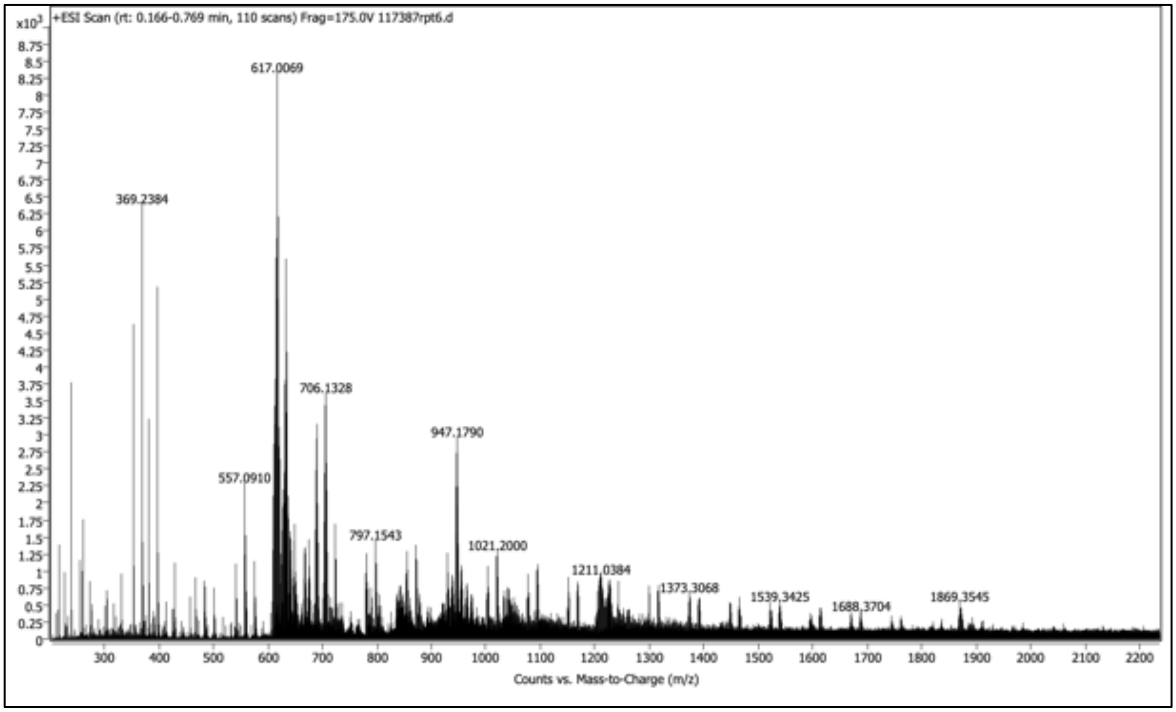

Figure S1. Electrospray mass spectrum of Yb$_2$Gd recorded in MeOH:H_2O in positive mode.

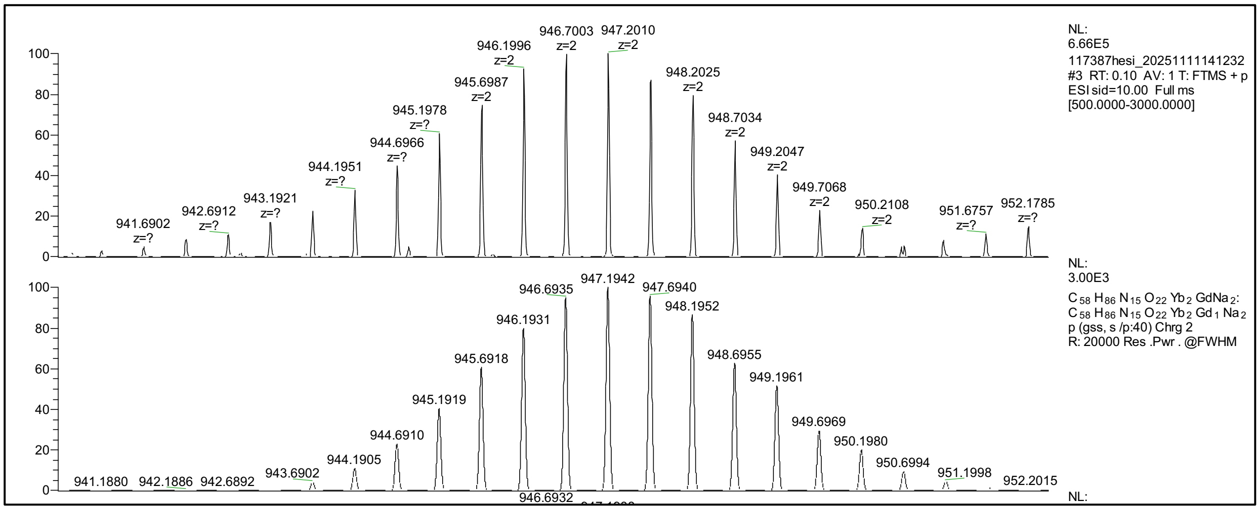

Figure S2. **Electrospray mass spectrum of Yb$_2$Gd recorded in MeOH:H$_2$O in positive mode showing, above: the experimental spectrum of the [M+2Na]$^{2+}$ peak at *m/z* 946 amu, and bottom: calculated isotope pattern.**

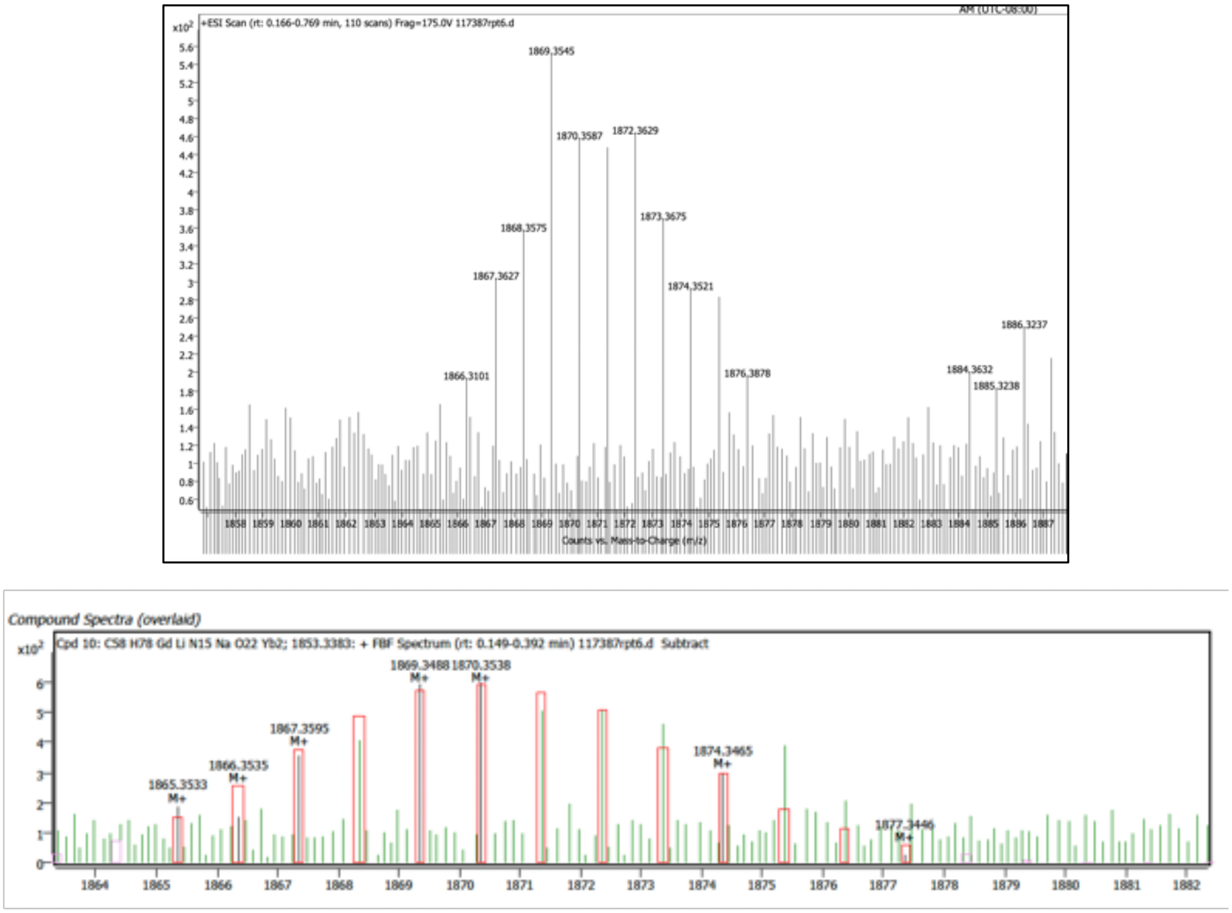

Figure S3. **Electrospray mass spectrum of Yb$_2$Gd recorded in MeOH:H$_2$O in positive mode showing, above the zoomed in experimental spectrum of the [M+Li]$^+$ peak at *m/z* 1869 amu and bottom, overlaid experimental spectrum and calculated isotope pattern.**

Optical Spectra

Note that there is no evidence of uncomplexed metal in solution through the one photon solution spectroscopic experiments.[1] Dissociation of the metals from the is highly unlikely in this system since due to the high kinetic stability of the macrocyclic chelate, there is always a metal bound in this site, and by extension, always a metal in the DTPA binding site as no other competing metals or chelates present.

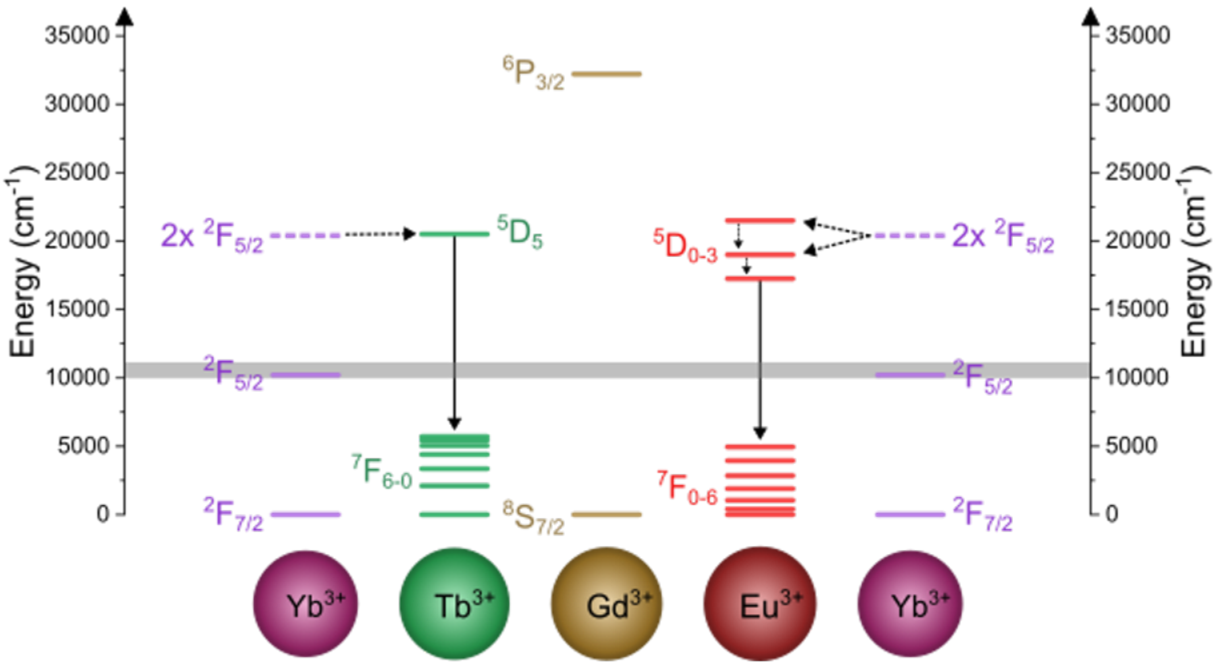

Figure S4. Energy level diagram of all lanthanides used in this study. Lines correspond to the respective lanthanide; Yb^{3+} (Purple); Tb^{3+} (Green); Gd^{3+} (Yellow); Eu^{3+} (Red). Dashed vertical lines correspond to the energy of two Yb^{3+} excited states. Dashed arrows represent energy transfer or non-radiative relaxation, solid arrows represent emission. Grey bar shows approximate excitation energy 960-980 nm (10200-10400 cm^{-1}).

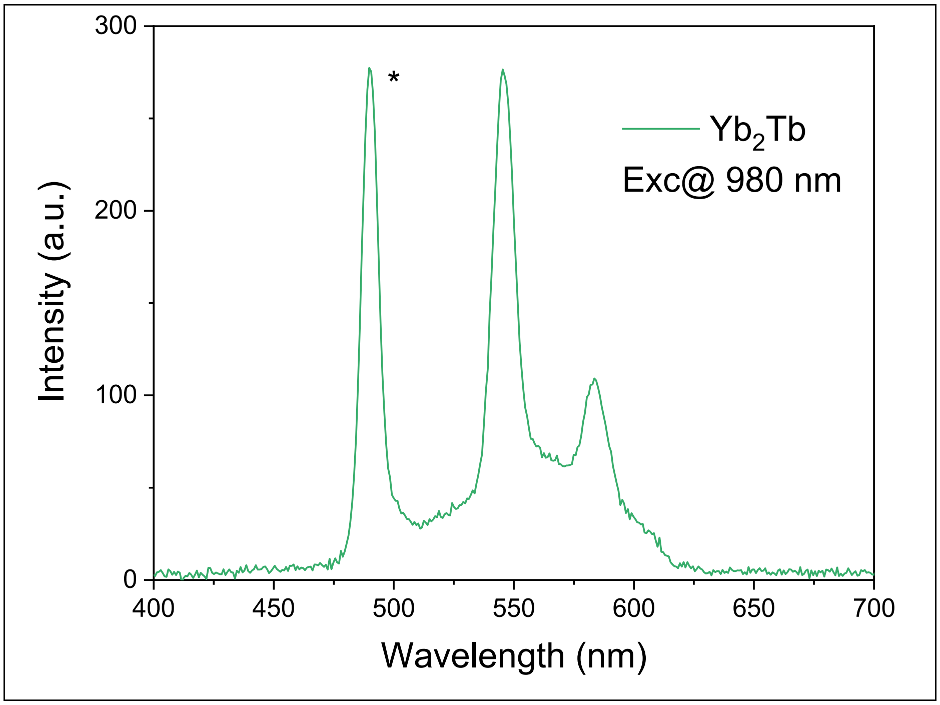

Figure S5. Upconversion emission spectra of Yb_2Tb in D_2O, excited at 980 nm ((laser beam radius = 11.8 ± 0.9 µm). Laser power was kept at 1130 mW. Signal from the second harmonic of the incident laser at 490 nm denoted with *

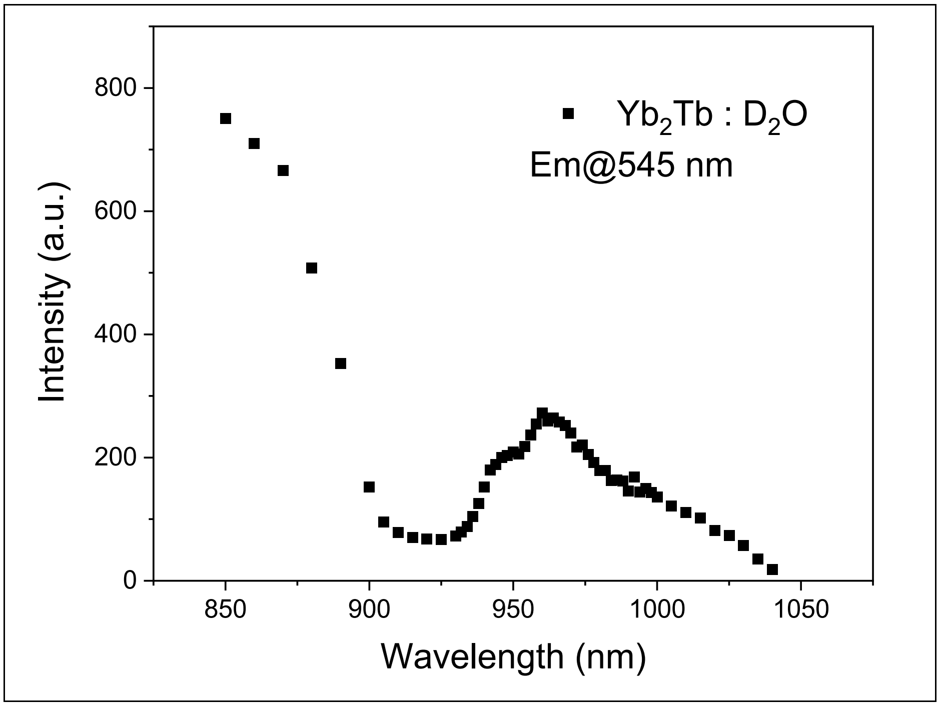

Figure S6. Upconversion excitation spectra of Yb_2Tb in D_2O. Emission measured at 545 nm. Laser power was kept constant at 550 ± 10 mW.

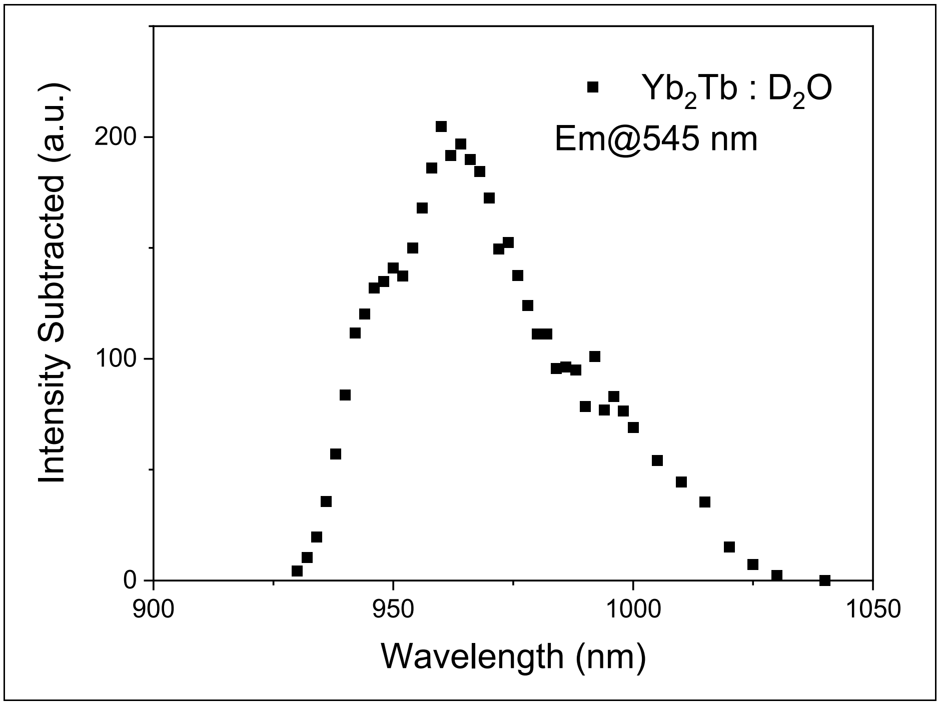

Figure S7. Upconversion excitation spectra of Yb_2Tb in D_2O. Ligand centred signal has been subtracted. Emission measured at 545 nm. Laser power was kept constant at 550 ± 10 mW.

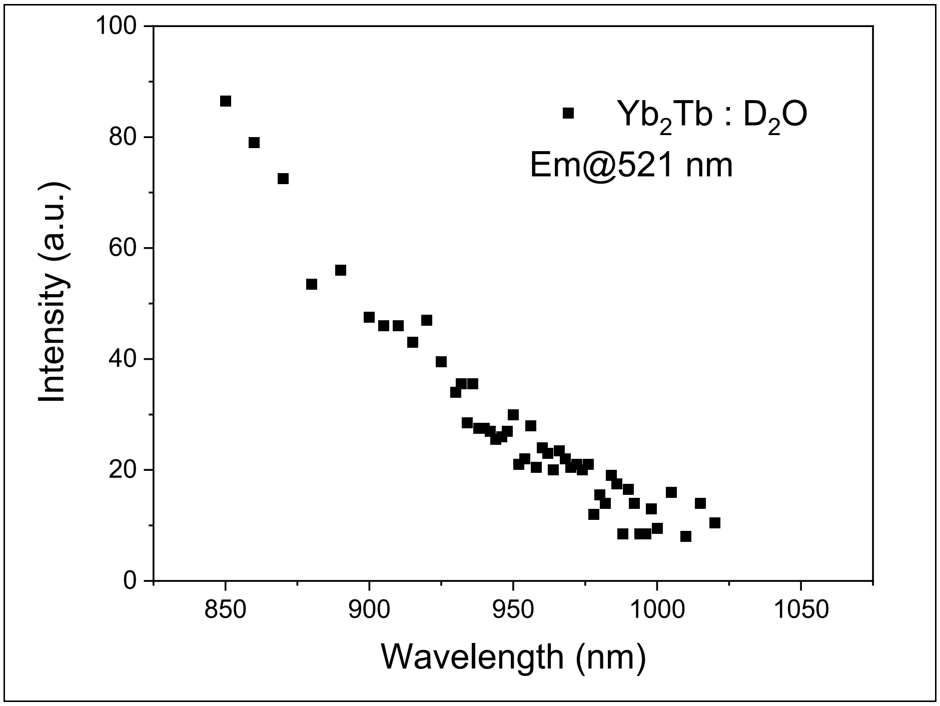

Figure S8. Upconversion excitation spectra of Yb_2Tb in D_2O. Emission measured at 521 nm. Laser power was kept constant at 550 ± 10 mW.

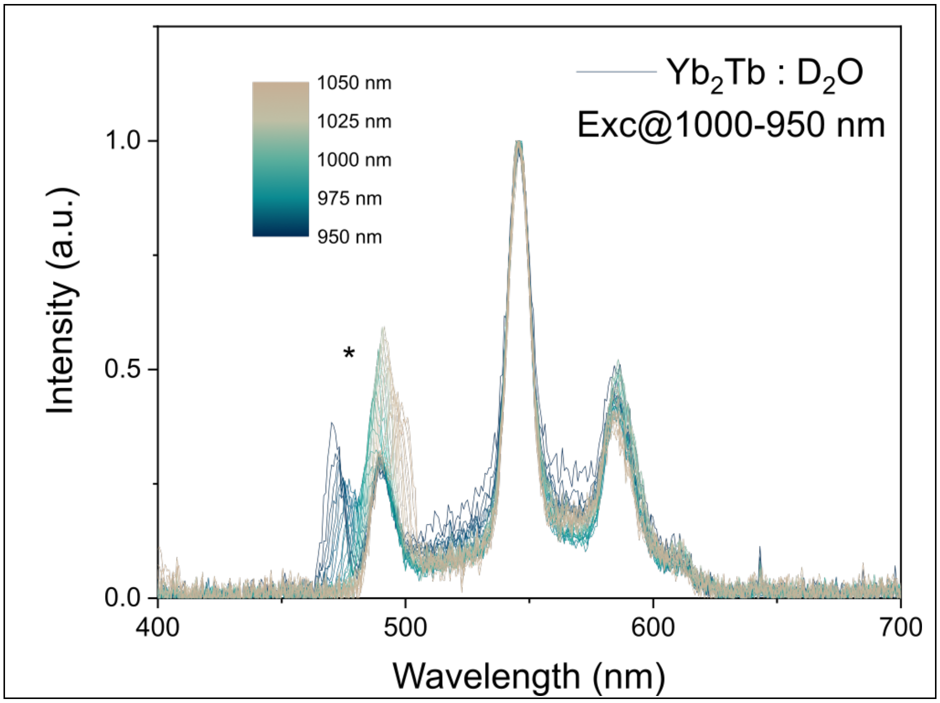

Figure S9. **Normalised emission spectra of Yb_2Tb in D_2O excited at 1000 (grey) nm to 950 (blue) nm in 2 nm intervals. Laser power was kept constant at 550 ± 10 mW. Residual signal from the second harmonic of the incident laser at 475-525 nm denoted with ***

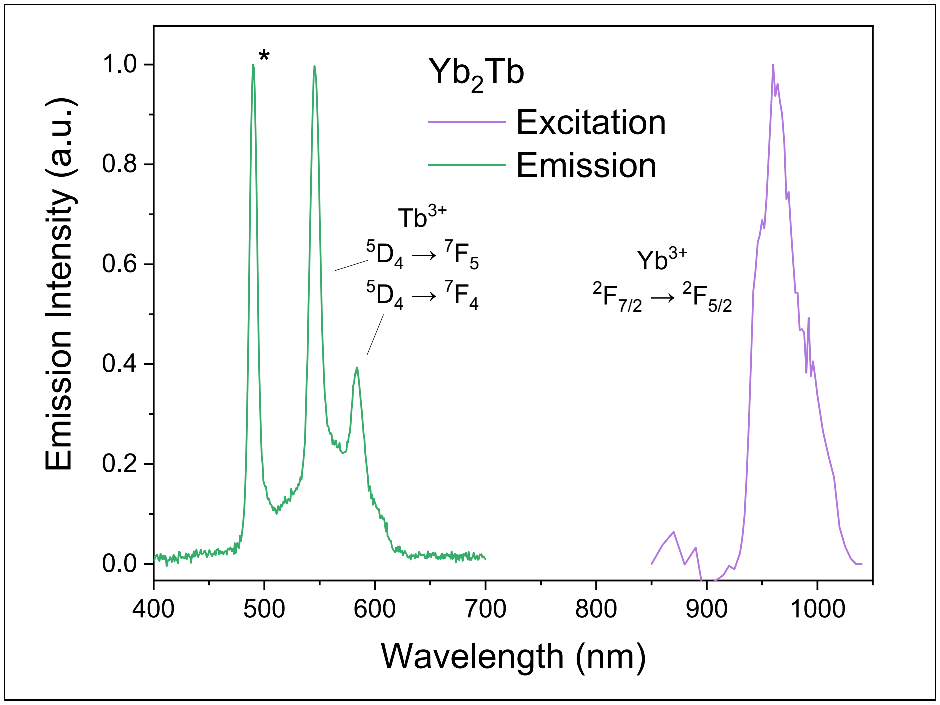

Figure S10. **Upconversion emission and excitation spectra of Yb_2Tb in D_2O. Ligand centred signal has been subtracted in excitation spectra. Emission measured at 545 nm. Excitation at 980 nm (laser beam radius = 11.8 ± 0.9 µm). Laser power was kept at 1130 mW for emission spectra and at 550 ± 10 mW for excitation spectra. Signal from the second harmonic of the excitation source at 490 nm denoted with ***

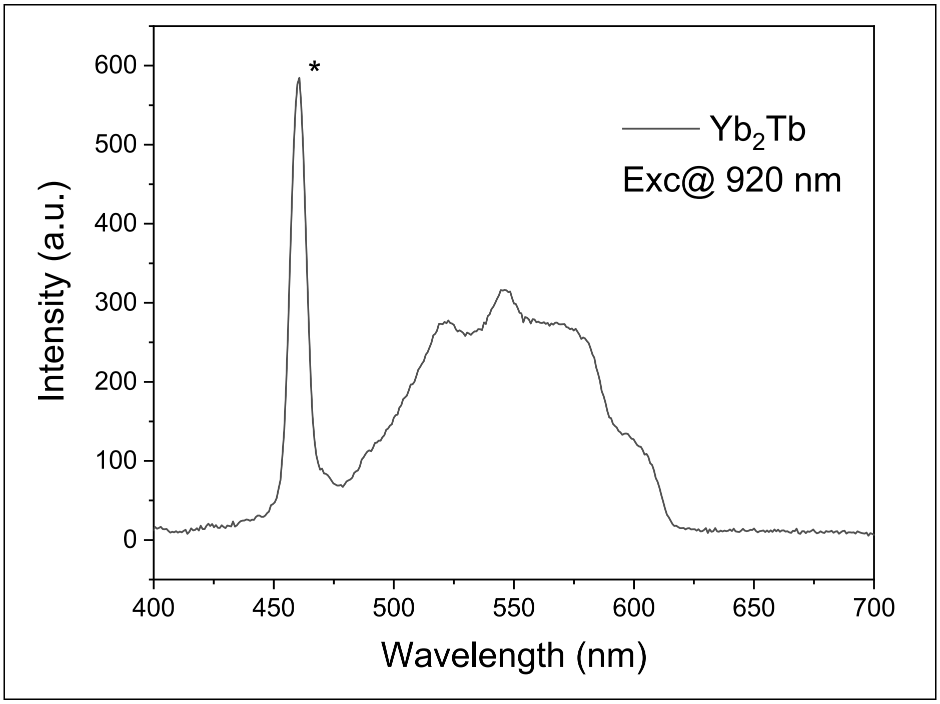

Figure S11. Upconversion emission spectra of Yb_2Tb in D_2O, excited at 920 nm (laser beam radius = 11.1 ± 0.8 µm). Laser power was kept at 1595 mW. Signal from the second harmonic of the excitation source at 460 nm denoted with *

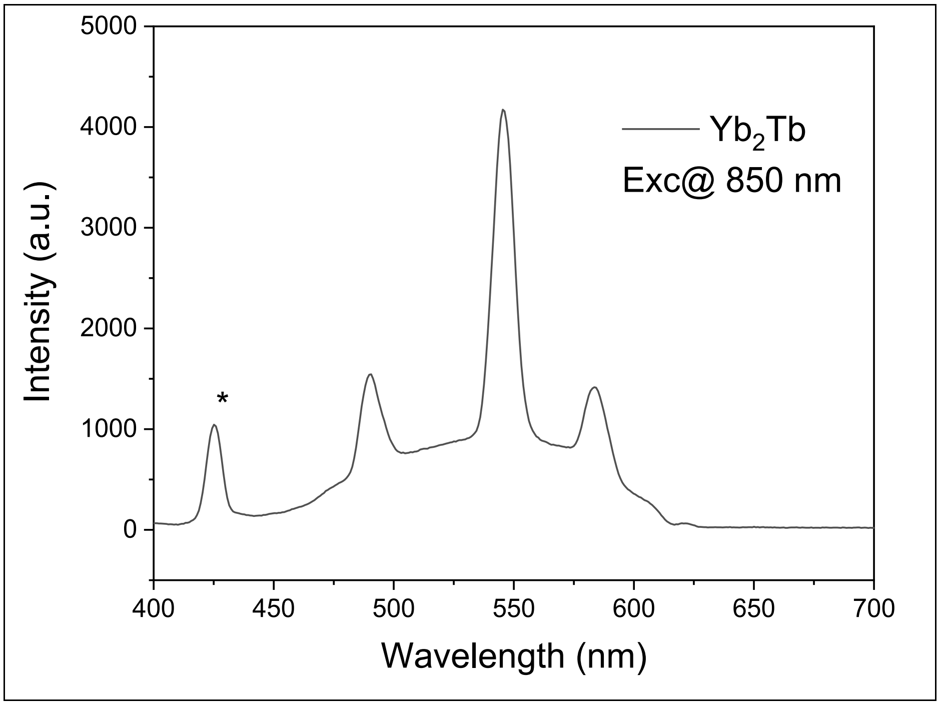

Figure S12. Upconversion emission spectra of Yb_2Tb in D_2O, excited at 850 nm (laser beam radius = 10.7 ± 0.8 µm). Laser power was kept at 2340 mW. Signal from the second harmonic of the excitation source at 425 nm denoted with *.

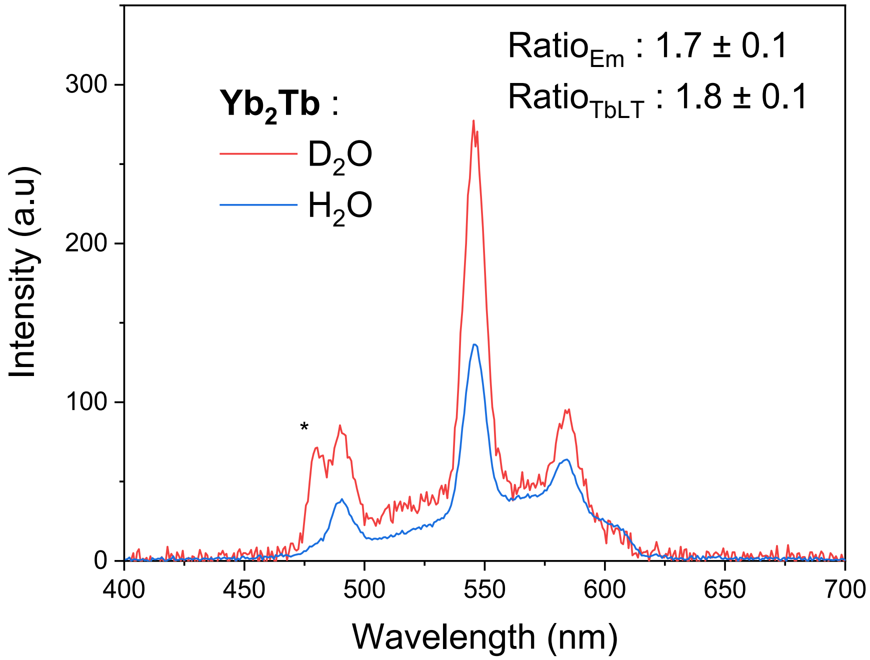

Figure S13. Upconversion emission spectra of Yb_2Tb in D_2O (red) and H_2O (blue) excited at 960 nm, laser beam radius = 11.5 ± 0.9 µm. The laser power was kept at 550 ± 5 mW. $Ratio_{Em}$ was taken as the ratio between the integrated emission intensities. $RatioT_{bLT}$ was taken as the ratio between the single-photon emission lifetimes of Tb^{3+} in D_2O and H_2O (3.0 and 1.7 ms respectively).[1] Signal from the second harmonic of the excitation source at 480 nm denoted with *.

In Figure S13 above, the ratios of the lifetimes, *i.e.* the decay from the Tb^{3+} $^5D_4$ excited state in D_2O and H_2O formed after 1-photon excitation are 1.8 +/- 0.1 (previously reported in by us),[1] while the ratio of upconverted emission intensities is 1.7 +/- 0.1. The 1-photon measurements report on the contribution of the O-H vibrational quenching to the lifetime of the Tb^{3+} $^5D_4$ excited state, while the intensities ratio will also report on any coupling of O-H/D vibrations to the Yb_2→Tb energy transfer process. As these two ratios are not significantly different, we suggest that Yb_2→Tb energy transfer appears to be independent of OH quenching, which we have rephrased in the manuscript for clarity.

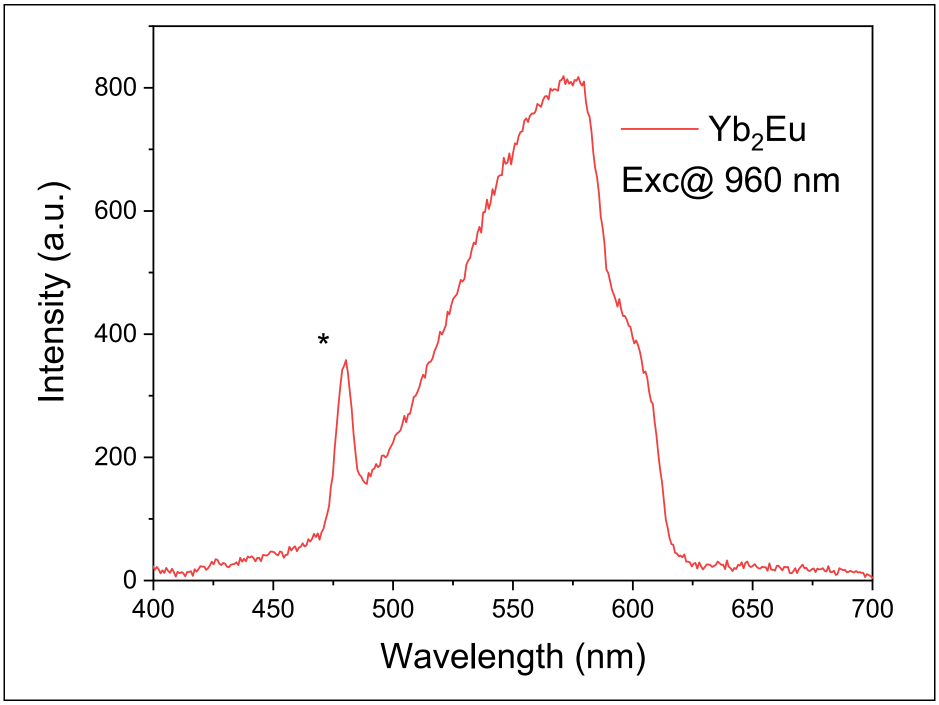

Figure S14. Upconversion emission spectra of Yb_2Eu in D_2O, excited at 960 nm (laser beam radius = 11.5 ± 0.9 µm). Laser power was kept at 1304 mW. Signal from the second harmonic of the excitation source at 480 nm denoted with *.

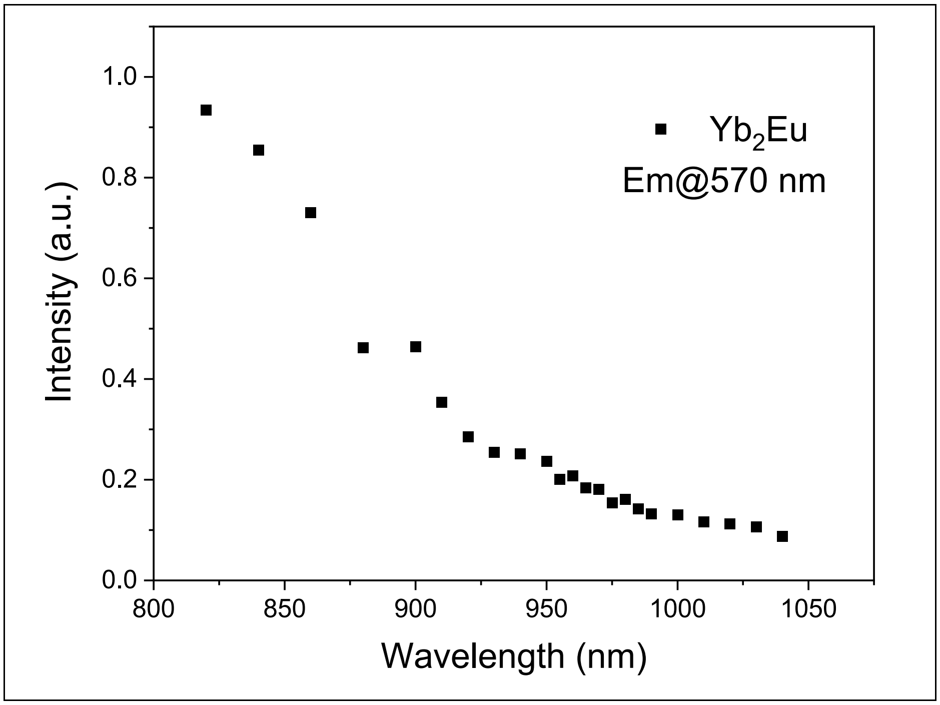

Figure S15. Upconversion excitation spectra of Yb_2Eu in D_2O. Emission measured at 570 nm. Laser power was kept constant at 550 ± 10 mW.

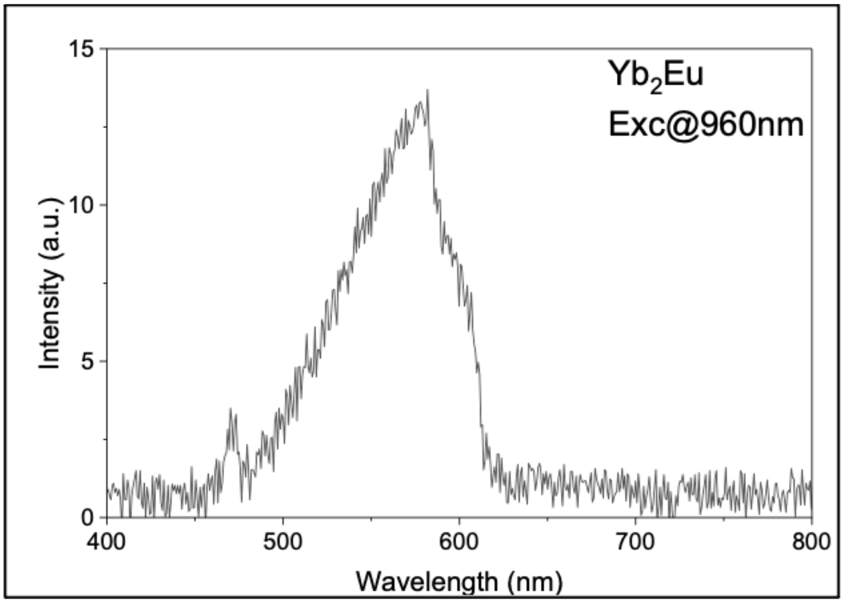

Figure S16. Upconversion emission spectra of Yb_2Eu in degassed, argon purged H_2O, excited at 960 nm (laser beam radius = 11.5 ± 0.9 µm). The feature at 480 nm is due to the second harmonic of the excitation source.

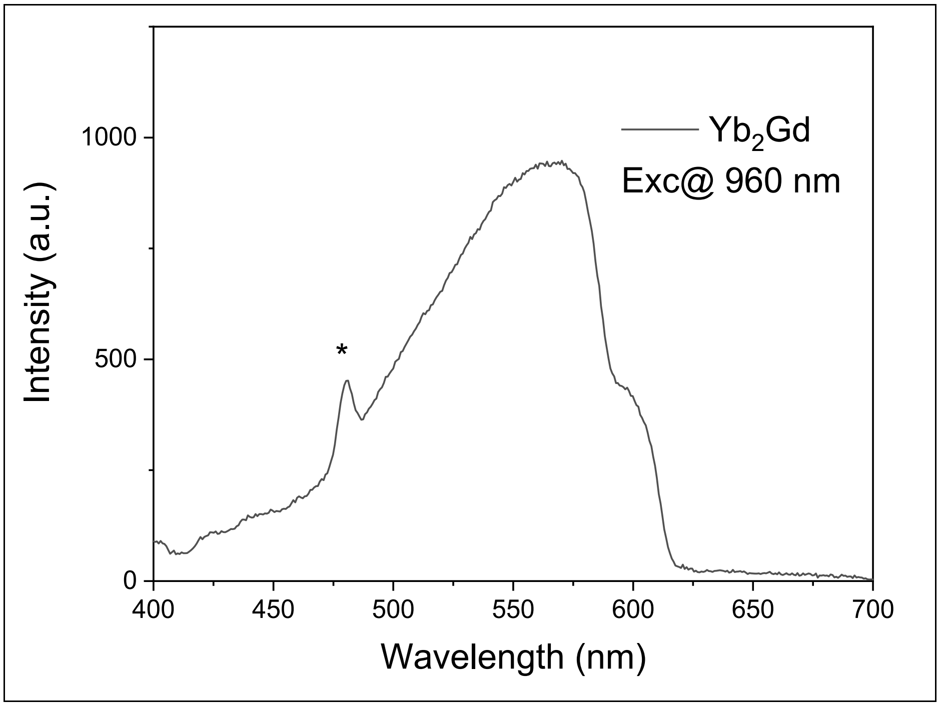

Figure S17. Upconversion emission spectra of Yb_2Gd in D_2O, excited at 960 nm (laser beam radius = 11.5 ± 0.9 µm). Laser power was kept at 1300 mW. Signal from the second harmonic of the excitation source at 480 nm denoted with *.

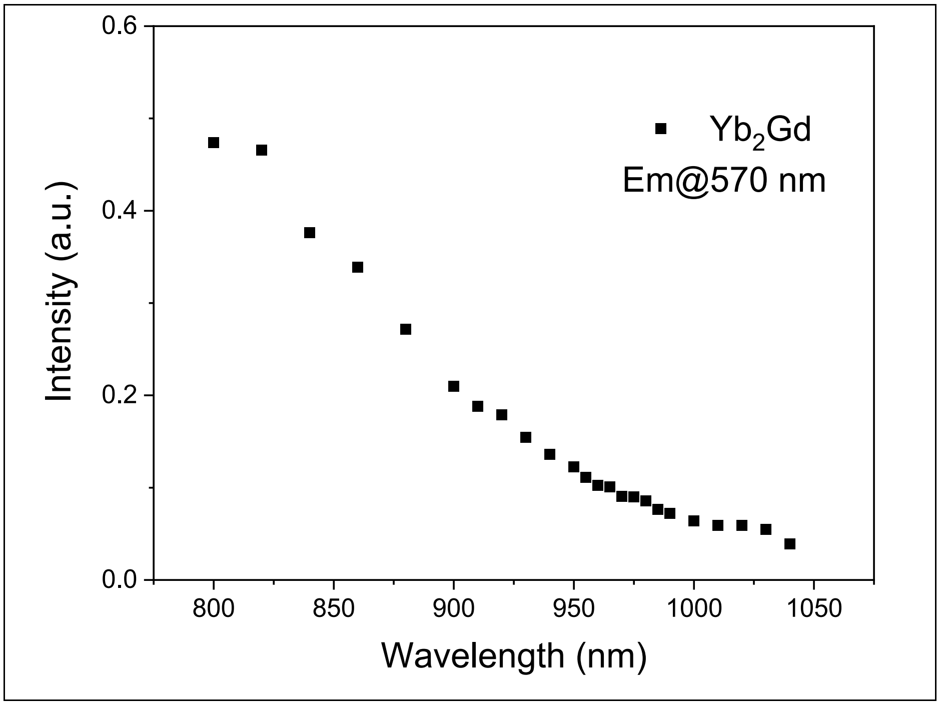

Figure S18. Upconversion excitation spectra of Yb_2Gd in D_2O. Emission measured at 570 nm. Laser power was kept constant at 550 ± 10 mW.

Power Dependence Measurements

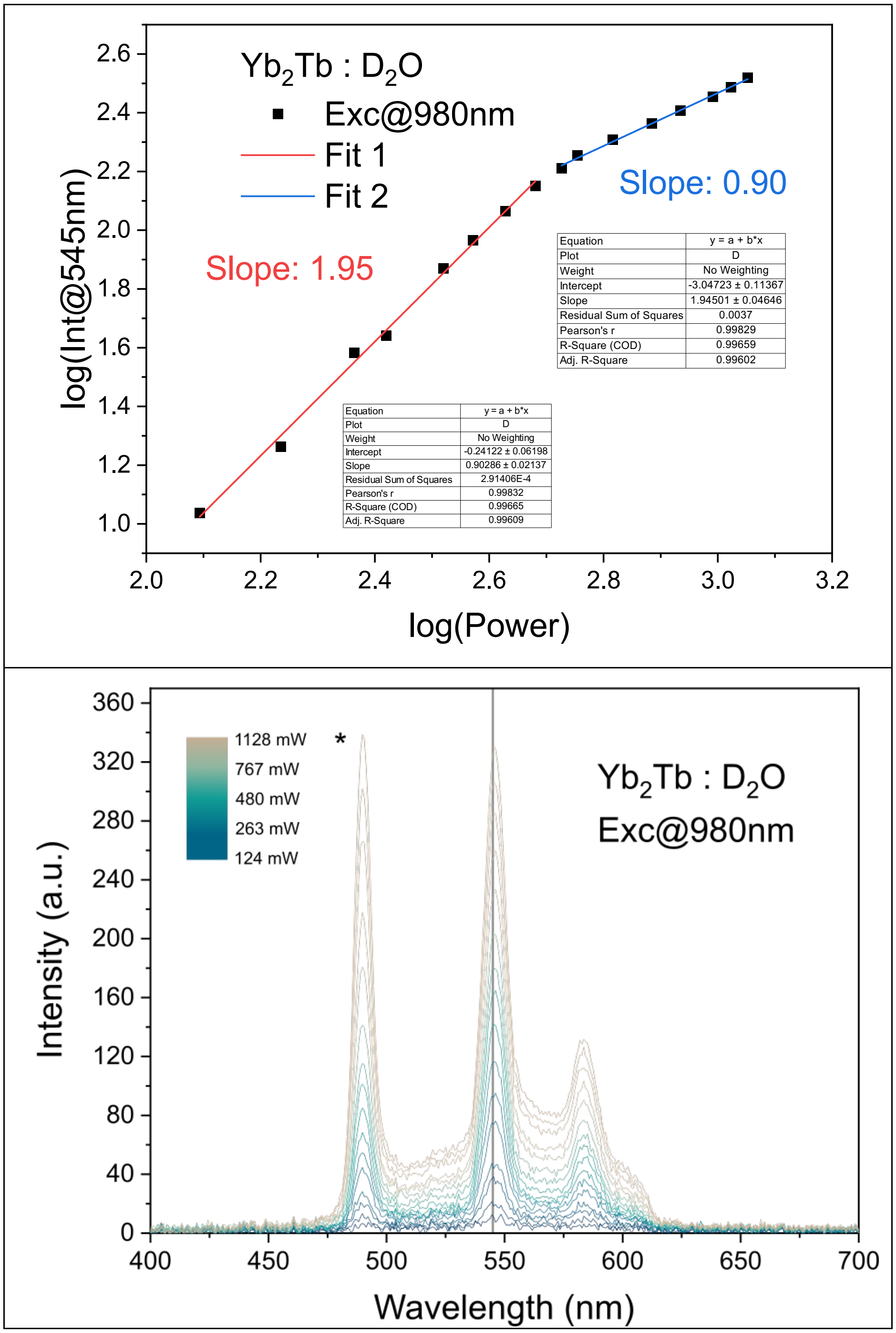

Figure S19. Top: Power dependence of upconversion emission at 545 nm for Yb$_2$Tb in D$_2$O, excited at 980 nm (laser beam radius = 11.8 ± 0.9 μm). The data has been fitted with two linear functions. Bottom: Upconversion emission spectra excited at 980 nm. Laser signal is denoted with *. The vertical line indicates wavelength (545 nm) used for power dependence determination. Laser power range was 124-1128 mW.

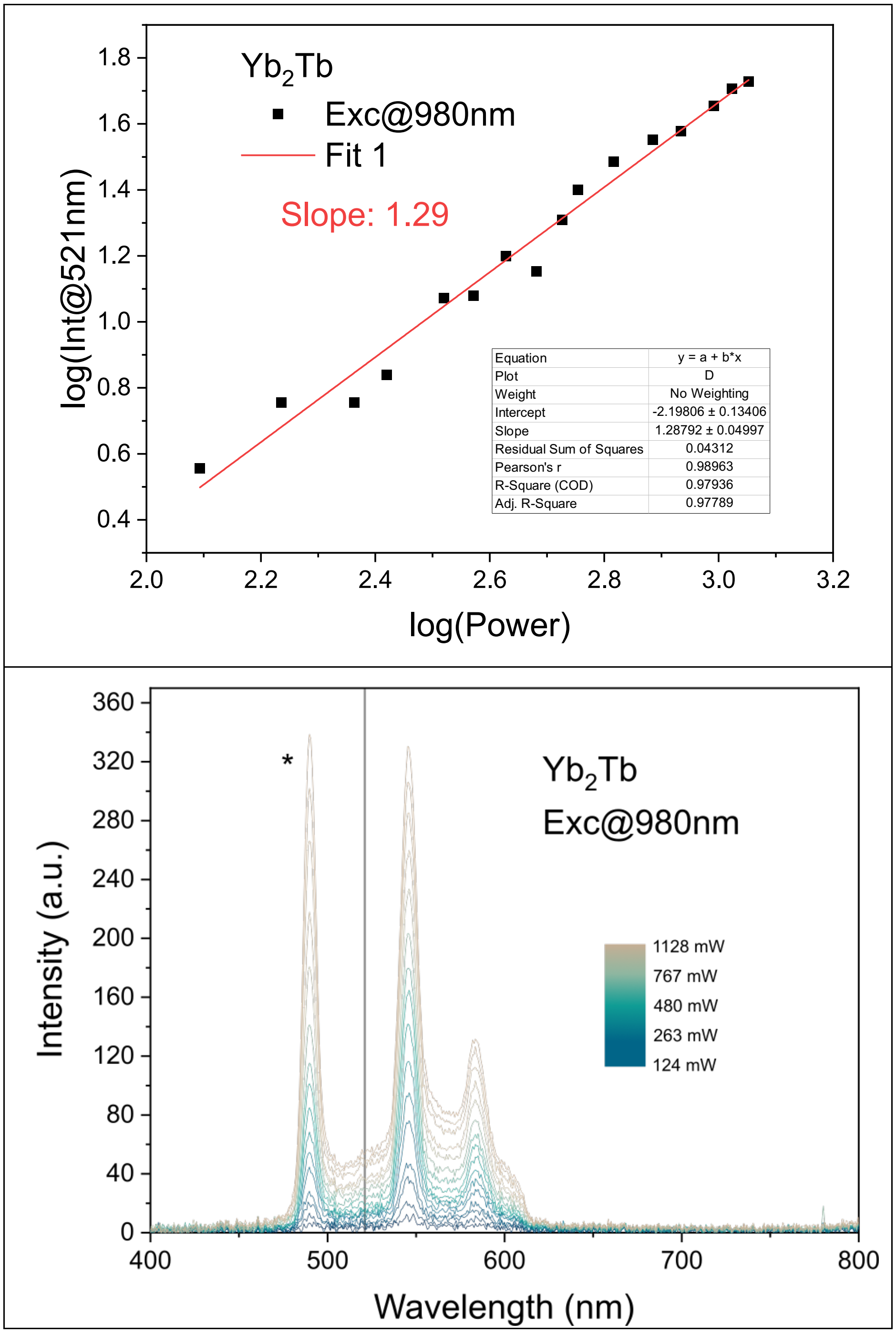

Figure S20. Top: Power dependence of upconversion emission at 521 nm for Yb_2Tb in D_2O, excited at 980 nm (laser beam radius = 11.8 ± 0.9 µm). The data has been fitted with one linear function. Bottom: Upconversion emission spectra excited at 980 nm. Residual laser signal from the second harmonic at 490 nm is denoted with *. The vertical line indicates wavelength (521 nm) used for power dependence determination. Laser power range was 124-1128 mW.

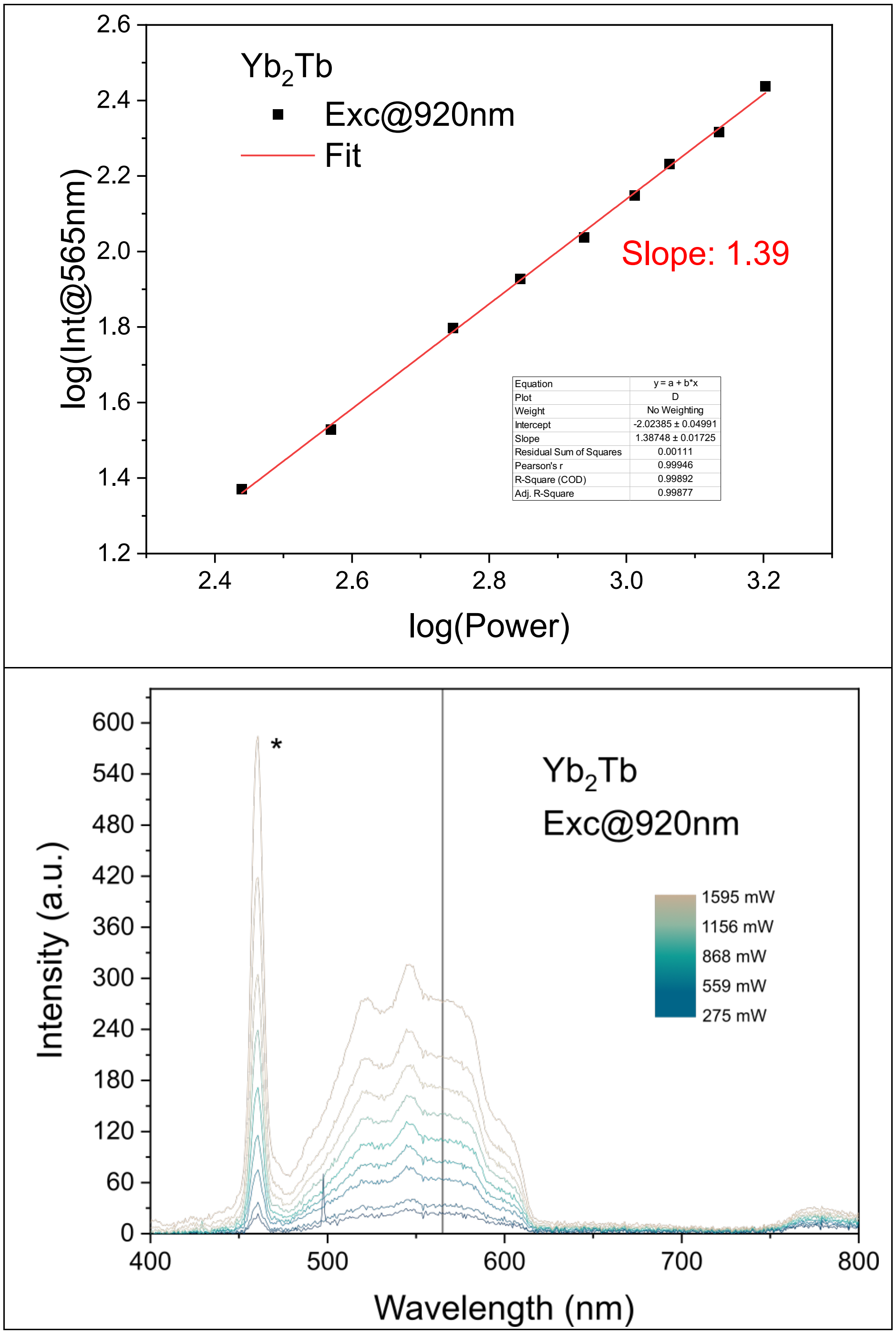

Figure S21. Top: Power dependence of upconversion emission at 565 nm for Yb_2Tb in D_2O, excited at 920 nm (laser beam radius = 11.1 ± 0.8 µm). The data has been fitted with one linear function. Bottom: Upconversion emission spectra excited at 920 nm. Second harmonic laser signal at 460 nm is denoted with *. The vertical line indicates wavelength (565 nm) used for power dependence determination. Laser power range was 275-1595 mW.

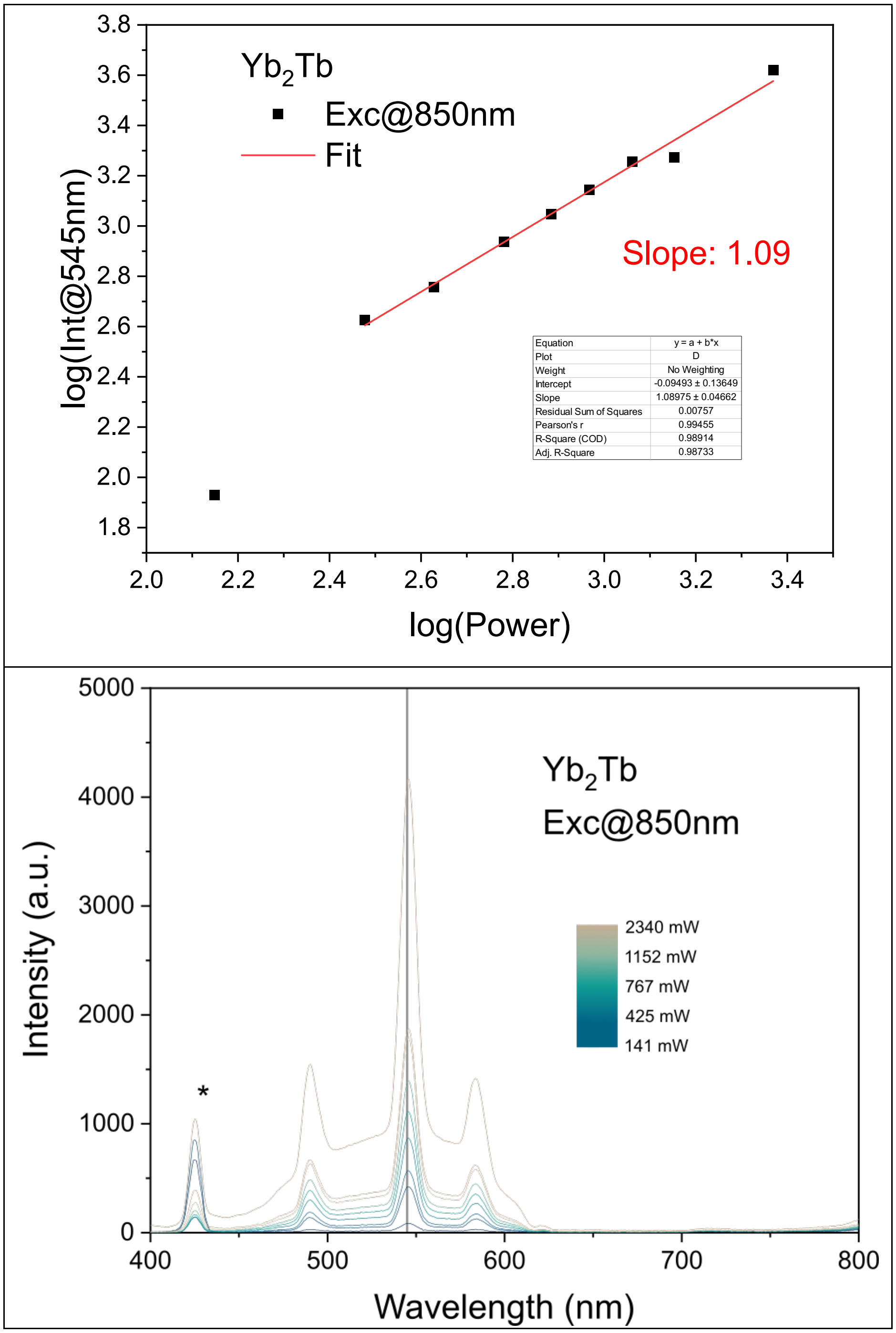

Figure S22. Top: Power dependence of upconversion emission at 545 nm for Yb_2Tb in D_2O, excited at 850 nm (laser beam radius = 10.7 ± 0.8 µm). The data has been fitted with one linear function. Bottom: Upconversion emission spectra excited at 850 nm. The second harmonic from the laser excitation at 425 nm is denoted with *. The vertical line indicates wavelength (545 nm) used for power dependence determination. Laser power range was 141-2340 mW.

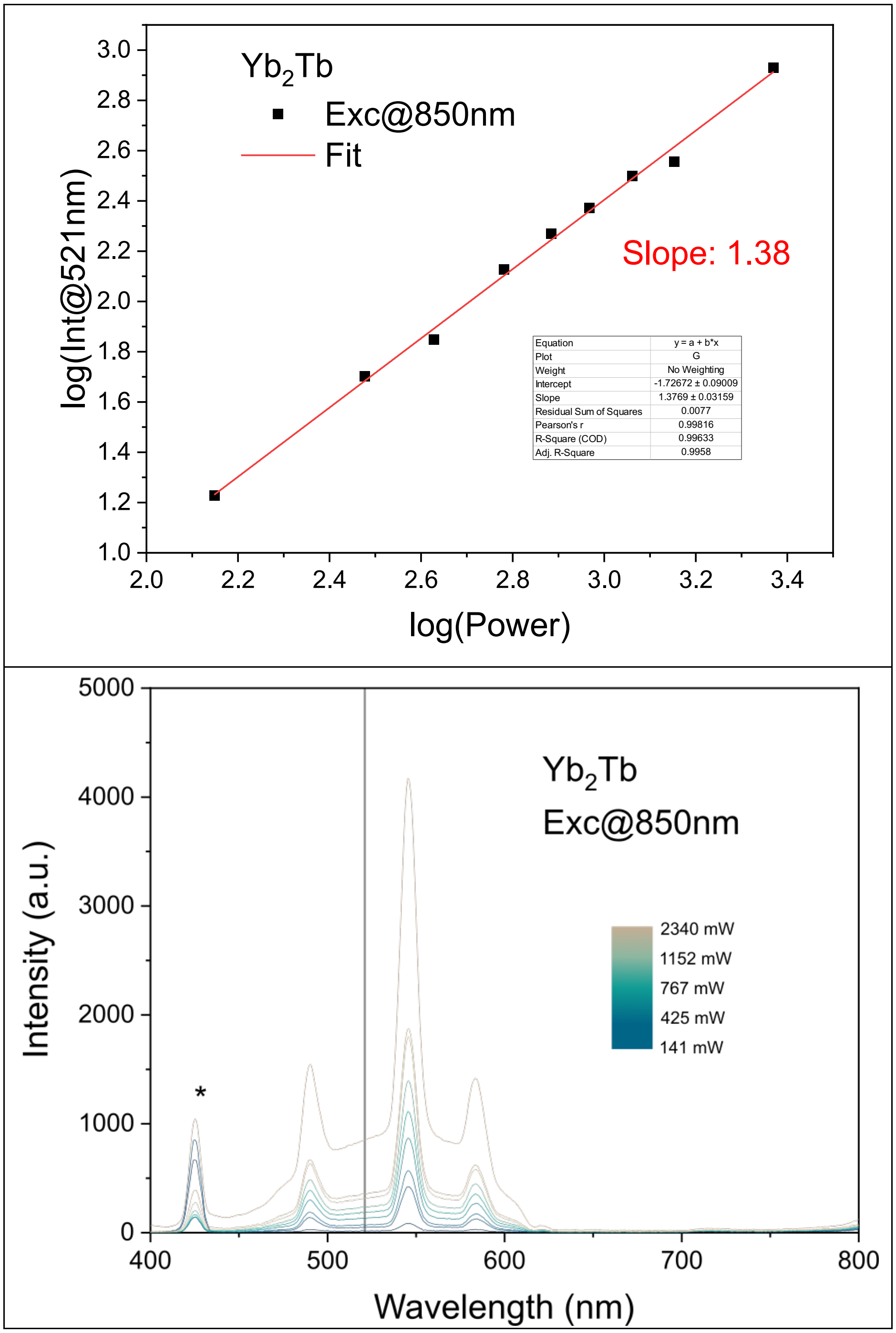

Figure S23. Top: Power dependence of upconversion emission at 521 nm for Yb_2Tb in D_2O, excited at 850 nm (laser beam radius = 10.7 ± 0.8 µm). The data has been fitted with one linear function. Bottom: Upconversion emission spectra excited at 850 nm (laser beam radius = 10.7 ± 0.8 µm). The second harmonic of the laser signal at 425 nm is denoted with *. The vertical line indicates wavelength (521 nm) used for power dependence determination. Laser power range was 141-2340 mW.

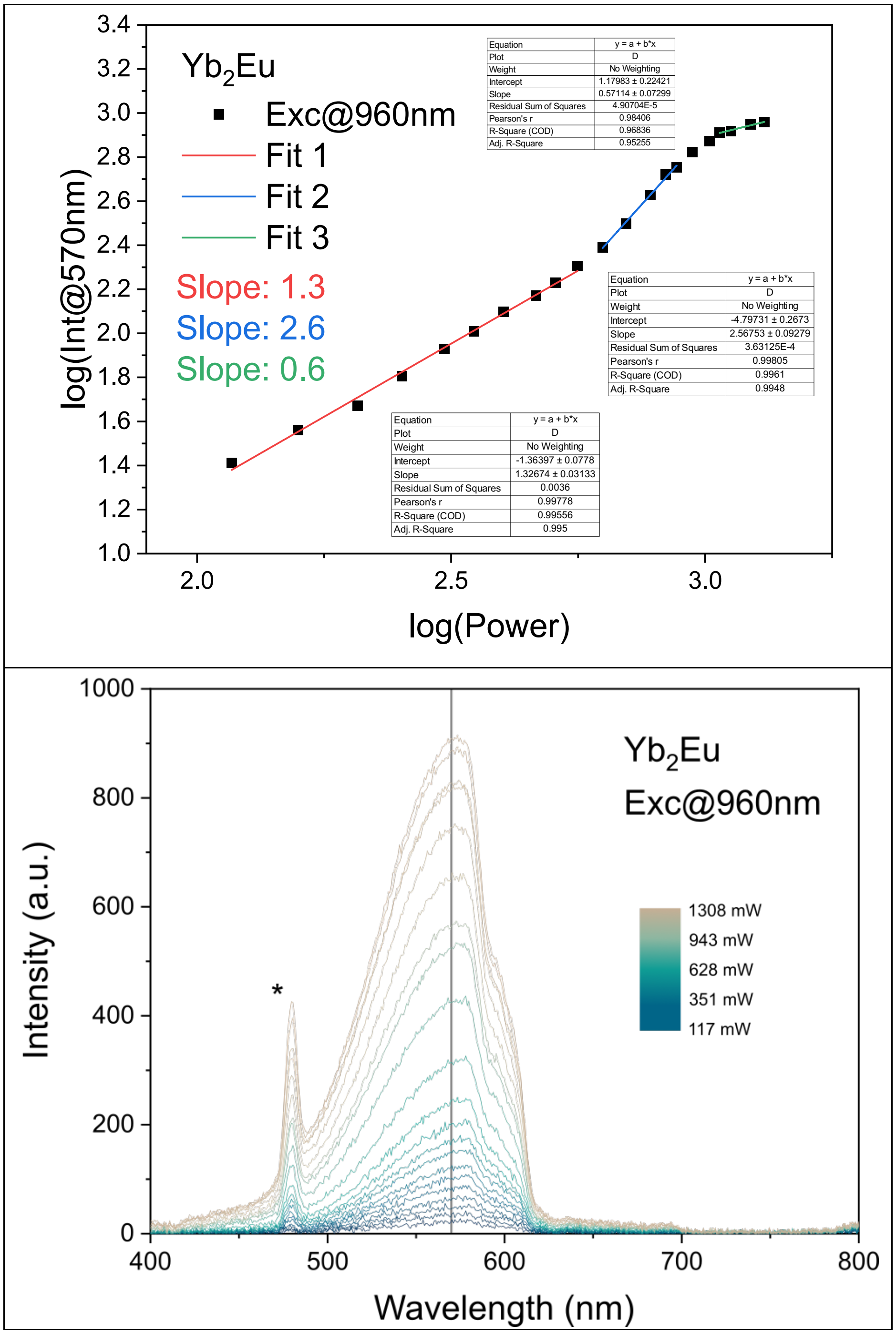

Figure S24. Top: Power dependence of upconversion emission at 570 nm for Yb_2Eu in D_2O, excited at 960 nm (laser beam radius = 11.5 ± 0.9 µm). The data has been fitted with three linear functions. Bottom: Upconversion emission spectra excited at 960 nm. Second harmonic of the laser at 480 nm is denoted with *. The vertical line indicates wavelength (570 nm) used for power dependence determination. Laser power range was 117-1308 mW.

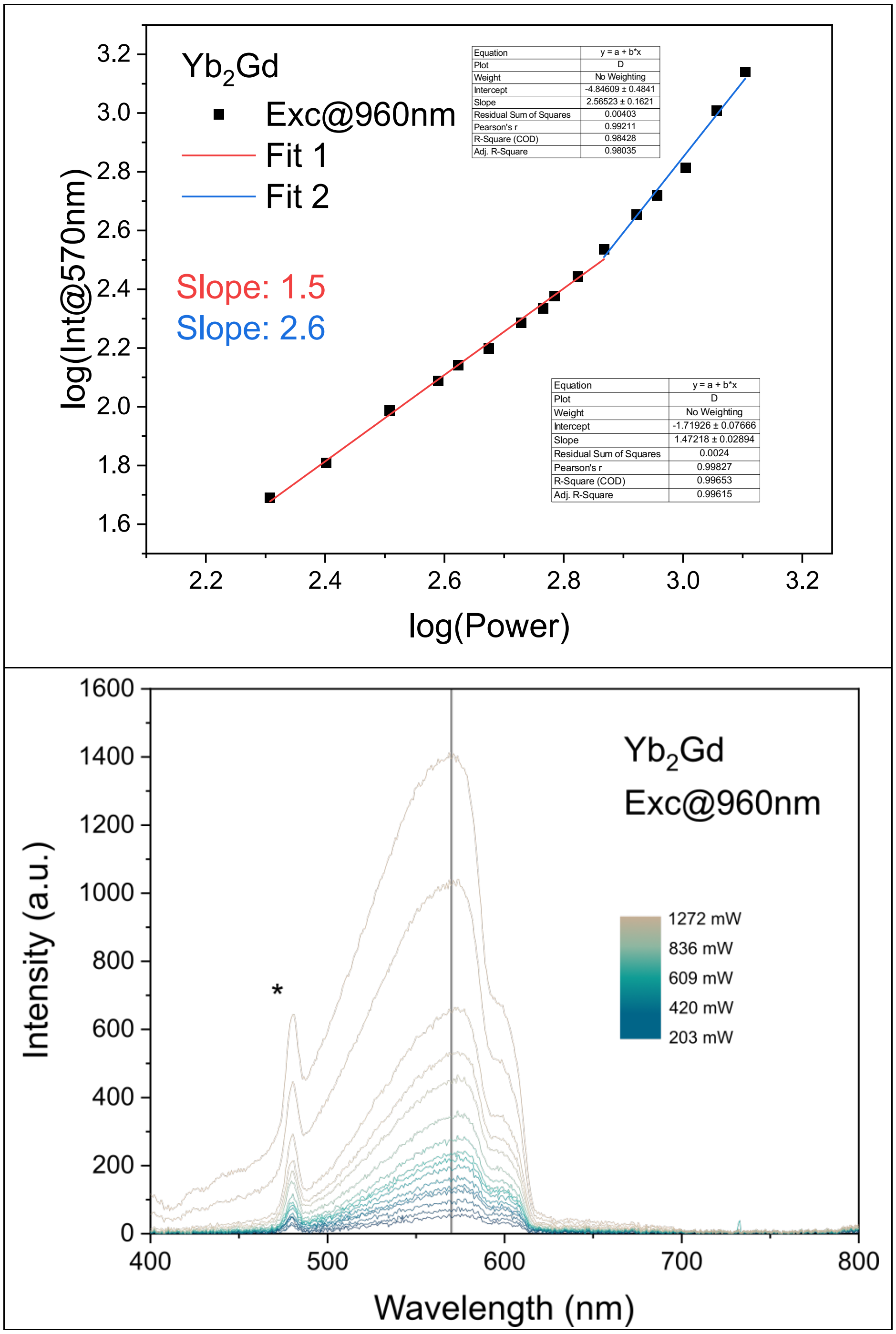

Figure S25. Top: Power dependence of upconversion emission at 570 nm for Yb_2Gd in D_2O, excited at 960 nm (laser beam radius = 11.5 ± 0.9 µm). The data has been fitted with two linear functions. Bottom: Upconversion emission spectra excited at 960 nm. Laser signal is denoted with *. The vertical line indicates wavelength (570 nm) used for power dependence determination. Laser power range was 203-1272 mW.

Time-Resolved Luminescence

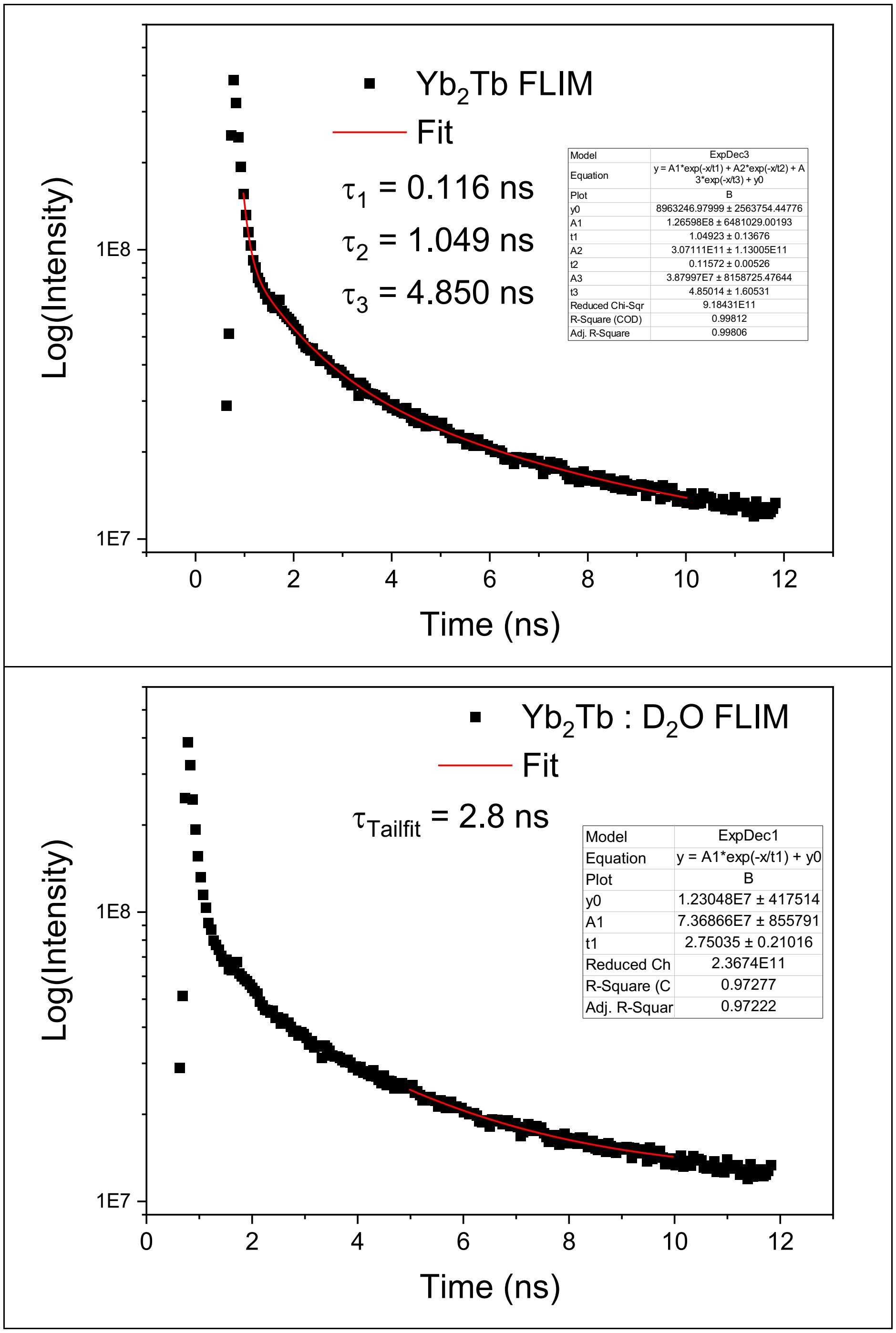

Figure S26. Time-resolved fluorescence decay of Yb_2Tb in D_2O excited at 960 nm. Top: Data was fitted with a tri-exponential decay function. Bottom: Data were fitted with a mono-exponential decay function in the tail-end of the emission decay profile (5-10 ns)

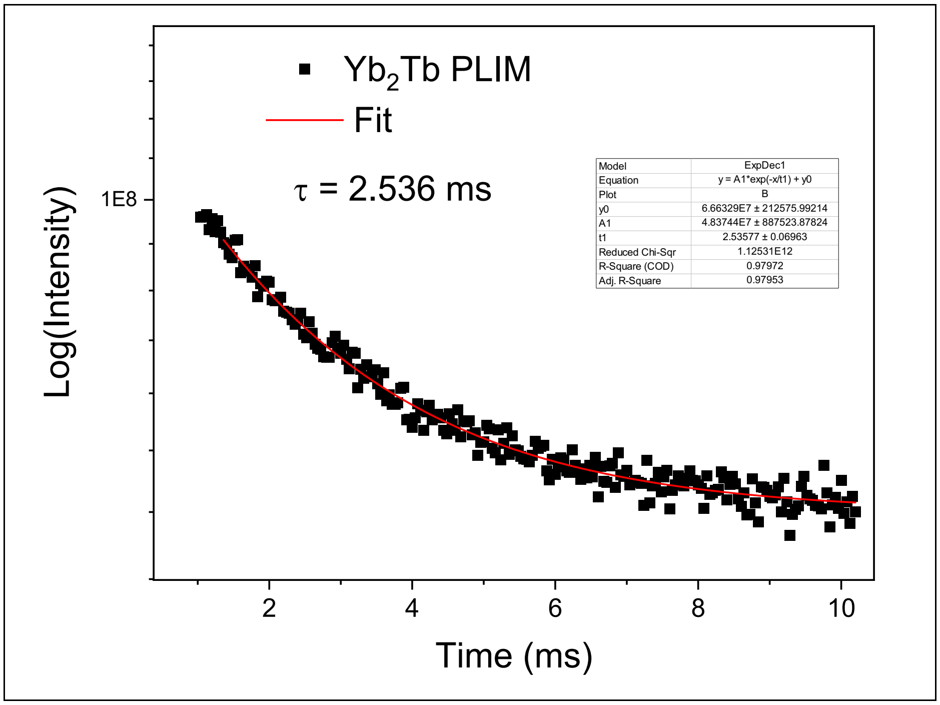

Figure S27. Time-resolved emission decay of long-lived emission of Yb_2Tb in D_2O excited at 960 nm. Data were fitted with a mono-exponential decay function.

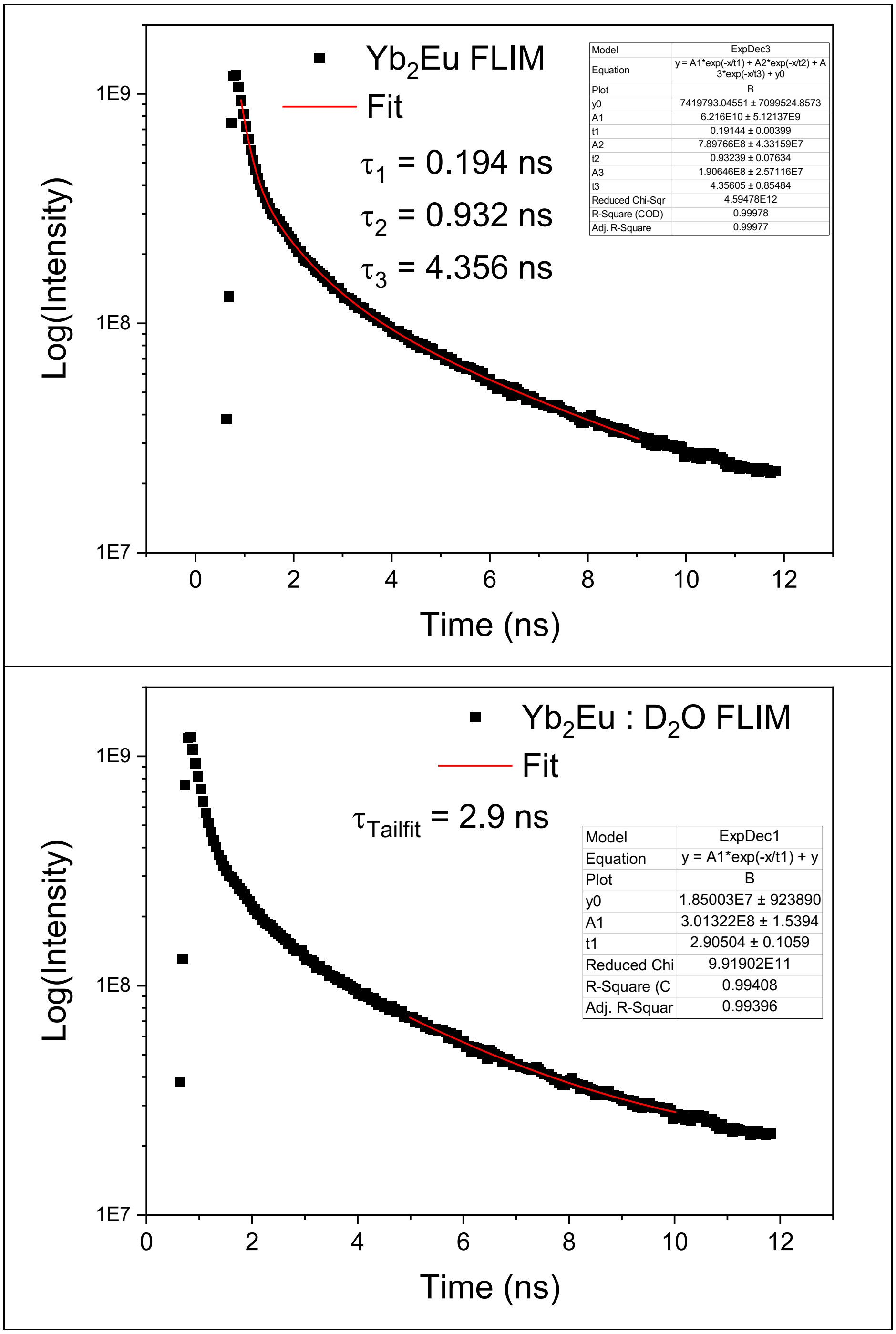

Figure S28. Time-resolved fluorescence decay of Yb_2Eu in D_2O excited at 960 nm. Top: Data were fitted with a tri-exponential decay function. Bottom: Data was fitted with a mono-exponential decay function in the tail-end of the emission decay profile (5-10 ns)

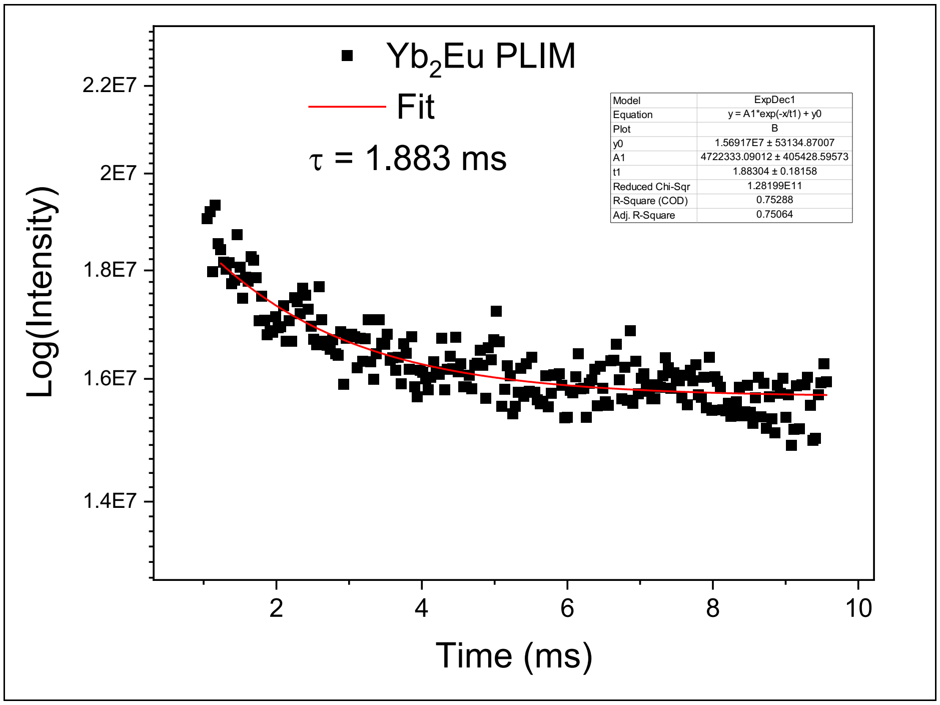

Figure S29. Time-resolved emission decay of long-lived emission of Yb_2Eu in D_2O excited at 960 nm. Data were fitted with a mono-exponential decay function.

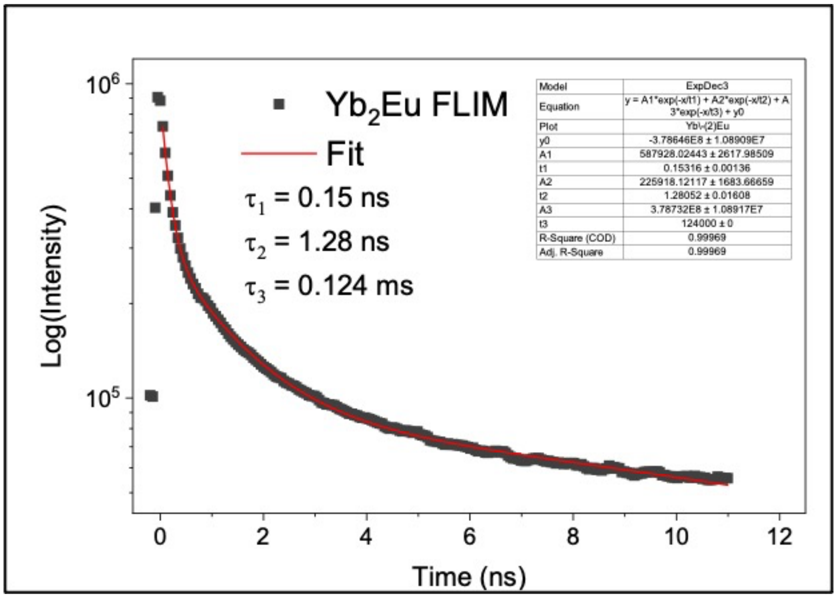

Figure S30. Time-resolved fluorescence decay of Yb_2Eu in degassed, argon purged H_2O solution excited at 960 nm. Data were fitted with a tri-exponential decay function with two short nanosecond components and a long lived (essentially flat) microsecond component estimated as 124 μs, which was not fully captured by the FLIM time window used.

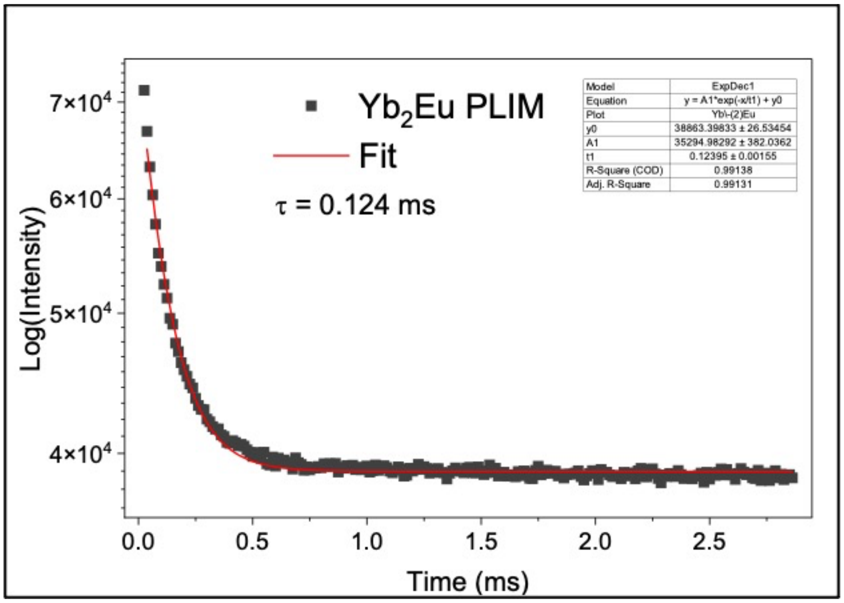

Figure S31. Time-resolved phosphorescence decay of Yb_2Eu in degassed, argon purged H_2O solution excited at 960 nm. Data were tail fitted with a mono-exponential decay function and no improvement of fit was observed when a second decay constant was added to the fit.

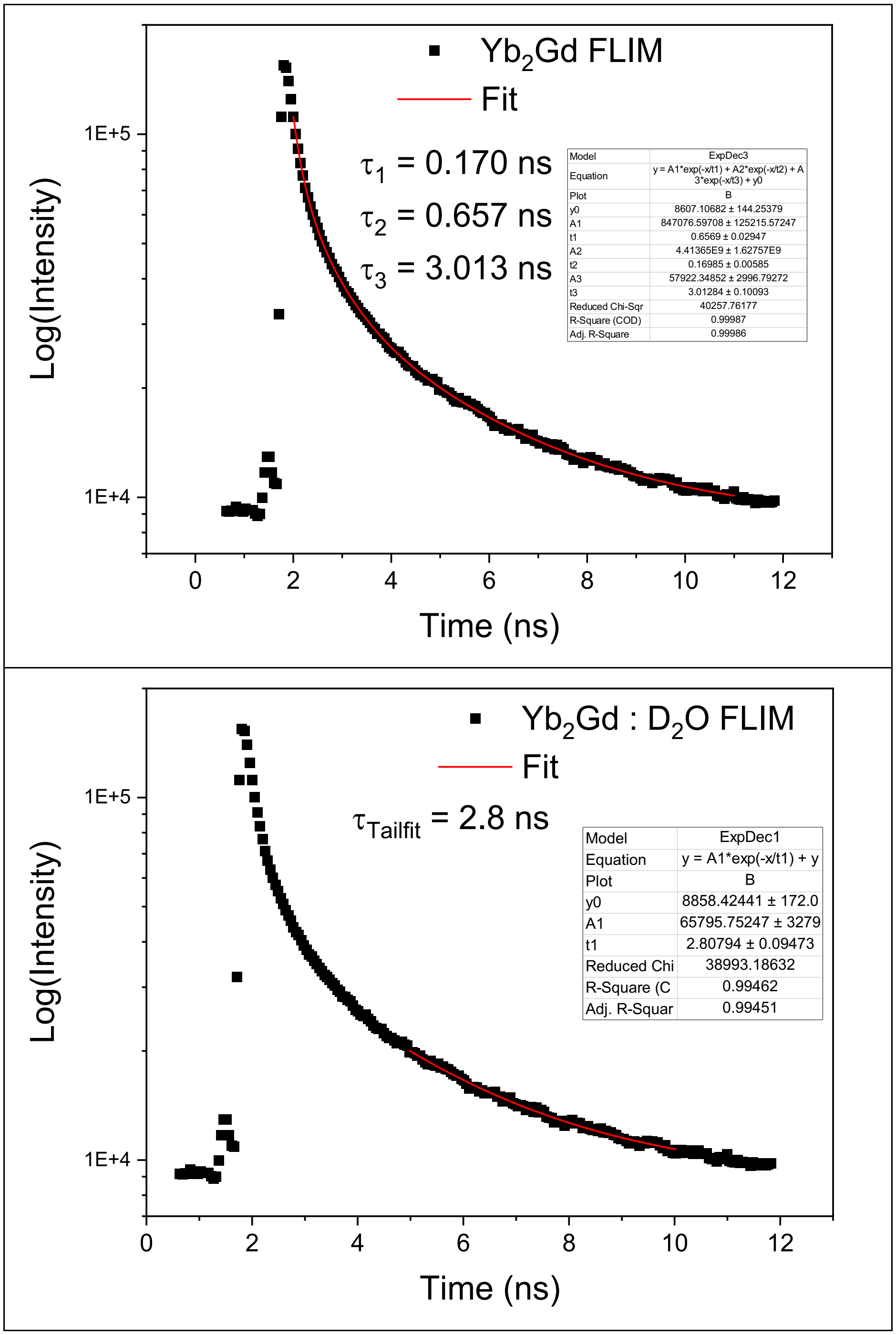

Figure S32. Time-resolved fluorescence decay of Yb_2Gd in D_2O excited at 960 nm. Top: Data were fitted with a tri-exponential decay function. Bottom: Data were fitted with a mono-exponential decay function in the tail-end of the emission decay profile (5-10 ns)

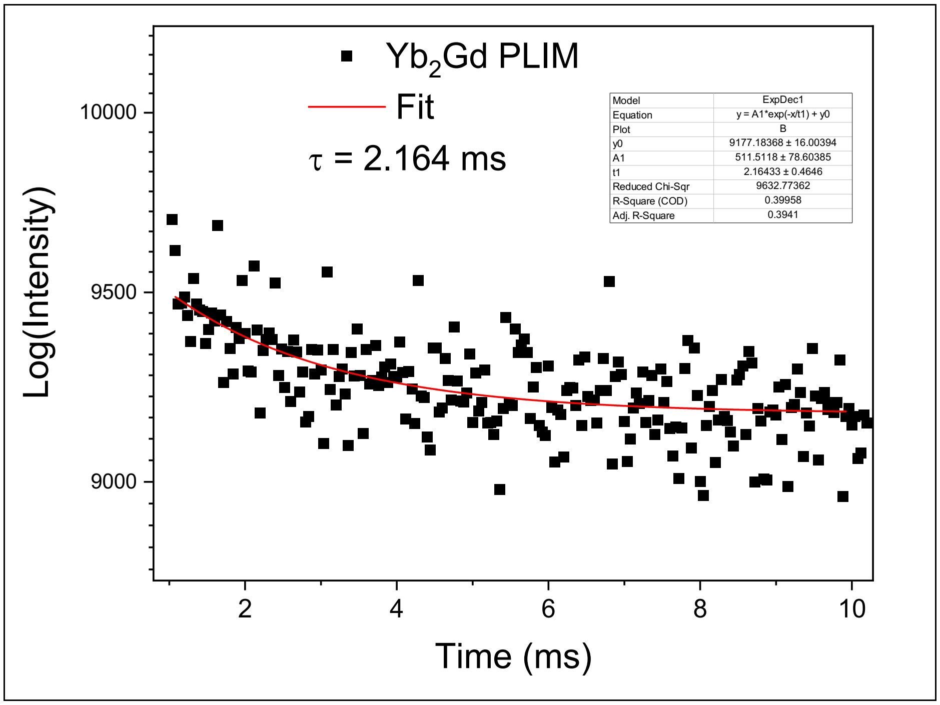

Figure S33. Time-resolved emission decay of long-lived emission of Yb_2Gd in D_2O excited at 960 nm. Data were fitted with a mono-exponential decay function.

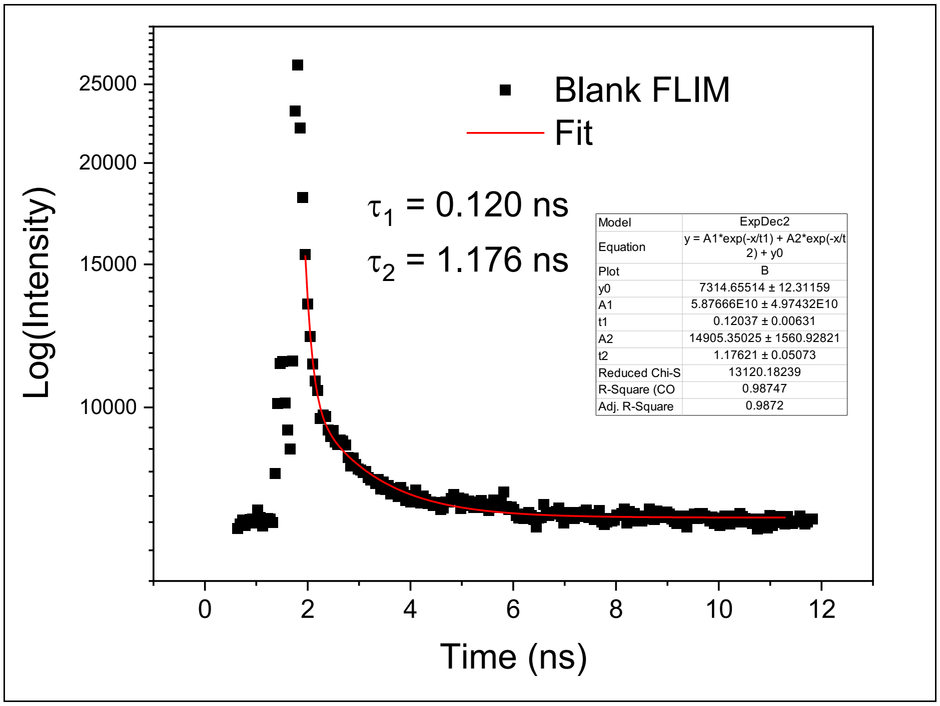

Figure S34. Time-resolved fluorescence decay of D_2O (Blank) excited at 960 nm. Data were fitted with a bi-exponential decay function.

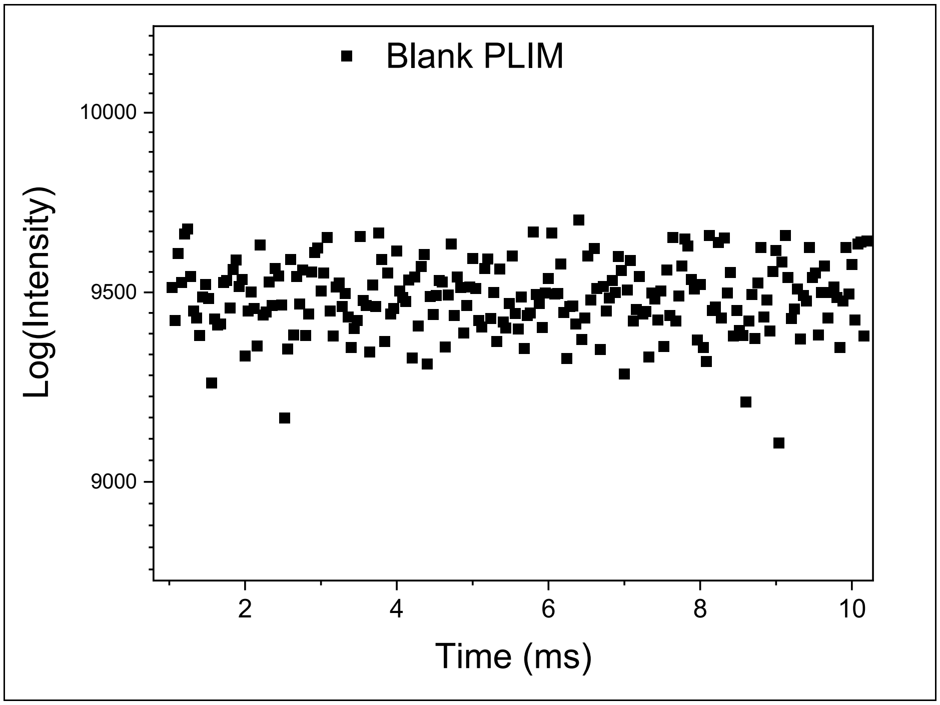

Figure S35. Time-resolved emission decay of long-lived emission of D_2O (Blank) excited at 960 nm. No signal was observed.

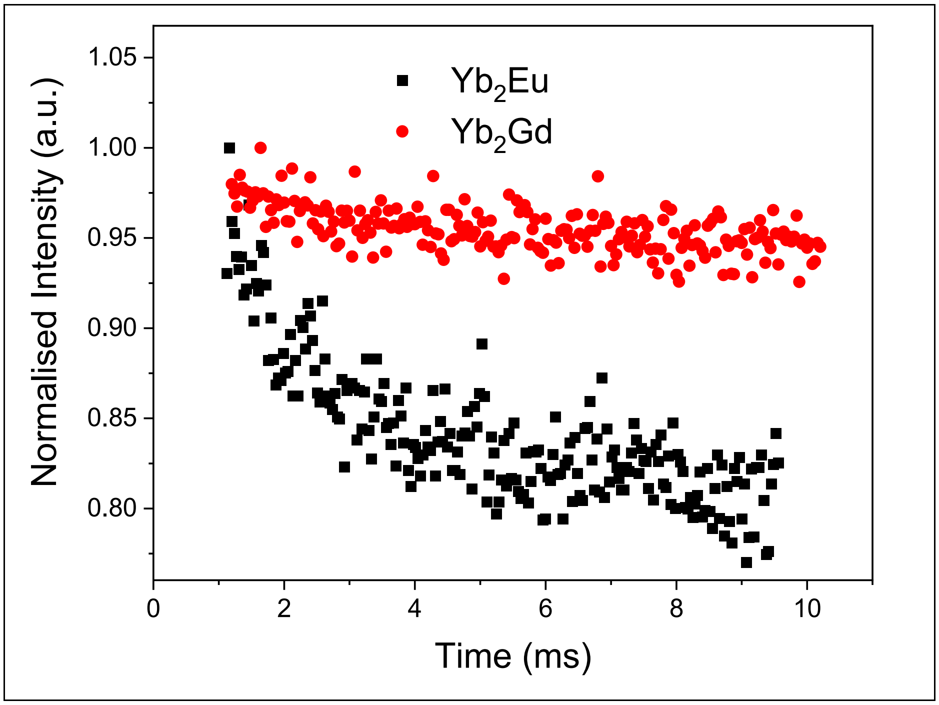

Figure S36. Normalised time-resolved emission decay of long-lived emission of Yb_2Eu and Yb_2Gd in D_2O.

H_2O / D_2O

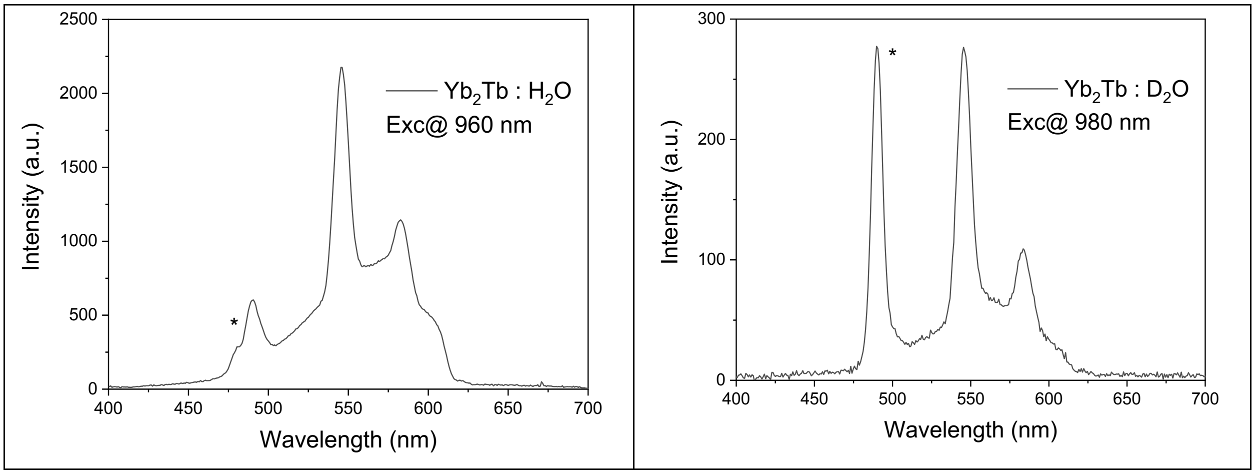

Figure S37. Emission spectra of Yb_2Tb in H_2O (left) and D_2O (right) excited at 960 (and 980 nm respectively. Laser power was kept at 1300 and 1130 mW for sample in H_2O and D_2O respectively. Signal from excitation source is marked with *. The Tb emission signal is independent of excitation wavelength between 1000-950 nm (see Figure S9).

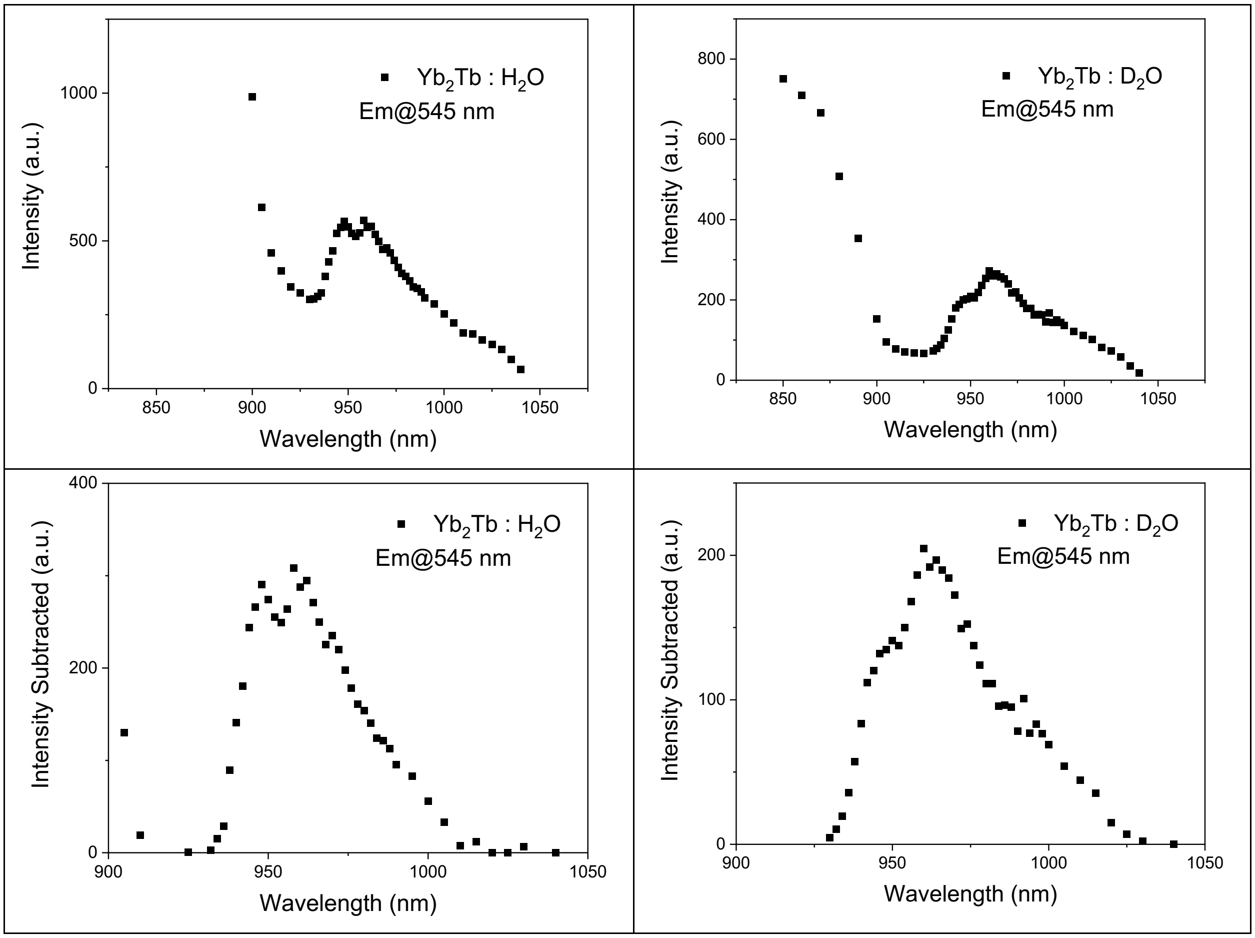

Figure S38. (top) Upconversion excitation spectra of Yb_2Tb in H_2O (left) and D_2O (right). (bottom) Upconversion excitation spectra of Yb_2Tb in H_2O (left) D_2O (right) with ligand centered signal has been subtracted. Emission measured at 545 nm. Laser power was kept constant at 550 ± 10 mW.

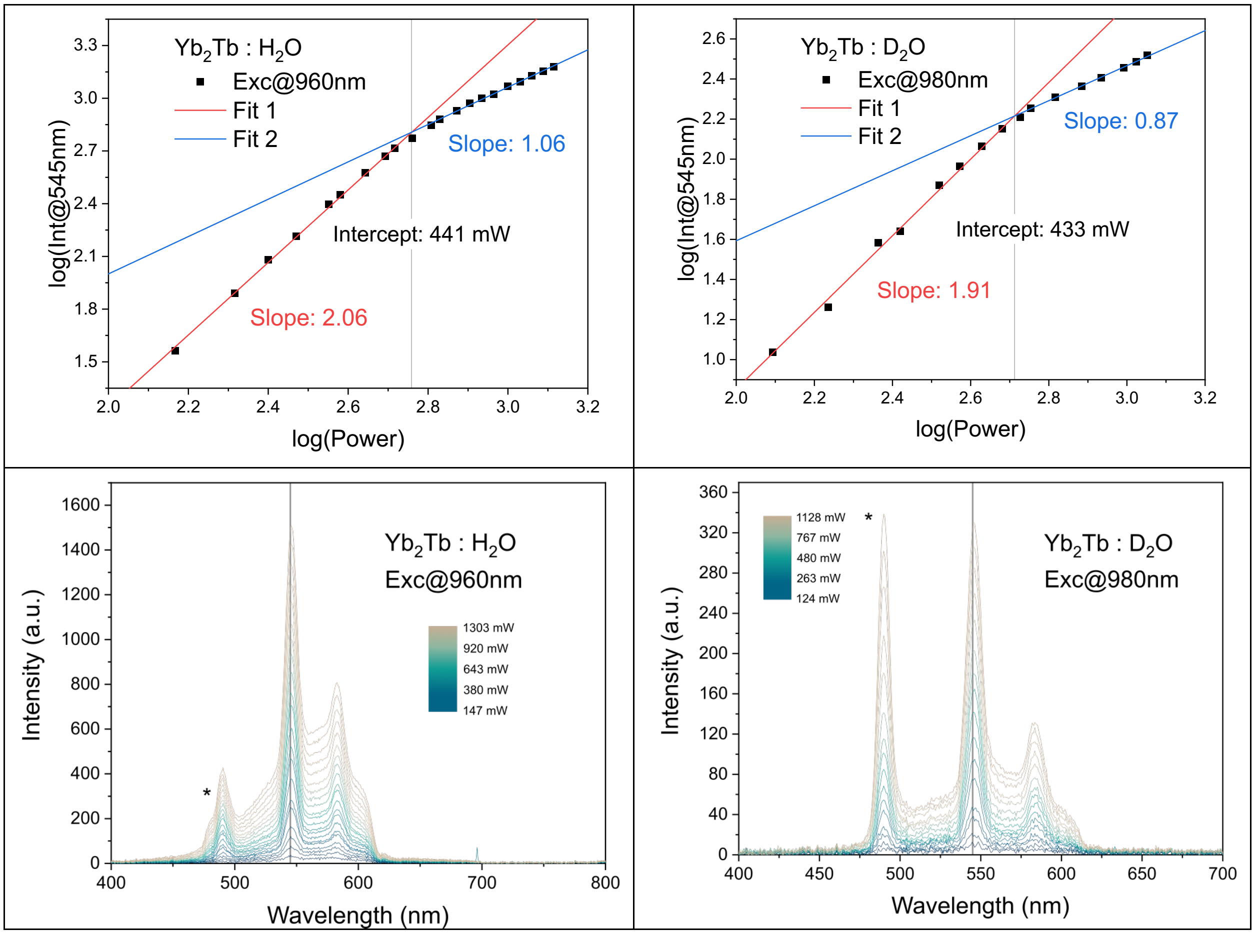

Figure S39. Top: Power dependence of upconversion emission at 545 nm for Yb_2Tb in H_2O (left) and D_2O (right), excited at 980 nm (laser beam radius = 11.8 ± 0.9 µm) and 960 nm (laser beam radius = 11.5 ± 0.9 µm). The data has been fitted with two linear functions. Vertical line indicates intercept between Fit 1 and Fit 2. Break point was determined using a Chow Test. Bottom: Upconversion emission spectra for Yb_2Tb in H_2O (left) and D_2O (right) excited at 960 nm and 980 nm. Laser signal is denoted with *. The vertical line indicates wavelength (545 nm) used for power dependence determination. Laser power range was 147-1303 mW and 124-1128 mW for samples in H_2O and D_2O respectively.

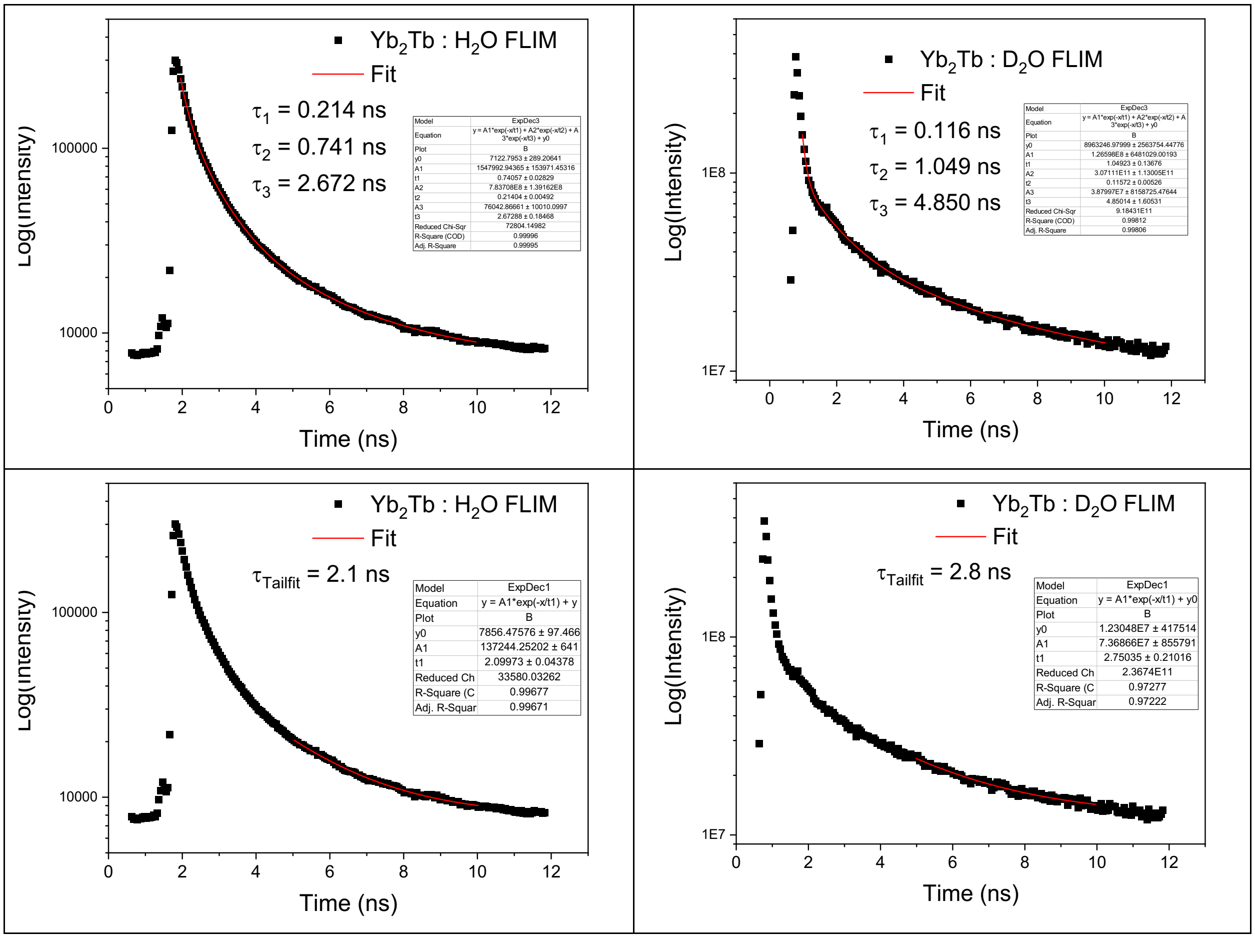

Figure S40. Time-resolved fluorescence decay of Yb_2Eu in H_2O (left) and D_2O (right) excited at 960 nm. Top: Data was fitted with a tri-exponential decay function. Bottom: Data was fitted with a mono-exponential decay function in the tail-end of the emission decay profile (5-10 ns)

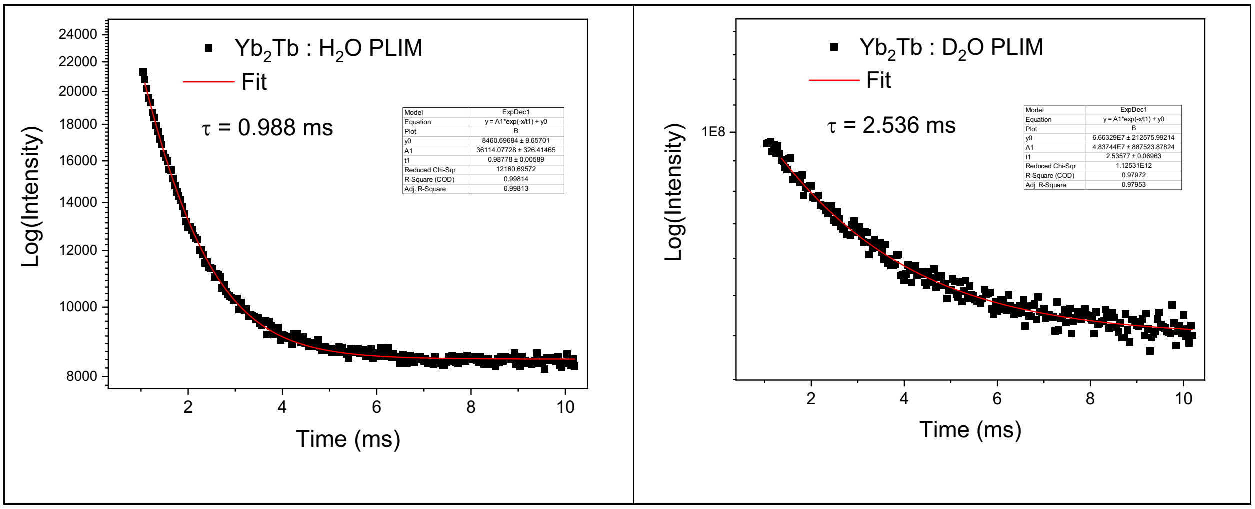

Figure S41. Time-resolved emission decay of long-lived emission of Yb_2Eu in H_2O (left) and D_2O (right) excited at 960 nm. Data was fitted with a mono-exponential decay function.

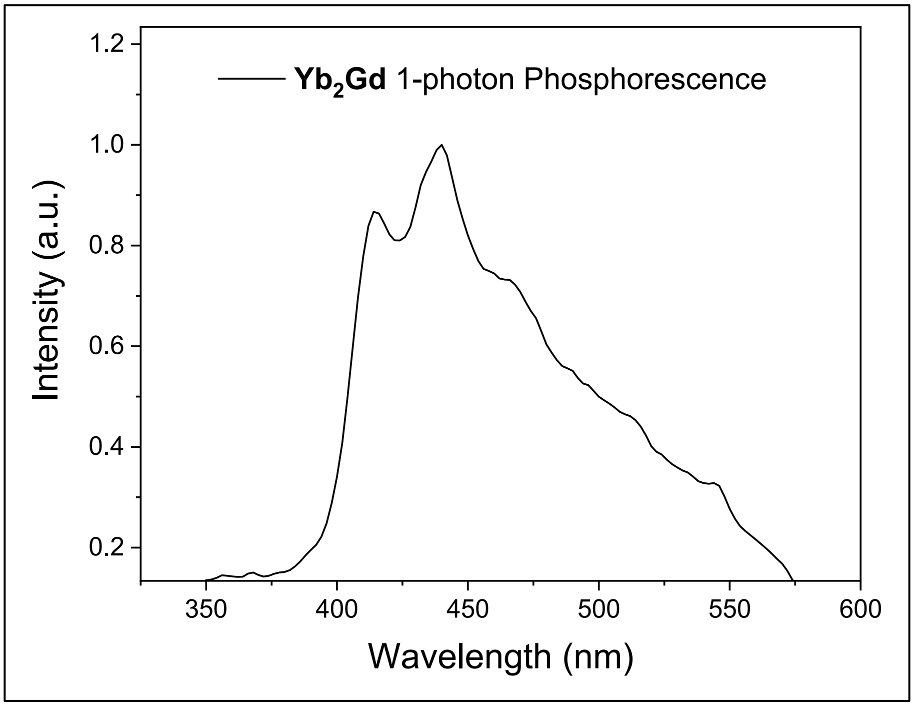

Figure S42. Corrected 1-photon phosphorescence of Yb_2Gd in D_2O at 77 K in frozen solution excited at 260 nm. No attempts were made to remove oxygen from the sample.

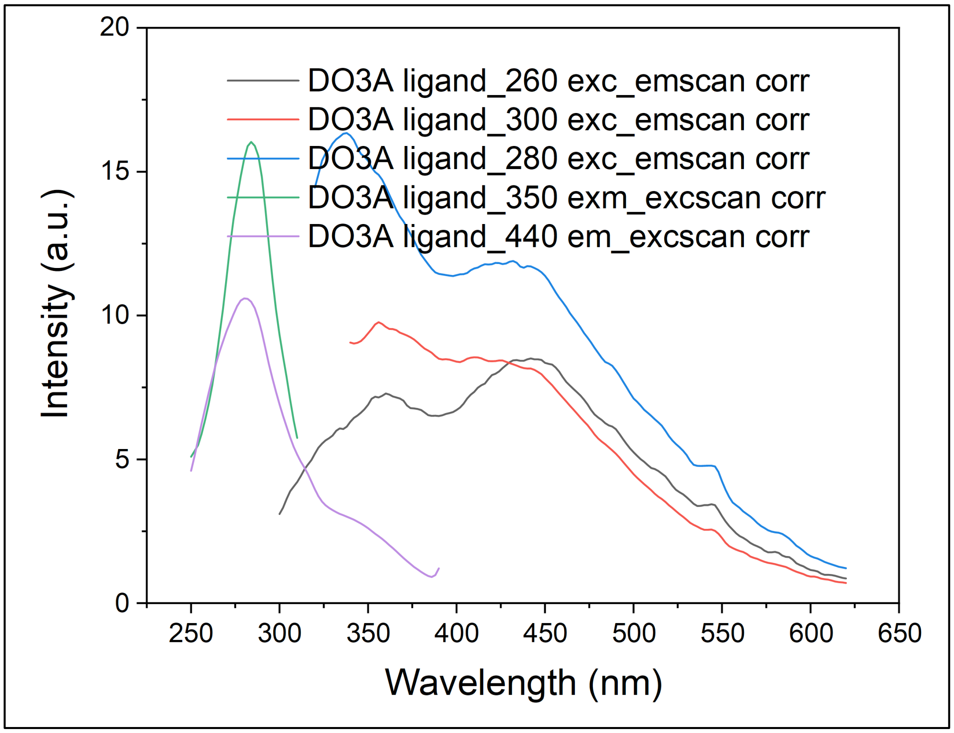

Figure S43. Corrected 1-photon fluorescence and excitation spectra of 2,2',2''-(10-(2-((4-aminophenyl)amino)-2-oxoethyl)-1,4,7,10-tetraazacyclododecane-1,4,7- triyl)triacetic acid (DO3A- aminophenyl acetamide) in D_2O at room temperature excited at 260 nm, 280 nm and 300 nm and the excitation region scanned monitoring the 350 and 440 nm emission bands.

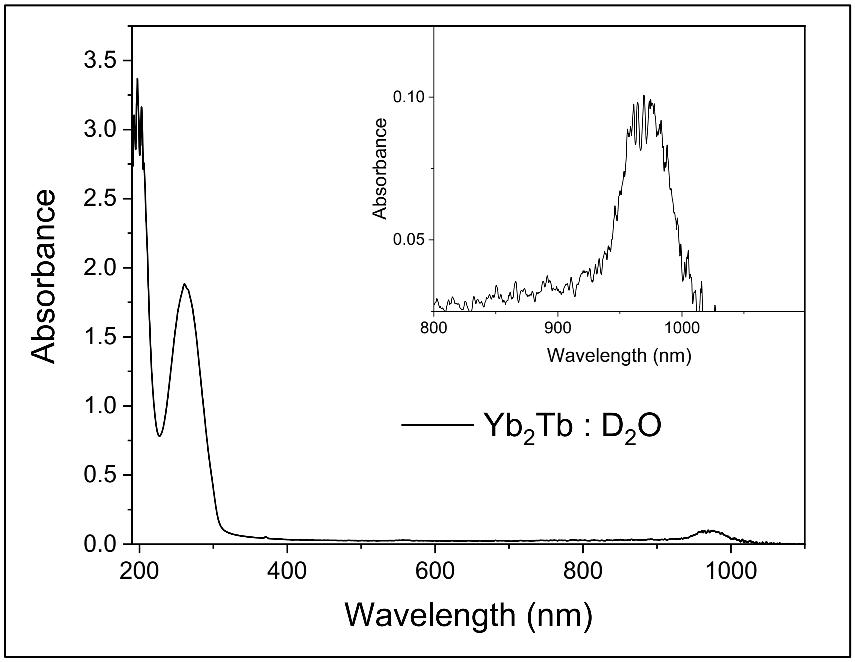

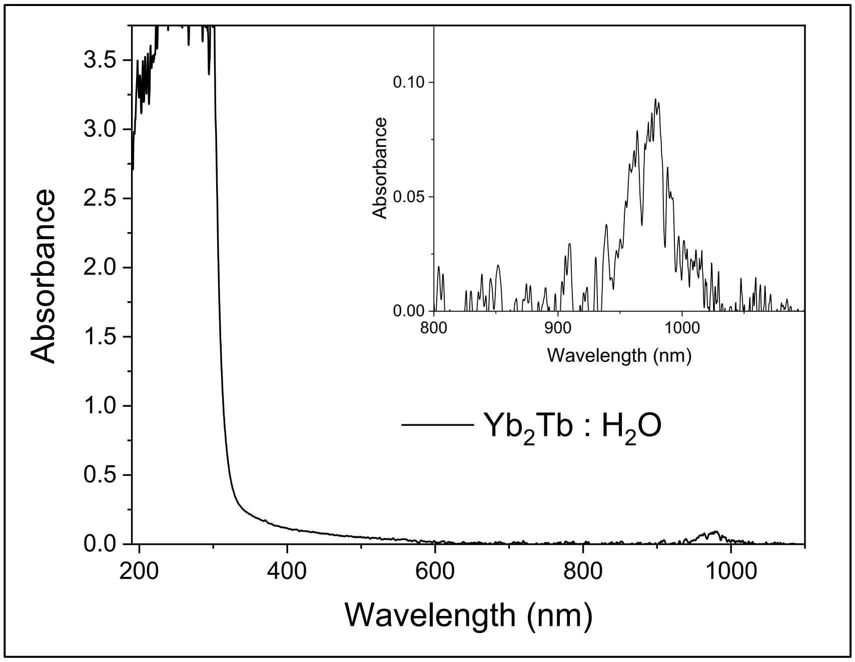

Figure S44. Absorption spectrum of Yb_2Tb in D_2O (top, 1.5 mM) and H_2O (bottom, 3 mM). Inset shows the Yb^{3+} $^2F_{5/2} \leftarrow {}^2F_{7/2}$ absorption band at ~980 nm.

Molecular Dynamic Simulations

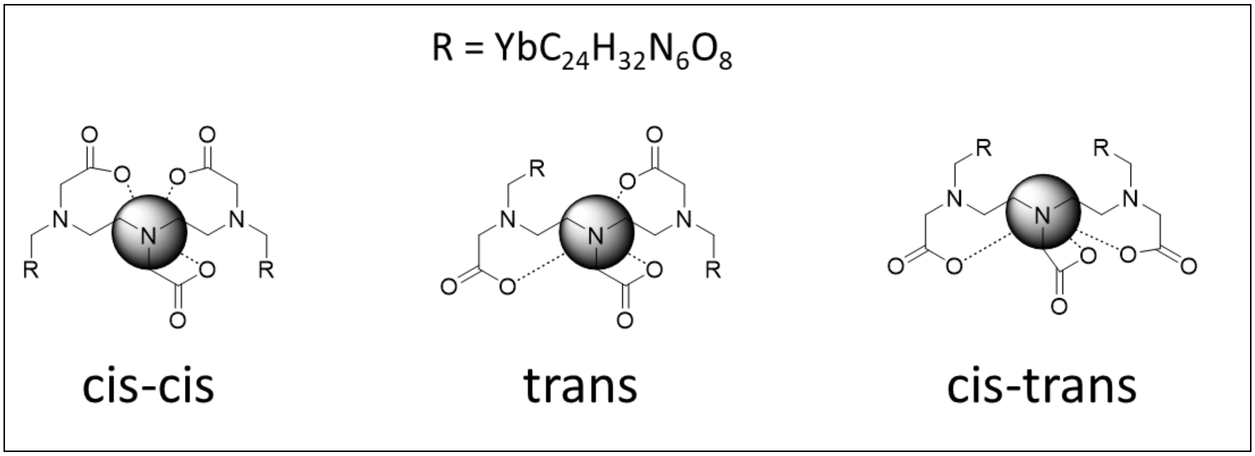

Figure S45. Illustration of the 3 main conformers of the Yb_2Tb complex with the Yb-arms cis towards the last DTPA carbonyl (cis-cis), trans, or cis away from the last carbonyl (cis-trans). Tb^{3+} is denoted as the grey ball. The two cis and the trans conformations give distinctly different intermetallic distances. Symmetry at the Yb-DOTA pocket is ignored to limit the computational cost.

Table S1. xyz coordinates for the three Yb_2Tb conformations with DFT, using the PBE functional.

Yb_2Tb(trans)				Yb_2Tb(cis-cis)				Yb_2Tb(cis-trans)			
Charge:			0	Charge:			0	Charge:			0
Spin: 1				Spin: 1				Spin: 1			
Tb	8.35915	6.73031	10.4602	Tb	7.49046	8.3102	13.52534	Tb	7.81525	7.75338	11.82205
N	10.12998	4.9957	9.39791	N	9.96195	7.274	12.89081	N	9.55921	6.0104	13.01476
N	9.91164	8.00368	8.79343	N	7.96097	8.51809	10.93837	N	9.3705	6.54823	10.05416
N	9.09656	9.07661	11.45866	N	6.67553	10.68918	12.5375	N	8.97579	9.55038	10.1898
C	8.1623	3.53168	9.90392	C	9.50951	6.04746	14.9457	C	7.84437	6.37076	14.7934
C	9.27993	3.89113	8.90137	C	10.05713	5.95473	13.52907	C	8.69241	5.34245	14.01051
H	9.86104	2.97705	8.65046	H	11.08004	5.52461	13.49538	H	9.27559	4.71295	14.71838
H	8.77199	4.23699	7.97932	H	9.37802	5.25298	12.99962	H	7.98361	4.69856	13.45222
C	11.18248	5.49836	11.57431	C	10.51719	9.65217	13.51272	C	10.32431	8.16472	13.93829
C	11.03274	4.49282	10.45197	C	10.9942	8.19349	13.4243	C	10.66342	6.70561	13.70528
H	12.02498	4.18422	10.05312	H	11.93931	8.13208	12.84204	H	11.55527	6.74533	13.04511
H	10.58372	3.59189	10.91941	H	11.23457	7.89544	14.46617	H	10.97695	6.17138	14.62445
C	10.86603	5.70487	8.3182	C	10.04973	7.17042	11.4159	C	10.08717	5.08206	11.98532
H	10.26595	5.62752	7.39182	H	9.54798	6.2368	11.09928	H	9.30058	4.33995	11.75844
H	11.83777	5.205	8.10509	H	11.1114	7.08481	11.08766	H	10.96494	4.50958	12.36276
C	11.14543	7.17497	8.63934	C	9.42797	8.37142	10.71052	C	10.49786	5.84473	10.7293
H	11.73281	7.2616	9.57342	H	9.9196	9.30577	11.04525	H	11.25715	6.60818	10.99309
H	11.77342	7.59451	7.81928	H	9.62656	8.27594	9.61723	H	10.98515	5.13984	10.01633
C	9.18549	8.09304	7.4964	C	7.22245	7.38929	10.31317	C	8.48165	5.56566	9.38252
H	9.88732	8.18972	6.63938	H	7.617	7.14863	9.30104	H	9.06489	4.77346	8.86288
H	8.5648	9.01192	7.52006	H	6.16636	7.70653	10.19461	H	7.89121	6.10773	8.61675
C	8.19437	6.94119	7.23526	C	7.16872	6.10297	11.16634	C	7.43981	4.91116	10.31358
C	10.277	9.37175	9.28005	C	7.51754	9.82707	10.3658	C	9.92767	7.53223	9.07734
H	9.5023	10.06731	8.90538	H	6.48499	9.69493	9.9906	H	9.1998	7.65075	8.25242
H	11.23984	9.7065	8.83389	H	8.13268	10.09989	9.4802	H	10.86084	7.14379	8.61245
C	10.36669	9.4775	10.7995	C	7.55968	10.96958	11.37775	C	10.21733	8.88988	9.71477
H	11.15067	8.80122	11.19139	H	8.58188	11.09279	11.78432	H	10.88146	8.76821	10.59448
H	10.66148	10.5182	11.07052	H	7.28022	11.91909	10.86499	H	10.75236	9.52818	8.9733
C	8.00345	10.0608	11.25141	C	5.24434	10.70203	12.16722	C	8.06185	9.88175	9.06629
H	8.14513	10.58359	10.28502	H	5.12704	10.57737	11.07064	H	8.62859	10.14723	8.14745
H	7.98643	10.82381	12.05836	H	4.7661	11.66822	12.41827	H	7.47398	10.78013	9.34774
C	6.68403	9.31623	11.12171	C	4.52353	9.49885	12.74899	C	7.01544	8.79832	8.75446
C	9.28005	8.86722	12.91362	C	6.89169	11.62616	13.66335	C	9.26711	10.76686	10.96507
H	9.29681	9.82599	13.46699	H	6.4507	12.6272	13.46363	H	9.54434	11.63349	10.32329
H	10.25016	8.35982	13.06801	H	7.98595	11.72649	13.80374	H	10.11425	10.56508	11.65038
C	8.22848	7.93214	13.52822	C	6.32525	11.05397	14.9799	C	8.06469	11.08289	11.85051
O	7.80146	4.50308	10.72113	O	8.68319	6.9545	15.24331	O	7.33041	7.32015	14.06317
O	7.65146	2.40587	9.87171	N	9.84669	5.11603	15.86309	O	7.68021	6.23603	16.02208

O	10.3868	6.47079	11.65184	O	9.24104	9.78627	13.75416	O	9.69199	8.79222	13.04764
N	12.13157	5.29689	12.51097	O	11.33066	10.5841	13.40515	N	10.77549	8.80834	15.03558
O	7.63652	6.43785	8.30521	O	7.17178	6.2869	12.45924	O	7.07111	5.62751	11.34228
O	7.95108	6.5981	6.06682	O	7.08053	5.00237	10.59511	O	6.98814	3.792	10.01668
O	6.59771	8.38178	10.26863	O	5.12638	8.39535	12.80591	O	6.64917	8.08001	9.78088
N	5.68001	9.69608	11.93386	N	3.22045	9.58057	13.10416	O	6.5607	8.69974	7.60234
O	7.66787	7.08134	12.70055	O	6.53878	9.77499	15.14888	O	7.31108	10.16244	12.26032
O	8.01187	7.96994	14.75383	O	5.74105	11.79863	15.78873	N	7.89639	12.38483	12.1823
O	8.30644	4.4889	13.52413	O	5.98721	7.19566	15.28611	O	5.32238	8.08105	12.06011
H	8.18855	4.21915	12.58111	H	5.81834	8.08305	15.69433	H	5.16557	8.96227	12.4606
H	7.97955	5.42238	13.45868	Yb	16.75458	-1.11766	12.95178	H	5.22666	8.25122	11.08945
Yb	-3.7534	7.92108	10.85132	N	17.32162	-2.61589	10.96683	Yb	17.18499	4.70025	21.04444
N	-4.28259	9.26224	8.73604	C	17.81794	-1.78746	9.83233	N	18.34426	2.45189	21.40096
N	-1.83043	9.6365	10.39266	H	18.34596	-2.42928	9.09026	C	19.38887	2.24476	20.35904
N	-3.49518	9.53946	12.86002	H	16.93976	-1.35838	9.3128	H	20.09653	1.44512	20.67729
N	-5.95626	9.16431	11.19779	C	18.75122	-0.66671	10.27871	H	18.88926	1.87884	19.44154
C	-3.4024	10.4576	8.63514	H	19.10434	-0.10995	9.38125	C	20.17612	3.51392	20.04816
C	-1.95384	10.16225	9.00217	H	19.65097	-1.08688	10.76545	H	20.95269	3.28111	19.28458
C	-1.909	10.76241	11.36685	N	18.08966	0.24819	11.25124	H	20.71207	3.86216	20.95046
C	-2.21889	10.30406	12.78714	C	17.20432	1.2	10.52713	N	19.28696	4.6183	19.58938
C	-4.64401	10.48811	12.85992	H	16.66353	0.63563	9.74325	C	18.92908	4.41665	18.15929
C	-5.97269	9.81614	12.53745	H	17.81316	1.97208	10.00249	H	18.69957	3.34378	18.01128
C	-6.13255	10.18812	10.13007	C	16.20657	1.89902	11.44174	H	19.8012	4.65021	17.50578
C	-5.71032	9.68717	8.75289	H	15.60513	2.61839	10.84163	C	17.74268	5.26457	17.7187
C	-4.01764	8.31878	7.61923	H	16.74011	2.49388	12.20628	H	17.55458	5.09264	16.63504
C	-2.8501	7.3428	7.87173	N	15.32357	0.92566	12.14857	H	17.97722	6.33934	17.83195
O	-2.23693	6.87299	6.89921	C	14.24506	0.46031	11.23026	N	16.5219	4.97049	18.52445
O	-2.62466	7.03916	9.12462	H	14.70013	0.3008	10.2344	C	15.87912	3.71968	18.02837
C	-0.55116	8.90434	10.53541	C	13.57262	-0.82347	11.70172	H	16.68707	2.99882	17.80098
C	-0.63187	7.85884	11.63488	H	12.77091	-1.09534	10.97808	C	14.90969	3.10523	19.03117
N	0.47006	7.53919	12.3478	H	13.06791	-0.66096	12.67287	H	14.46532	2.18748	18.58314
O	-1.72714	7.28001	11.88294	H	13.47296	1.25356	11.10493	H	14.06467	3.79285	19.22371
C	-3.52834	8.67938	14.07173	C	14.74989	1.56345	13.35524	H	15.34322	3.91251	17.07105
C	-4.37899	7.40085	13.93108	H	15.5265	2.21902	13.79814	C	15.58464	6.11376	18.44198
O	-4.8487	6.87751	14.95477	H	13.87137	2.19829	13.10761	H	16.17967	7.04894	18.46164
O	-4.49604	6.92758	12.71786	C	14.4184	0.53965	14.42664	H	15.00172	6.1009	17.49569
C	-7.01303	8.12375	11.10604	N	13.42848	0.77124	15.32093	C	14.67955	6.17644	19.66029
C	-6.69314	6.97804	10.1268	H	13.23993	-0.02733	15.9402	N	13.43125	6.68614	19.55741
O	-7.62862	6.33269	9.6258	C	12.55746	1.88843	15.43895	H	12.87021	6.60144	20.41451
O	-5.42173	6.73698	9.93973	C	13.0332	3.21298	15.37718	C	12.78384	7.22042	18.40634
H	-3.85559	8.85624	6.6592	H	14.10601	3.41811	15.26931	C	13.39187	8.22323	17.62744

H	-4.91462	7.67797	7.50193	C	12.13832	4.28185	15.50943	H	14.37605	8.62282	17.90656
H	-3.43408	10.87506	7.60223	H	12.50998	5.31579	15.48613	C	12.72985	8.72431	16.49988
H	-3.80877	11.23904	9.30598	C	10.76854	4.03342	15.70775	H	13.20327	9.49614	15.87712
H	-1.53055	9.40842	8.31274	C	10.29935	2.7116	15.81378	C	11.44871	8.25043	16.16867
H	-1.34911	11.08786	8.87187	H	9.23003	2.52511	15.9854	C	10.81511	7.28606	16.97735
H	0.30493	9.59651	10.68696	C	11.19117	1.6425	15.68088	H	9.79654	6.94082	16.74059
H	-0.36923	8.34062	9.59842	H	10.82893	0.60663	15.74725	C	11.49147	6.76285	18.08509
H	-0.95774	11.34227	11.36759	C	19.09352	0.98084	12.06569	H	11.01486	5.99694	18.71342
H	-2.70099	11.45066	11.01562	H	19.49302	1.86604	11.52388	C	19.93188	5.94275	19.78687
H	-1.40787	9.65769	13.17292	H	19.93916	0.28995	12.25625	H	20.63216	6.1843	18.95745
H	-2.24592	11.19579	13.45385	C	18.59429	1.40805	13.46136	H	20.5215	5.8929	20.72429
H	-3.85652	9.24948	14.96834	O	19.11462	2.39258	14.01112	C	18.94467	7.11111	19.98925
H	-2.49574	8.32371	14.26477	C	18.38947	-3.52637	11.45603	O	19.30019	8.26016	19.67985
H	-4.72292	10.99473	13.84947	H	18.97881	-3.95343	10.61539	C	18.96267	2.54375	22.74908
H	-4.43404	11.27895	12.11388	C	19.33874	-2.89333	12.49132	H	19.78777	1.80836	22.87066
H	-6.20007	9.03781	13.2899	O	20.4861	-3.35133	12.61823	C	19.45818	3.95359	23.12409
H	-6.7872	10.57272	12.60458	H	17.89925	-4.36846	11.9853	O	20.37256	4.07222	23.95614
H	-8.00231	8.56699	10.8583	C	16.11921	-3.3898	10.54696	H	18.18067	2.30016	23.49653
H	-7.09997	7.64257	12.10127	H	16.26221	-3.83308	9.53539	C	17.34525	1.34693	21.35178
H	-7.19491	10.52157	10.0885	H	15.99859	-4.23597	11.24927	H	17.84622	0.35977	21.23035
H	-5.53104	11.0759	10.40577	C	14.8608	-2.53032	10.53745	H	16.82308	1.31915	22.32659
H	-6.32652	8.81885	8.45421	H	14.97871	-1.70166	9.8127	C	16.34006	1.53493	20.22173
H	-5.89984	10.48732	8.0018	H	14.00325	-3.14633	10.18082	H	16.86818	1.54094	19.24861
C	2.45842	8.04114	10.97542	O	18.82444	-1.93069	13.21182	H	15.64633	0.66284	20.20334
C	3.74847	8.57246	10.85818	N	14.54746	-1.93765	11.86822	O	18.81867	4.94261	22.55614
C	4.38826	9.1181	11.99094	C	14.01096	-2.97476	12.78821	N	15.56674	2.80381	20.33358
C	3.72253	9.12327	13.23715	C	15.08383	-3.75817	13.57101	C	14.55992	2.70715	21.42291
C	2.42944	8.60366	13.34555	O	14.81191	-4.89624	13.98686	C	15.0842	3.09393	22.82049
C	1.78768	8.07008	12.21189	H	13.34237	-3.68635	12.256	O	14.51575	2.63152	23.82319
H	1.97451	7.59234	10.09704	H	13.40124	-2.46277	13.56035	H	14.09814	1.69633	21.46396
H	4.25789	8.55832	9.88857	O	16.20592	-3.1223	13.7865	H	13.74911	3.43169	21.20427
H	4.2316	9.54638	14.11471	O	17.67034	0.64772	13.99191	O	16.07819	3.94313	22.83884
H	1.90563	8.61698	14.31203	O	15.06995	-0.53868	14.50162	O	17.79625	6.79736	20.53323
H	0.30017	6.88229	13.11996	Yb	-2.36857	16.90382	16.22664	O	15.08819	5.75782	20.77891
Yb	10.06779	12.54712	15.40224	N	-4.46023	17.28466	17.63885	H	10.6528	9.82893	15.01581
N	11.16127	14.75996	16.01819	C	-4.13593	17.11583	19.08305	Yb	3.39776	19.30053	14.93753
N	11.10403	12.36019	17.8164	H	-4.93359	17.57282	19.71252	N	2.0093	20.90511	13.52958
N	8.21608	11.9862	17.10074	H	-4.13535	16.03196	19.30838	C	0.87906	21.43897	14.34073
N	8.28911	14.40228	15.34929	C	-2.7892	17.72245	19.46382	H	0.4523	22.34677	13.85567
C	11.4047	14.82565	17.48536	H	-2.62561	17.58544	20.55688	H	0.07587	20.67707	14.35065
C	11.99172	13.53918	18.04927	H	-2.79302	18.81234	19.27663	C	1.28353	21.78047	15.77153

C	9.99654	12.34445	18.81433	N	-1.6732	17.12731	18.67583	H	0.40193	22.20076	16.3066
C	8.8153	11.48999	18.37105	C	-1.31083	15.79897	19.23889	H	2.05975	22.56816	15.77245
C	7.40078	13.20789	17.35792	H	-2.24888	15.26023	19.47425	N	1.83317	20.59565	16.48975
C	7.06312	13.9771	16.08463	H	-0.76251	15.92298	20.20125	C	0.7198	19.72813	16.9615
C	8.83048	15.64777	15.96164	C	-0.46006	14.96262	18.29184	H	-0.03566	19.67348	16.1541
C	10.28992	15.8955	15.60211	H	-0.19305	14.00478	18.79226	H	0.21521	20.19168	17.84044
C	12.44688	14.78345	15.27105	H	0.49203	15.48126	18.07428	C	1.17469	18.32342	17.3399
C	13.10073	13.40104	15.07698	N	-1.15657	14.71161	16.99631	H	0.29926	17.74786	17.71637
O	14.3275	13.33173	14.90076	C	-2.14744	13.60924	17.16019	H	1.9056	18.36625	18.16859
O	12.27793	12.38277	15.06258	H	-2.6425	13.74852	18.13997	N	1.82028	17.62086	16.19398
C	11.89674	11.11258	17.88227	C	-3.19427	13.57828	16.05277	C	0.78058	17.09949	15.26212
C	11.54047	10.11165	16.79141	H	-3.88585	12.7247	16.23606	H	-6.09E-04	17.87742	15.16769
N	12.32822	9.01398	16.75207	H	-2.71573	13.38239	15.07457	C	1.33031	16.75455	13.88227
O	10.59241	10.30984	15.98462	H	-1.62854	12.62439	17.20135	H	0.49706	16.36918	13.25178
C	7.37904	10.93391	16.47102	C	-0.16072	14.37351	15.9544	H	2.06527	15.93082	13.95083
C	7.24477	11.12154	14.95697	H	0.73866	14.99937	16.12245	H	0.2867	16.19964	15.69535
O	6.1718	10.79993	14.38735	H	0.1505	13.30805	16.0135	C	2.68394	16.52687	16.69185
O	8.29009	11.58396	14.35262	C	-0.65829	14.73648	14.56615	H	3.21723	16.8963	17.59156
C	8.00147	14.60725	13.90448	N	-0.2331	14.04696	13.48212	H	2.09571	15.62817	16.98183
C	9.19316	14.34978	12.96131	H	-0.69686	14.32318	12.6073	C	3.77531	16.19818	15.68583
O	9.16326	14.83267	11.81831	C	0.65992	12.94084	13.41314	N	4.13607	14.90042	15.57217
O	10.14233	13.57975	13.43044	C	1.90056	12.94723	14.07845	H	3.59572	14.23881	16.13864
H	13.17571	15.48187	15.73701	H	2.21699	13.82202	14.6615	C	5.10183	14.31305	14.71999
H	12.23409	15.15367	14.24812	C	2.7481	11.8351	13.99602	C	5.97605	15.05513	13.89504
H	12.09494	15.66772	17.72339	H	3.69179	11.83274	14.5618	H	5.94093	16.14868	13.89208
H	10.44294	15.0521	17.98398	C	2.38539	10.73613	13.19452	C	6.88319	14.38265	13.07178
H	12.96678	13.32968	17.5718	C	1.15626	10.73975	12.50772	H	7.55897	14.96805	12.43029
H	12.19066	13.67773	19.13564	H	0.87792	9.87681	11.8865	C	6.94381	12.97334	13.04518
H	11.7971	10.61363	18.87235	C	0.29255	11.83344	12.6235	C	6.07376	12.23182	13.87503
H	12.971	11.35201	17.76122	H	-0.67441	11.83516	12.10048	H	6.10473	11.1378	13.87197
H	10.36621	11.97597	19.79807	C	-0.49226	18.02898	18.65395	C	5.16855	12.90386	14.70098
H	9.66988	13.38966	18.97186	H	0.12351	17.9193	19.57352	H	4.49375	12.31892	15.34385
H	9.14038	10.44617	18.19949	H	-0.86692	19.07167	18.61995	C	2.69373	21.01087	17.62804
H	8.05846	11.46488	19.1876	C	0.40411	17.87815	17.4074	H	2.08986	21.25431	18.52954
H	6.37812	10.86134	16.94815	O	1.60396	18.1883	17.48708	H	3.23365	21.92978	17.32308
H	7.89089	9.95662	16.58667	C	-4.87313	18.68355	17.35444	C	3.7958	19.99767	17.99917
H	6.45558	12.93513	17.88113	H	-5.51714	19.0919	18.16381	O	4.24073	19.98995	19.15843
H	7.97015	13.86037	18.04716	C	-3.70359	19.6487	17.08053	C	2.93951	21.9977	13.14269
H	6.45274	13.35245	15.4066	O	-3.86491	20.86395	17.27932	H	2.38966	22.92387	12.86648
H	6.43208	14.85459	16.351	H	-5.47085	18.67592	16.42057	C	4.01357	22.32062	14.1986
H	7.58795	15.62113	13.71148	C	-5.52954	16.32635	17.23994	O	4.5258	23.45171	14.2144

H	7.23171	13.86931	13.60205	H	-6.33506	16.28318	18.00772	H	3.50121	21.66112	12.24779
H	8.22169	16.52656	15.64854	H	-5.99648	16.70663	16.31196	C	1.48883	20.20619	12.32059
H	8.72192	15.56242	17.06022	C	-4.98252	14.92168	17.01647	H	0.67776	20.7958	11.83596
H	10.39903	16.02042	14.50874	H	-4.5351	14.54036	17.95475	H	2.3153	20.14006	11.58819
H	10.62558	16.84783	16.07175	H	-5.82668	14.23905	16.76423	C	0.96318	18.81367	12.64467
C	11.11705	7.2695	15.50217	O	-2.62126	19.09784	16.59604	H	0.12389	18.88611	13.3633
C	11.09072	6.32651	14.46864	N	-3.94105	14.86335	15.9524	H	0.54909	18.3575	11.71593
C	12.22852	6.14132	13.66188	C	-4.56004	15.01	14.60883	O	4.34577	21.32805	14.98281
C	13.42159	6.82995	13.93752	C	-4.72767	16.46657	14.13198	N	1.99806	17.91968	13.2368
C	13.45317	7.75898	14.98682	O	-5.60388	16.72497	13.29072	C	2.96023	17.4693	12.19643
C	12.29048	8.00552	15.7409	H	-5.53755	14.48306	14.55048	C	4.17388	18.39623	11.98242
H	10.21479	7.45119	16.09912	H	-3.88465	14.53602	13.86775	O	4.79794	18.31673	10.91179
H	10.18093	5.74287	14.25916	O	-3.87493	17.32675	14.62431	H	2.45391	17.29084	11.22249
H	14.30878	6.66988	13.30869	O	-0.19955	17.47095	16.31983	H	3.3912	16.50079	12.52232
H	14.37025	8.32796	15.19633	O	-1.4669	15.69324	14.40915	O	4.49095	19.16994	12.98739
H	13.13961	9.00536	17.3796	H	6.66131	6.80417	15.88433	O	4.22266	19.23886	17.02163
H	12.84937	4.58432	12.34353	H	9.2972	5.14928	16.73101	O	4.31797	17.1235	15.02332
H	5.94354	10.30465	12.75695	H	2.77854	8.67224	13.29497	H	8.53231	13.04346	11.72176

Table S2. Energies of Yb_2Tb conformations from DFT calculations, using PBE functional.

	Yb_2Tb(trans)	Yb_2Tb(cis-cis)	Yb_2Tb(cis-trans)
Energy (Hartree)	-4921.304436998755	-4921.273268420408	-4921.285888853934

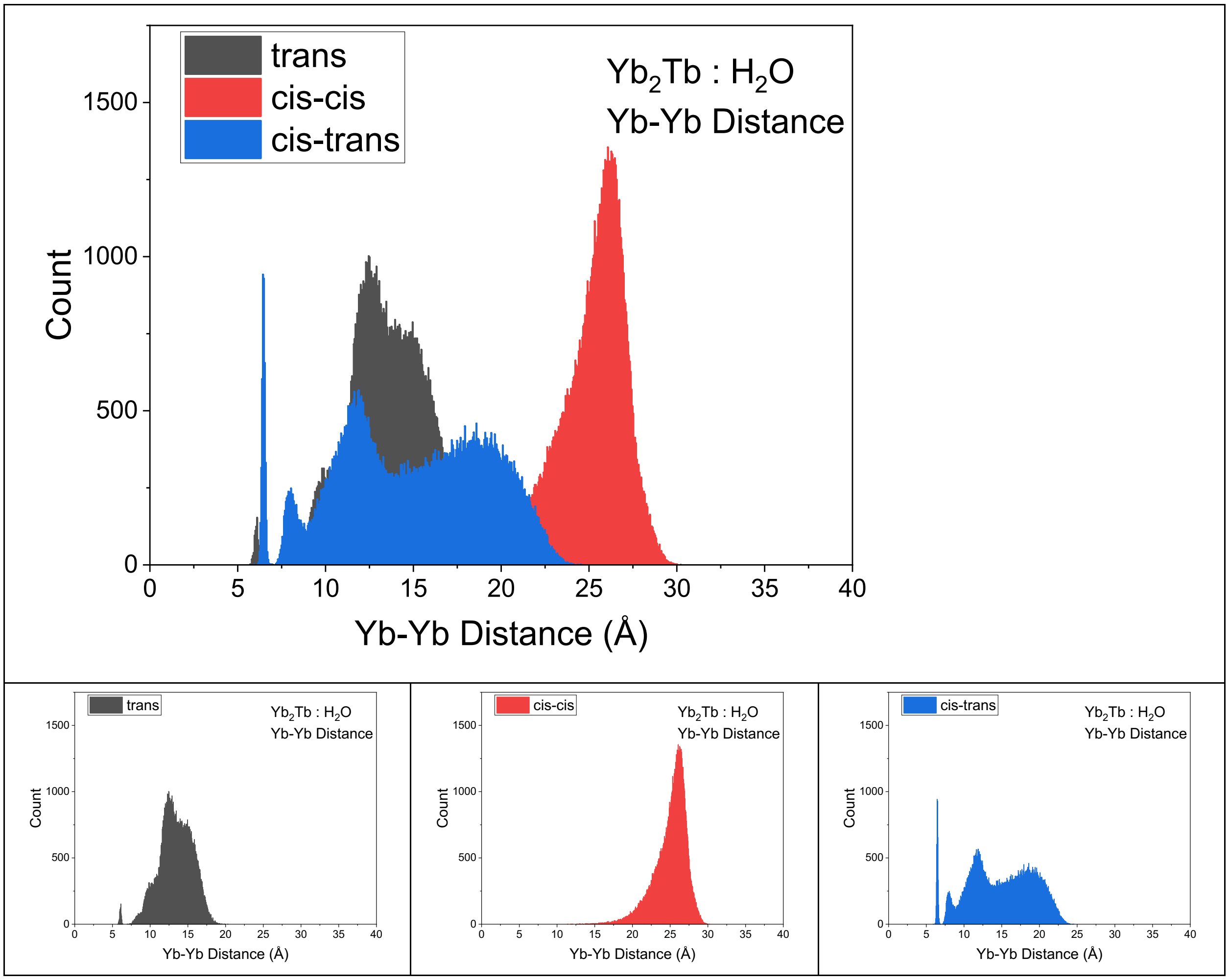

Figure S46. Histograms of the Yb-Yb distances in the three Yb_2Tb conformations observed in MD simulations (see Figure S45). The *trans* (grey), *cis-cis* (red) and *cis-trans* (blue) conformations are shown individually.

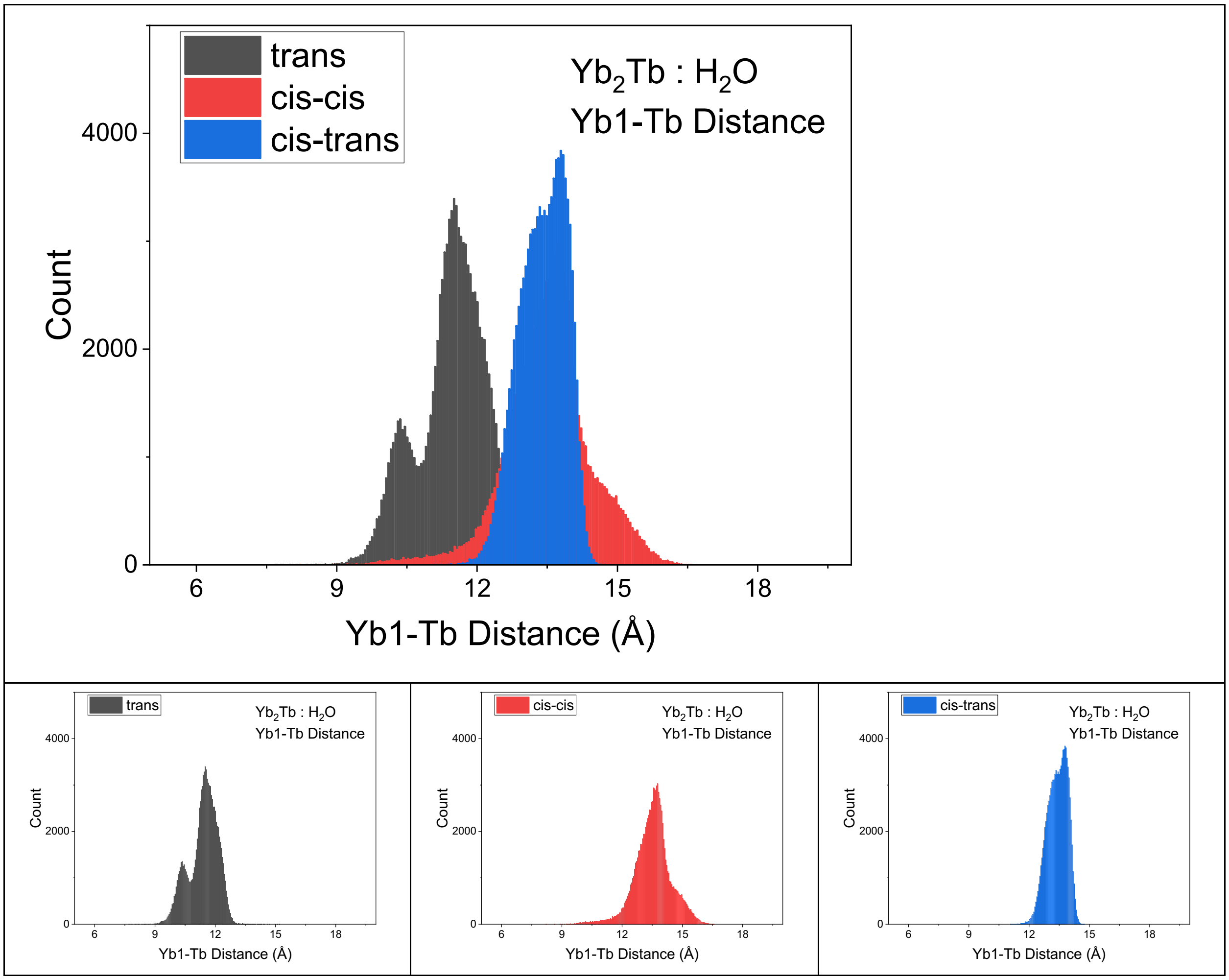

Figure S47. Histograms of the Yb1-Tb distances in the three Yb_2Tb conformations observed in MD simulations (see Figure S45). The *trans* (grey), *cis-cis* (red) and *cis-trans* (blue) conformations are shown individually.

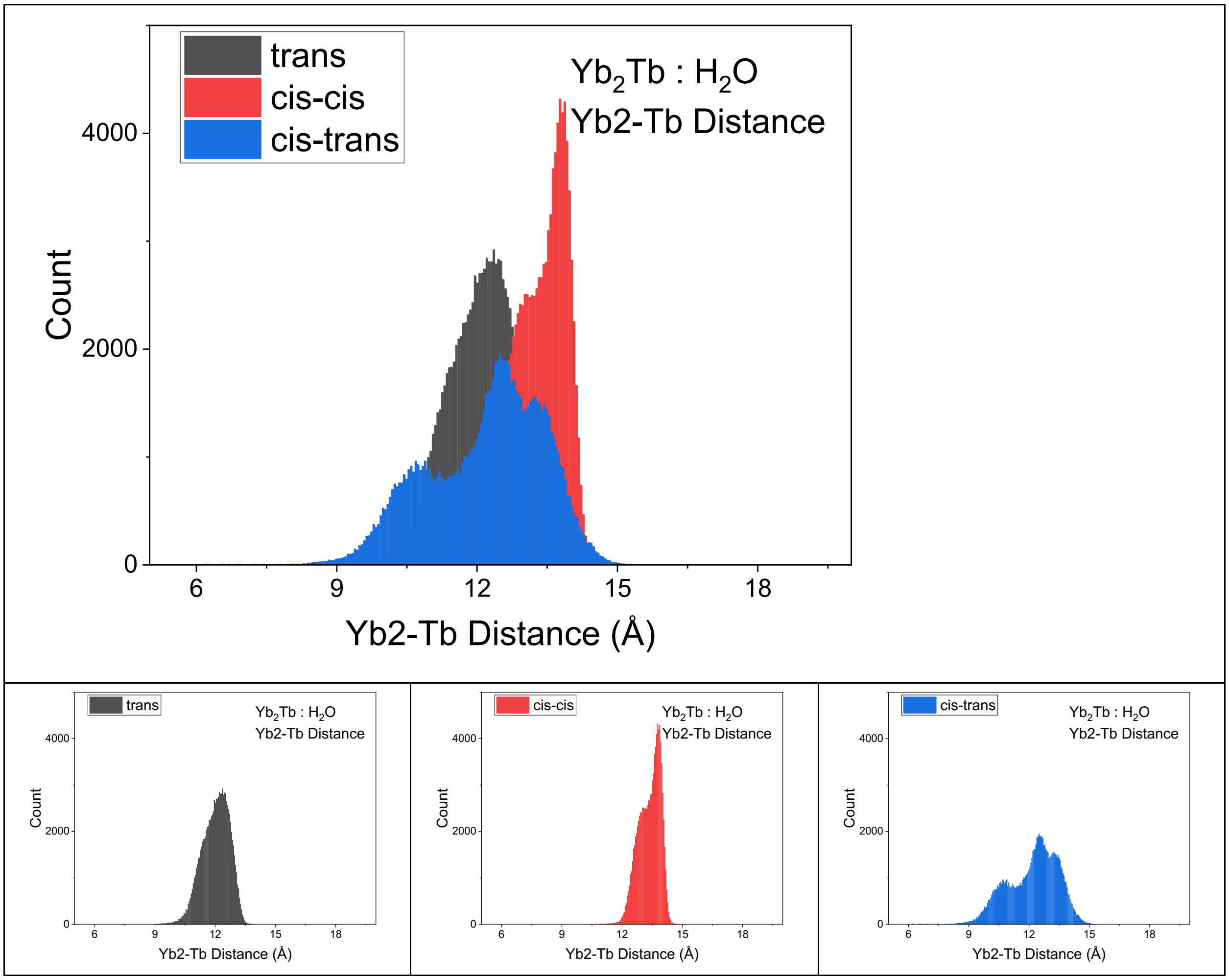

Figure S48. Histograms of the Yb2-Tb distances in the three Yb_2Tb conformations observed in MD simulations (see Figure S45). The *trans* (grey), *cis-cis* (red) and *cis-trans* (blue) conformations are shown individually.

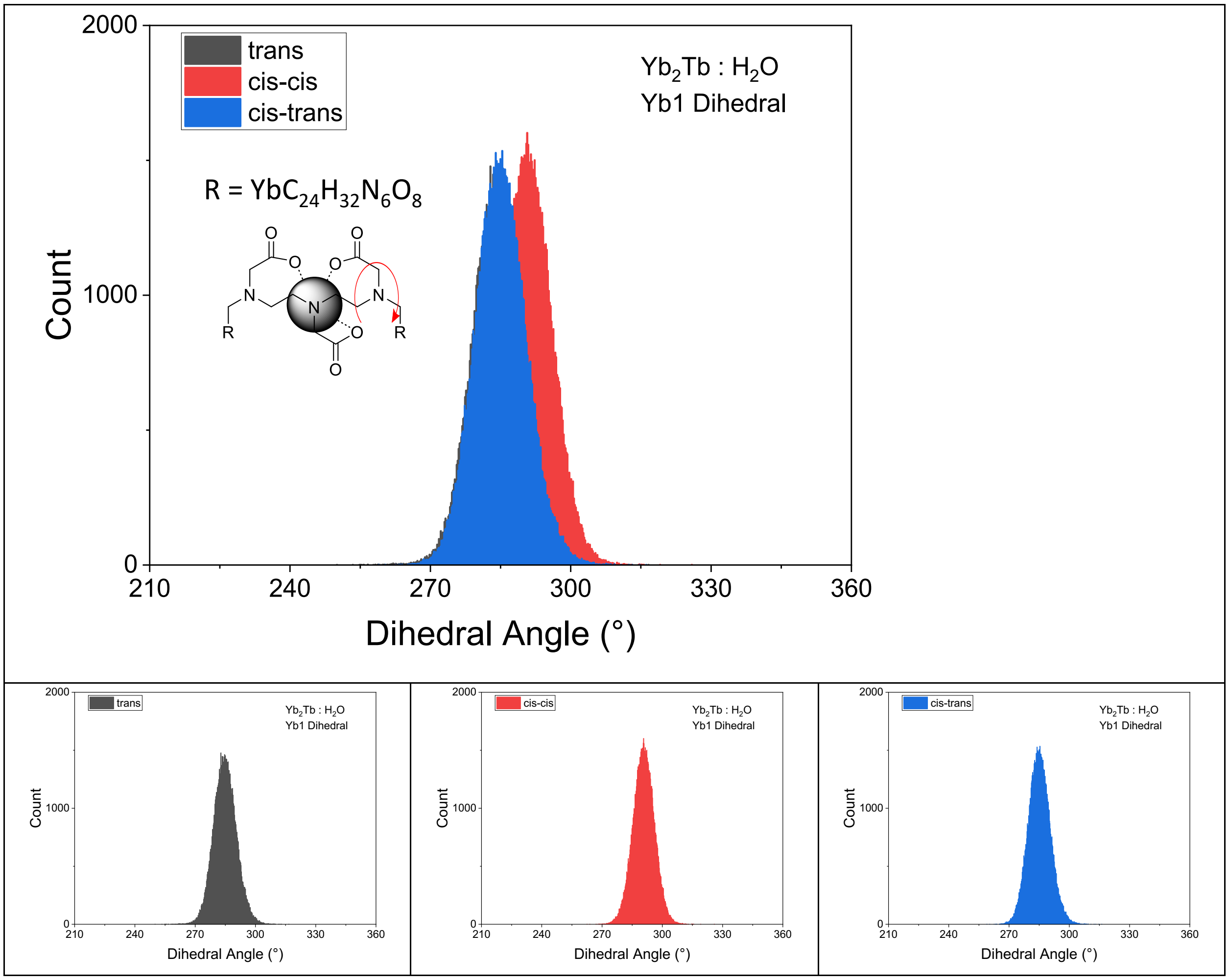

Figure S49. Histograms of the dihedral angle around the carbonyl/YbDOTA arm of Yb1 in the three Yb_2Tb conformations observed in MD simulations (see Figure S45). The *trans* (grey), *cis-cis* (red) and *cis-trans* (blue) conformations are shown individually.

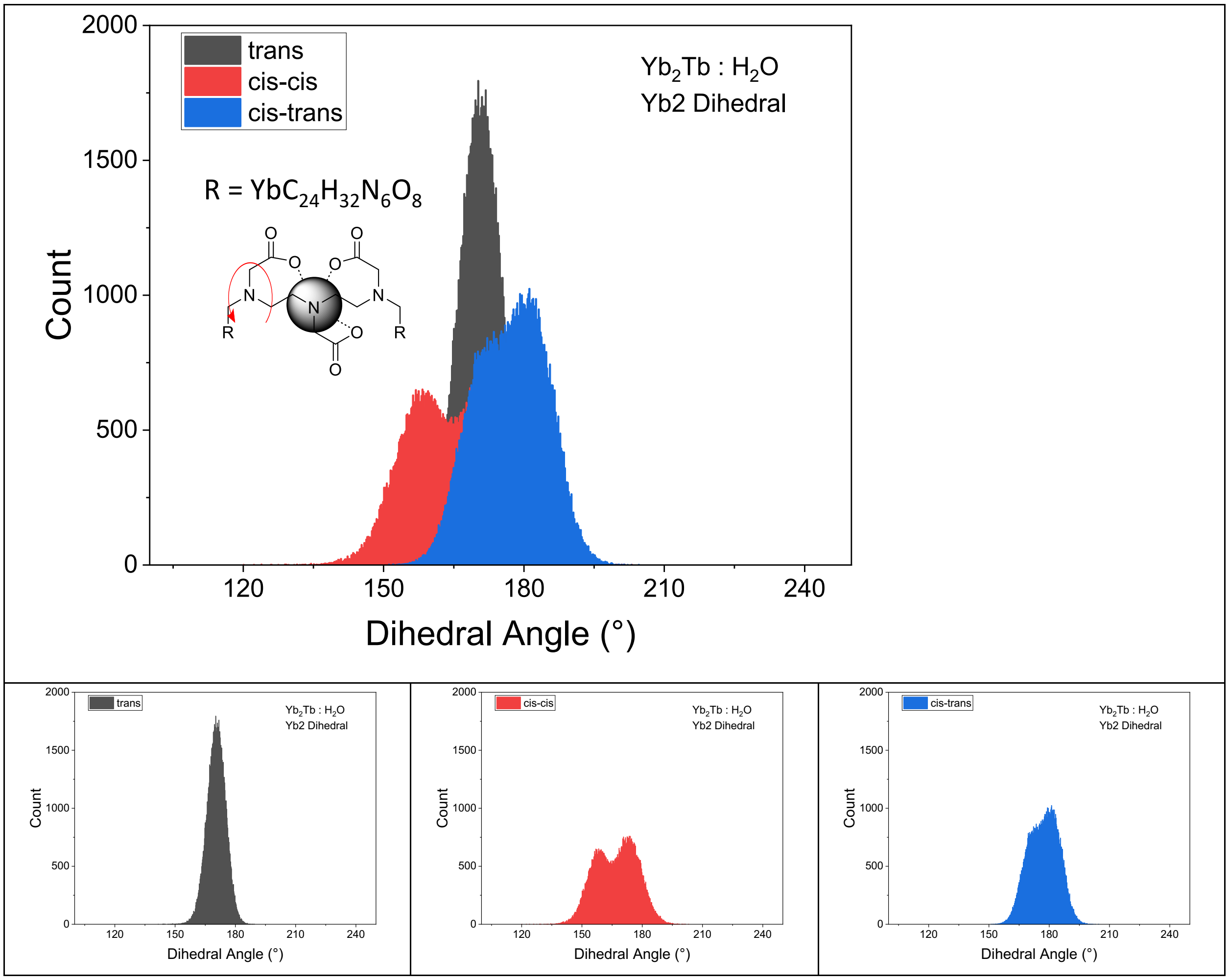

Figure S50. Histograms of the dihedral angle around the carbonyl/YbDOTA arm of Yb_2 in the three Yb_2Tb conformations observed in MD simulations (see Figure S45). The trans (grey), cis-cis (red) and cis-trans (blue) conformations are shown individually.

Table S3. Number of directly coordinated waters to each metal centre in Yb_2Tb trans conformation calculated with a cut-off of 2.7 Å between the metal centre and the oxygen atom of solvent waters during a 1 µs MD simulation.

Yb_2Tb - trans			
$n_{(H_2O)}$	Tb	Yb1	Yb2
0	0.34 %	99.97 %	99.98 %
1	98.92 %	0.03 %	0.02 %
2	0.72 %	-	-
3	0.01 %	-	-

Table S4. Number of directly coordinated waters to each metal centre in Yb_2Tb cis-cis conformation calculated with a cut-off of 2.7 Å between the metal centre and the oxygen atom of solvent waters during a 1 µs MD simulation.

Yb_2Tb – cis-cis			
$n_{(H_2O)}$	Tb	Yb1	Yb2
0	0.09 %	99.97 %	99.97 %
1	32.91 %	0.03 %	0.03 %
2	66.99 %	-	-
3	0.02 %	-	-

Table S5. Number of directly coordinated waters to each metal centre in Yb_2Tb cis-trans conformation calculated with a cut-off of 2.7 Å between the metal centre and the oxygen atom of solvent waters during a 1 µs MD simulation.

Yb_2Tb – cis-trans			
$n_{(H_2O)}$	Tb	Yb1	Yb2
0	0.28 %	99.97 %	99.97 %
1	39.47 %	0.03 %	0.03 %
2	51.72 %	-	-
3	8.53 %	-	-

Table S6. Intermetallic distances from MD simulations in the three conformations of Yb_2Tb. Distances were obtained from the maximum of fitted normal distributions of the histograms in Figures S35-37.

	trans (Å)	cis-cis (Å)	cis-trans (Å)
Yb-Yb	13.5	25	15
Yb1-Tb	11.5	13.5	13.5
Yb2-Tb	12	13.5	12

Table S7. Intermetallic distances from DFT calculations in the three conformations of Yb_2Tb.

	trans (Å)	cis-cis (Å)	cis-trans (Å)
Yb-Yb	15.3	26.5	21.0
Yb1-Tb	7.8	13.4	13.5
Yb2-Tb	12.2	13.2	12.8

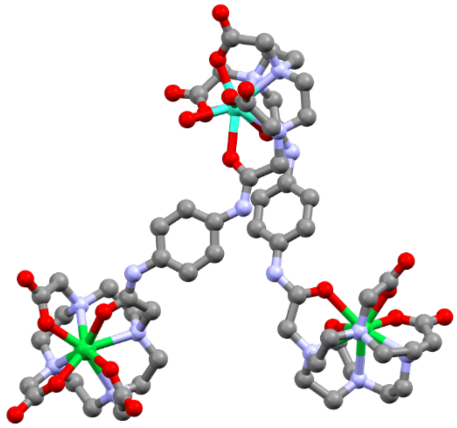

Yb$_2$Tb(trans): frame 53408
Yb-Yb: 12.235 Å

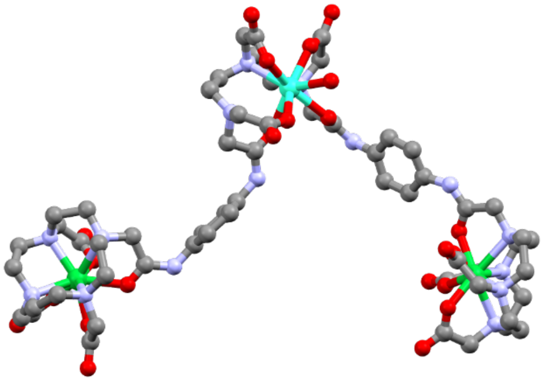

Yb$_2$Tb(cis-cis): frame 21192
Yb-Yb: 18.437 Å

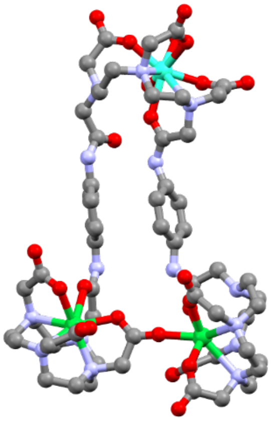

Yb$_2$Tb(cis-trans): frame 54451
Yb-Yb: 6.429 Å

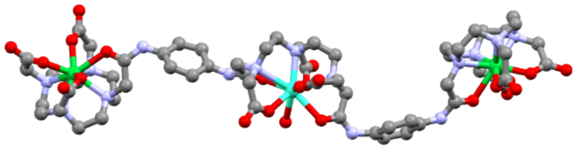

Yb$_2$Tb(cis-cis): frame 85529
Yb-Yb: 25.859 Å

Figure S51. Snapshots of the MD trajectories. Hydrogens have been removed for clarity.

Table S8. Yb^{3+} and Tb^{3+} ion parameters for the TIP3P water model during MD simulations.

	Yb^{3+}	Tb^{3+}
σ (kJ/mol)	0.2950	0.2904
ε (nm)	0.1674	0.3979

Table S9. Population of complexes with both Yb1-Tb and Yb2-Tb within cutoff distances between 9 and 15 Å from MD simulations. All individual intermetallic distances are shown in Figures S35-37.

	Population		
Cutoff (Å)	trans	cis-cis	cis-trans
9	0.00 %	0.00 %	0.00 %
10	0.04 %	0.00 %	0.00 %
11	3.17 %	0.00 %	0.00 %
12	37.69 %	0.02 %	0.11 %
13	94.06 %	6.28 %	16.60 %
14	99.98 %	67.00 %	86.75 %
15	100.00 %	94.45 %	99.98 %

References

1. Thornton, M. E.; Hemsworth, J.; Hay, S.; Parkinson, P.; Faulkner, S.; Natrajan, L. S. Heterometallic lanthanide complexes with site-specific binding that enable simultaneous visible and NIR-emission. *Frontiers in Chemistry* **2023**, *11*. DOI: 10.3389/fchem.2023.1232690.
